# Accessing Diverse Pyridine-Based Macrocyclic Peptides by a Two-Site Recognition Pathway

**DOI:** 10.1021/jacs.2c02824

**Published:** 2022-06-17

**Authors:** Dinh T. Nguyen, Tung T. Le, Andrew J. Rice, Graham A. Hudson, Wilfred A. van der Donk, Douglas A. Mitchell

**Affiliations:** † Department of Chemistry, University of Illinois at Urbana−Champaign, Urbana, Illinois 61801, United States; ‡ Carl R. Woese Institute for Genomic Biology, University of Illinois at Urbana−Champaign, Urbana, Illinois 61801, United States; § Howard Hughes Medical Institute, University of Illinois at Urbana−Champaign, Urbana, Illinois 61801, United States

## Abstract

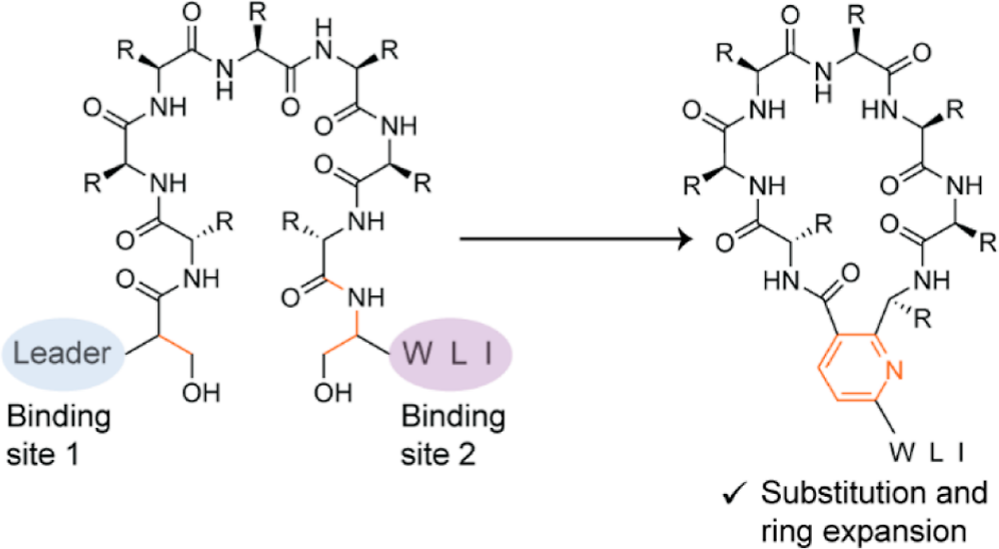

Macrocyclic peptides are sought-after molecular scaffolds for drug discovery, and new methods to access diverse libraries are of increasing interest. Here, we report the enzymatic synthesis of pyridine-based macrocyclic peptides (pyritides) from linear precursor peptides. Pyritides are a recently described class of ribosomally synthesized and post-translationally modified peptides (RiPPs) and are related to the long-known thiopeptide natural products. RiPP precursors typically contain an N-terminal leader region that is physically engaged by the biosynthetic proteins that catalyze modification of the C-terminal core region of the precursor peptide. We demonstrate that pyritide-forming enzymes recognize both the leader region and a C-terminal tripeptide motif, with each contributing to site-selective substrate modification. Substitutions in the core region were well-tolerated and facilitated the generation of a wide range of pyritide analogues, with variations in macrocycle sequence and size. A combination of the pyritide biosynthetic pathway with azole-forming enzymes was utilized to generate a thiazole-containing pyritide (historically known as a thiopeptide) with no similarity in sequence and macrocycle size to the naturally encoded pyritides. The broad substrate scope of the pyritide biosynthetic enzymes serves as a future platform for macrocyclic peptide lead discovery and optimization.

## Introduction

Macrocyclic peptide natural products are a privileged class with many members exhibiting potent antibacterial, antifungal, antiviral, anticancer, and immunosuppressive activities.^1,2^ Compared to their linear counterparts, macrocyclic peptides possess desired properties, such as proteolytic stability, increased cell-membrane permeability, and conformational restrictions, resulting in reduced entropy cost upon binding biological targets.^3,4^ These features have increased interest in accessing macrocyclic peptides through combinatorial display,^5^ epitope grafting,^6^ and cyclization of previously identified linear peptides with activity against biological targets.^7^ These efforts are greatly aided by versatile macrocyclization methods that tolerate a wide variety of peptide sequences and that can be executed with large-sized libraries.^8−10^

Ribosomally synthesized and post-translationally modified peptides (RiPPs) routinely have macrocyclic structures.^11^ During RiPP biosynthesis, a gene-encoded precursor peptide undergoes modification by enzymes encoded in a biosynthetic gene cluster (BGC). RiPP precursor peptides are commonly composed of an N-terminal leader region responsible for recruiting biosynthetic proteins and a C-terminal core region that undergoes conversion to the mature RiPP. The physical separation of substrate binding from the site(s) of modification is an attractive feature of RiPP biosynthesis, as it facilitates access to a chemically diverse array of variants. Thus, libraries based on RiPP macrocyclic peptides have been constructed to yield analogues with reprogrammed bioactivity.^12−16^

Thiopeptides are macrocyclic RiPPs associated with several enticing bioactivities of which potent inhibition of bacterial protein translation is the best studied.^17^ Structural analysis of thiopeptides reveals three universal functional groups: azole/azoline heterocycles derived from the ATP-dependent backbone cyclodehydration of Cys, Ser, and Thr residues;^18^ dehydroalanine/dehydrobutyrine (Dha/Dhb) and their derivatives resulting from the glutamylation and subsequent elimination of Ser and Thr residues;^19,20^ and a class-defining, six-membered nitrogenous heterocycle resulting from a formal [4 + 2] cycloaddition of two Dha-like residues that coincide with elimination of water and the leader peptide.^21^ Accessing thiopeptide derivatives beyond single amino acid substitutions has been challenging because of the requirement of multiple azoles in the peptide for downstream Dha formation and [4 + 2] cycloaddition.^17,18,22−26^ The only thiopeptide thus far shown to be amenable to multisite variation is lactazole, for which macrocyclization requires only two azoles and three Dha residues.^27^

**Figure 1:**
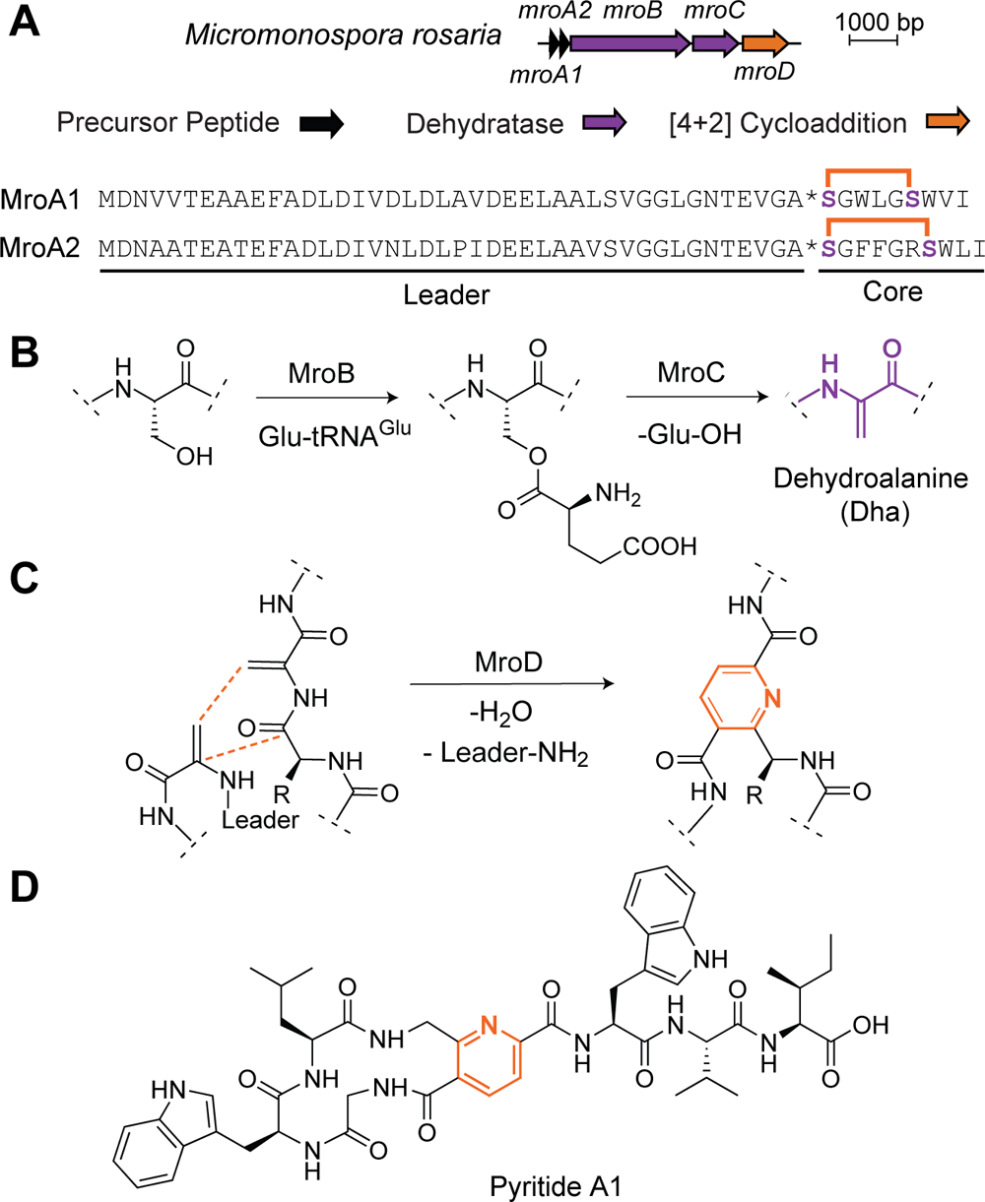
Biosynthesis of pyritides. (A) BGC from *Micromonospora rosaria* and sequences of precursor peptides. (B) Reactions catalyzed by MroB and MroC. (C) Reaction catalyzed by the [4 + 2] macrocyclase MroD. (D) Structure of pyritide A1 with the class-defining pyridine shown in orange.

Recently, we reported a minimalistic, thiopeptide-like BGC from *Micromonospora rosaria* that encodes two precursor peptides without Cys residues. The BGC also lacks the genes for azol(in)e formation^28^ and was predicted to produce a pyridine-based macrocyclic peptide (*i.e.*, pyritide, Figure 1). Reasoning that the absence of thiazol(in)es would render the pyritide biosynthetic pathway more tolerant of substitutions in the core region, we investigated here the substrate selectivity of pyritide biosynthesis to contribute to recent efforts to identify macrocycle-forming biosynthetic enzymes with broad substrate tolerance.^11,13,14,29−36^

## Results and Discussion

### Reconstitution of Enzymatic Pyritide Production

In previous work, native pyritides were accessed *via* total chemical synthesis or enzymatic [4 + 2] cycloaddition using a substrate peptide with chemically installed Dha residues.^28^ Here, to facilitate understanding of the substrate scope of the entire pathway, we focused on the complete enzymatic biosynthesis of pyritides. We first reconstituted the activity of MroB and MroC, a split LanB-like dehydratase pair that forms two Dha residues in the MroA precursor peptides (Figure 1).^19,20^ Based on membership in InterPro family IPR006827, which includes both dehydratases and enzymes with other tRNA-dependent activities,^37^ MroB (NCBI accession identifier WP_067368389.1) was expected to utilize Glu-tRNA^Glu^ to glutamylate the side chain of Ser residues. MroC (IPR023809, WP_083978639.1) was expected to eliminate glutamate to yield Dha. To test this hypothesis, the genes encoding MroB and MroC were cloned and expressed in *Escherichia coli* with maltose-binding protein (MBP) fused to the N-terminus of each protein. MBP-MroB and MBP-MroC were purified using affinity and size-exclusion chromatography (Supporting Information, Figure S1). MBP-MroB was only successfully purified after co-expression with *Thermobispora bispora* GluRS and tRNA^Glu^(CUC) (Figure S1), which shares 91% sequence identity with *M. rosaria* tRNA^Glu^(CUC) (Table S4). After purification, the precursor peptides MroA1 and MroA2 were reacted with MBP-MroB and MBP-MroC in the presence of ATP, L-Glu, *T. bispora* GluRS, and tRNA^Glu^(CUC). Analysis by matrix-assisted laser desorption/ionization time-of-flight mass spectrometry (MALDI-TOF-MS) and high-resolution electrospray ionization tandem mass spectrometry (HR-ESI-MS/MS) indicated that Ser1 and Ser6/Ser7 (MroA1/MroA2) were dehydrated (Figures S2–S5). Omission of MBP-MroC showed the formation of diglutamylated intermediates of MroA1 and MroA2 (Figures S2 and S3). Didehydrated MroA1 and MroA2 were then treated with MBP-MroD (like MroC, a member of IPR023809; WP_067368384.1), yielding the expected pyritides and elimination of the leader peptide as a C-terminal carboxamide (leader-NH_2_, Figures S2, S3, S6, and S7). The high-performance liquid chromatography and MS/MS profiles of enzymatically prepared pyritide A1 and pyritide A2 matched their corresponding standards whose structures were previously verified by ^1^H NMR spectroscopy (Figures S8–S11).^28^

### Tolerance of the Pyritide Biosynthetic Machinery toward Single-Site Variation

**Figure 2:**
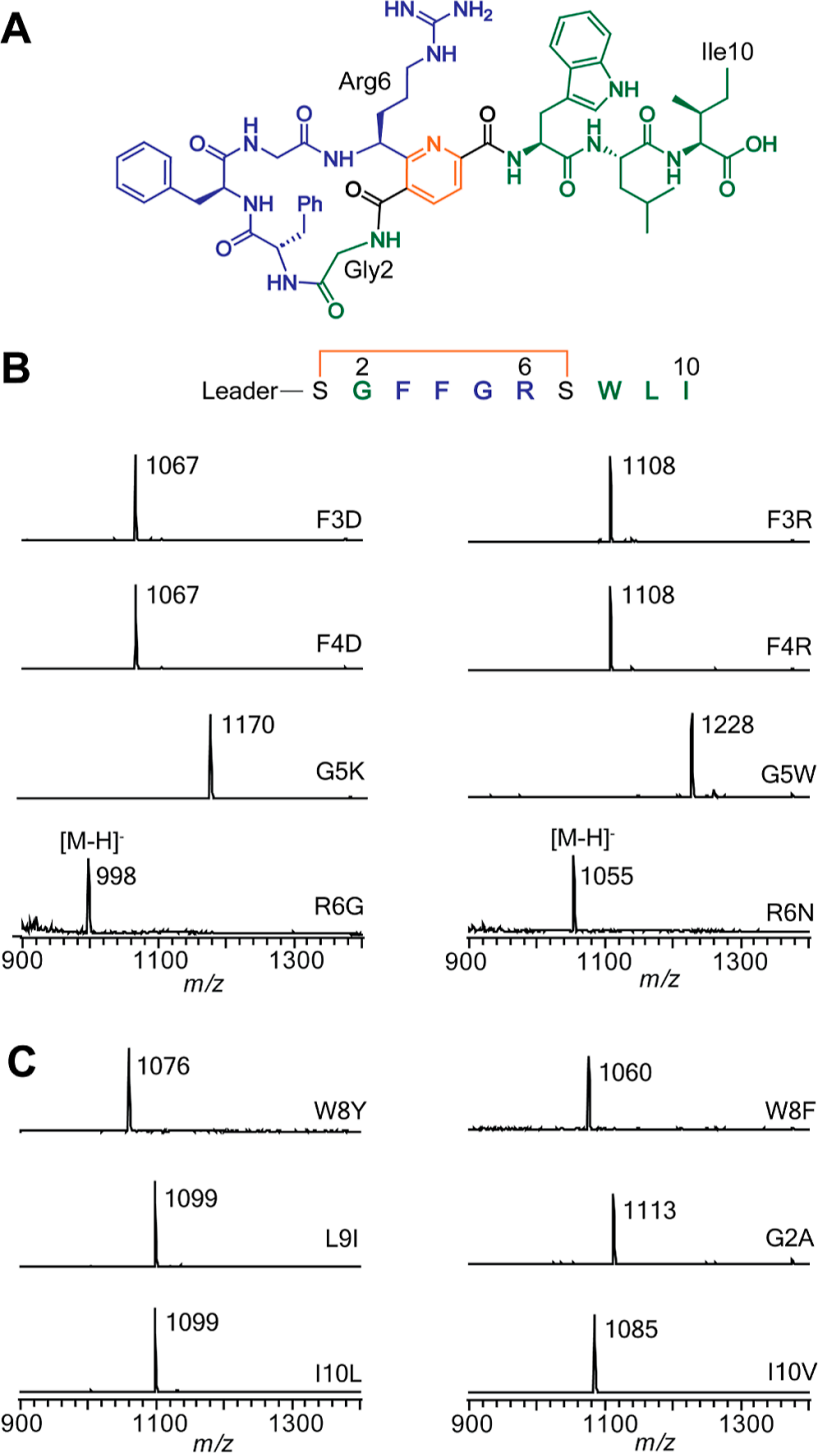
Substrate scope of MroBCD. Unless otherwise stated, all peaks represent [M + H]^+^. (A) Summary of results from assays in which MroA2 variants reacted with MroBCD (Figures S12–S21). Highlighted in blue are residues tolerant of nonconservative substitutions for pyritide maturation. MroBCD only accepted conservative substitutions of residues highlighted in green. (B) Representative MALDI-TOF-MS of MroA2 variants at Phe3, Phe4, Gly5, and Arg6 processed by MroBCD. (C) Macrocycle formation from substrates with conservative substitutions of Gly2, Trp8, Leu9, and Ile10.

Having successfully reconstituted the enzymatic biosynthesis of pyritide A1/A2, we next examined whether residues in the core region can be substituted to generate analogues. We first varied each core position of MroA2 with amino acids of different physicochemical properties using *in vitro* transcription and translation,^38^ generating 52 single-site variants. These variants were subjected to the treatment of MroBCD, and the reaction outcomes were analyzed by MALDI-TOF-MS (Figures S12–S21, Table S5). Only conservative substitutions were well tolerated at Gly2 (G2A), Trp8 (W8Y and W8F), Leu9 (L9I in MroA2), and Ile10 (I10L and I10V) (Figure 2) for the overall pyritide biosynthesis. Other Trp8 (W8G, W8A, W8D, W8N, and W8R) and Ile10 (I10G, I10A, I10N, I10D, and I10W) variants resulted in inefficient dehydration and macrocyclization (Figures S15 and S22), while didehydrated peptides with nonconservative substitutions at Gly2 (G2D, G2L, G2N, G2W, and G2R) and Leu9 (L9D, L9R, L9G, L9W, and L9N) were poor substrates for macrocyclization. In contrast, all examined single substitutions of the ring positions (Phe3, Phe4, Gly5, and Arg6) yielded the expected macrocycle.

### Tolerance of the Biosynthetic Machinery toward Multisite Variation and Ring Expansion and Contraction

**Figure 3:**
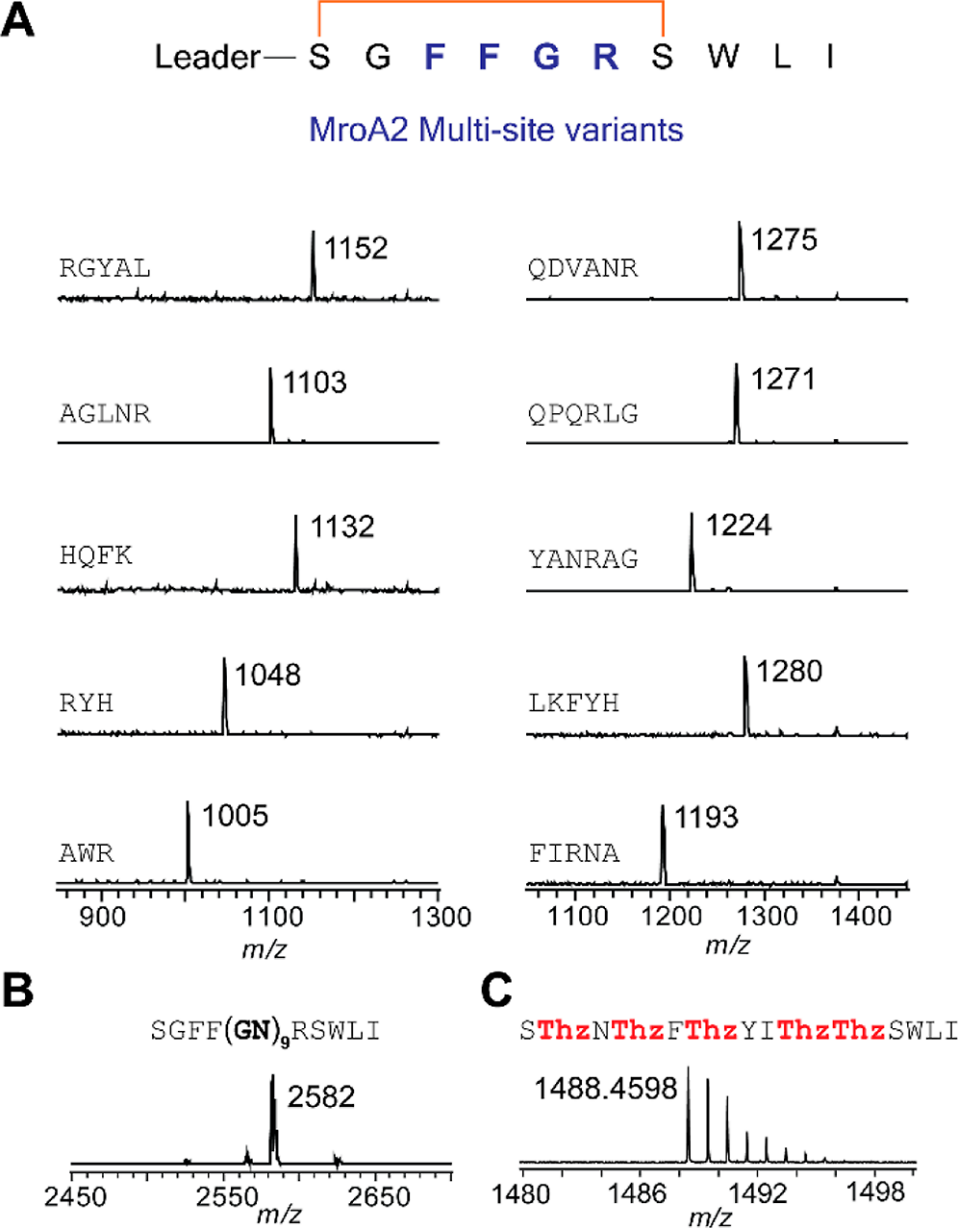
Panel of variant pyritides. Variations were made in regions in blue. (A) MALDI-TOF-MS of representative multisite pyritide variants. (B) MALDI-TOF-MS of a 68-membered pyritide macrocycle through substitution of Gly by (GlyAsn)_9_. (C) LC-HR-ESI-MS of a pyritide containing four thiazoles and one thiazoline. Thiazol(in)e residues are bolded in red and abbreviated as Thz. Additional multisite variant data are shown in Tables S5 and S6 and Figures S23–S38 and S44–S49.

Encouraged by the substrate flexibility in the ring, we next expanded the size and sequence of the macrocycle by inserting 56 different sequences varying in length from three to six residues between the two Ser residues involved in pyridine formation; Gly2 was retained (Figure 3, Table S6). These substrate variants were treated with MroBCD, and the products were analyzed by MALDI-TOF-MS (Figures S23–S28) and HR-ESI-MS/MS (Figures S29–S37). All 56 variants successfully yielded two Dha residues after treatment with MroBC, illustrating the contrast of this enzyme pair compared to dehydratases from thiopeptide BGCs that often require prior introduction of specific azoles.^24,27,39^ Reactions including MroD demonstrated that 44 out of 56 didehydrated substrates were macrocyclized (Table S6). We did not observe trends separating substrates and nonsubstrates of MroD in our data set, except the fact that all variants containing Arg or Lys immediately upstream of the C-terminal Dha (equivalent to Arg6 in MroA2) were processed. Hence, positively charged residues at this position are beneficial but not essential. To examine whether an Arg residue at this position would turn nonsubstrates into substrates, Arg was introduced in 11 peptides that previously were poor or nonsubstrates for macrocyclization (Figure S38). Six were cyclized, showing that Arg at this position contributes but is not sufficient to render any sequence a substrate. We then examined whether Thr at this position would be preferred due to its prevalence in natural variants (Table S7). In all investigated substrates, this Thr was bypassed as a site of MroBC-catalyzed dehydration, and six out of ten didehydrated Thr-containing precursors were poor or nonsubstrates for macrocyclization by MroD (Figures S39–S42). Thus, unlike Arg, Thr preceding the second Ser in the core peptide does not facilitate efficient pyritide formation by MroBCD but may be preferable for catalysis by other natural homologues. Further elucidation of the substrate tolerance of MroD will require structural information on core peptide binding. Nonetheless, our data show that whereas some positions are intolerant to variation, much of the precursor peptide tolerates a wide range of substitution, including multiple positively or negatively charged residues.

Pyritides A1 and A2 have 14- and 17-membered rings, respectively. Our substrate engineering efforts show that MroBCD can form 14–23-membered rings with diverse sequences (Table S5). We examined next whether the ring size can be further contracted or expanded. Two (Phe4 and Gly5) and three residues (Phe3, Phe4, and Gly5) could be deleted without effecting the dehydration by MroBC, but MroD did not cyclize the dehydrated intermediates to form 8- and 11-membered rings (Figure S43). Thus, the smallest ring size achieved in our data set is a 14-membered ring. Conversely, larger ring sizes were readily accessed including a pyritide macrocycle of 68 atoms *via* a 17-residue insertion of a Gly–Asn repeat, the longest attempted insertion (Figures S44–S48). Gly–Asn repeats were initially chosen due to their established usage as hydrophilic flexible linkers^40^ and were preferred in this work over popular Gly–Ser repeats^41−44^ as they may lead to extra dehydrations and potentially complicate downstream data analysis. We subsequently examined whether MroBCD tolerates large rings with sequences different from Gly–Asn repeats through randomization (Supporting Information). All investigated sequences successfully formed 62-membered macrocycles albeit didehydrated intermediates were also detected (Figure S49).

### Use of MroBCD and TbtEFG for Thiopeptide Formation

We next investigated whether post-translational modification can be performed on residues inside the pyritide macrocycle. We chose thiazole formation from Cys residues to assess the feasibility of using MroBCD as a platform for thiopeptide engineering. Thus, we inserted the core sequence of the thiomuracin macrocycle (with four C-terminal residues deleted) between the MroA1 leader peptide and the three C-terminal MroA residues (Trp–Leu–Ile) that were shown above to be important for MroBCD activity. The resulting core sequence shares no similarity with the wild-type sequence (Figure S50). In addition, in the leader peptide of this non-natural substrate, we incorporated residues previously identified as critical for the thiazole synthetase TbtEFG (NCBI accession identifier TbtE WP_013130813.1, TbtF WP_206207102.1, and TbtG WP_206207103.1).^24^ All Cys residues in the designed substrate peptide were successfully converted to thiazole/thiazoline residues after treatment with TbtEFG, and the macrocycle was formed upon reaction with MroBCD (Figures 3C, S50, and S51), opening possibilities to access diverse chemical space of both thiopeptides and pyritides.

### Mechanism of Substrate Recognition

The broad substrate tolerance, including the ability to significantly expand the size of the macrocycle, combined with the observed importance of the C-terminal tripeptide for catalysis, suggested that MroBCD relies on both the leader region and the C-terminal motif for substrate binding. We tested this hypothesis through analysis of substrate binding to MroB and MroD. Substrate binding to MroC was not investigated as glutamate elimination activity was consistently observed with the substrate variants, suggesting that elimination activity is not limiting. This finding agrees with recent reports showing that MroC homologues recognize glutamylated Ser/Thr rather than a specific peptide sequence.^39,45^ Sequence alignment of pyritide precursor peptides indicated that the first 12 residues in the leader region are not conserved and thus are unlikely to be critical for binding (Table S7). Indeed, a variant of MroA1 in which the first 12 residues were deleted (termed Δ12MroA1) underwent full dehydration and macrocyclization (Figure S52). Fluorescence polarization (FP) measurements indicated that Δ12MroA1 N-terminally labeled with fluorescein (fluorescein-Δ12MroA1) displayed high affinity toward MBP-MroB and MBP-MroD (*K*_D_ MroB ≈ 60 nM and *K*_D_ MroD ≈ 12 nM) (Figure S53). Neither the leader nor the core regions efficiently displaced the labeled precursor peptide (Table 1 and Figures S54 and S55), confirming that MroB and MroD require both for avid binding. We also investigated a panel of MroA1 variants by competition FP assays with fluorescein-Δ12MroA1 (Table 1 and Figures S54 and S55). The binding data with the variants also confirm the importance of the C-terminal tripeptide for MroB (Trp7) and MroD (Trp7, Val8, and Ile9) binding (Figures S56 and S57). To determine if the C-terminal carboxylate is important, we evaluated the binding of MroB to the methyl ester variant of Δ12MroA1, which resulted in approximately eightfold loss in binding affinity (Table 1 and Figure S56).^46^ Thus, both binding and activity data point to recognition of the leader peptide and the C-terminal tripeptide.

**Table 1:** Binding of MroA1 Variants to MroB and MroD^a^

MroA1 variants	sequence	IC_50_ MroB (μM)	IC_50_ MroD (μM)
Δ12MroA1	SDLDIVDLDLAVDEELAALSVGGLGNTEVGASGWLGSWVI	0.68 ± 0.04	0.09 ± 0.02
Δ12MroA1 leader	SDLDIVDLDLAVDEELAALSVGGLGNTEVGA	19.4 ± 1.6	16.0 ± 4.4
GlyAla-MroA1 core	Ac-GASGWLGSWVI	27.2 ± 1.9	40.9 ± 5.0
Δ12MroA1-W7G	SDLDIVDLDLAVDEELAALSVGGLGNTEVGASGWLGSGVI	8.1 ± 3.9	2.4 ± 0.3
Δ12MroA1-V8G	SDLDIVDLDLAVDEELAALSVGGLGNTEVGASGWLGSWGI	1.1 ± 0.2	1.7 ± 0.4
Δ12MroA1-I9G	SDLDIVDLDLAVDEELAALSVGGLGNTEVGASGWLGSWVG	2.0 ± 0.6	0.91 ± 0.09
Δ12MroA1-COOMe	SDLDIVDLDLAVDEELAALSVGGLGNTEVGASGWLGSWVI-COOMe	4.2 ± 1.7	0.40 ± 0.06

a FP traces and *K*_i_ values are shown in Supporting Information, Figures S53–S57. Ac = *N*-acetyl.

**Figure 4:**
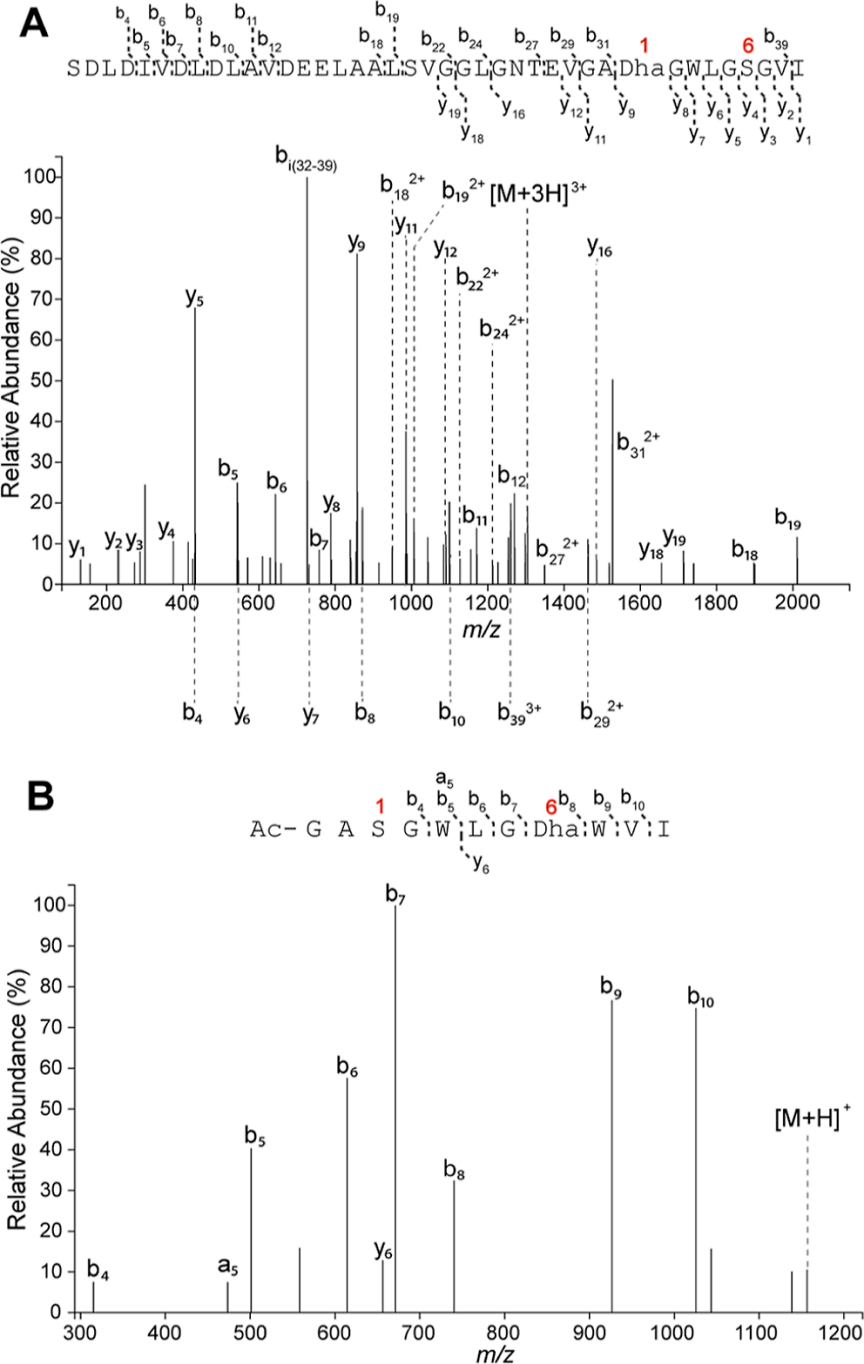
LC-ESI-MS/MS of MroA1 variants treated with MroBC. Extracted ion chromatogram traces are in Figures S58 and S59. (A) Product obtained with Δ12MroA1-W7G, showing that Ser1 was dehydrated. (B) Product obtained with the GlyAla-MroA1 core, showing that Ser6 was dehydrated.

With the support for two-site recognition by MroB, we investigated how each site contributed to the overall dehydration of MroA1 and MroA2. MroBC assays followed by LC–MS/MS analysis revealed that only Ser1 is predominantly dehydrated in Δ12MroA1 W7G, while only Ser6 is dehydrated in the GlyAla-MroA1 core peptide (Figure 4). These data suggest that the leader peptide is more important for dehydration at Ser1 and the C-terminal tripeptide is more important for dehydration at Ser6. Analogously, the MroA2 variants S7G/W8G and S7G/I10G were completely dehydrated at Ser1, whereas MroA2-S1G/W8G and MroA2-S1G/W10G were inefficiently dehydrated at Ser7 (Figure S60). Dehydration of both MroA2-S1G and MroA2-S7G went to completion, indicating that the two dehydrations are independent of one another.

In summary, we fully reconstituted enzymatic pyritide biosynthesis *in vitro*, enabling in-depth characterization of the substrate selectivity of the dehydratase MroBC and the [4 + 2] cycloaddition enzyme MroD. The enzymatic macrocyclization proved to be compatible with *in vitro* translation, presenting a powerful platform for macrocyclic peptide library construction. Our data support a model in which these enzymes recognize both the leader peptide and the C-terminal tripeptide. The leader peptide is more important for dehydration at the N-terminal Ser in the core, whereas the C-terminal tripeptide is more important for dehydration at Ser6/7. By keeping the leader peptide and C-terminal residues invariant, we generated pyritide analogues with diverse ring sequences and sizes (14–68 membered). These data will facilitate future efforts in the bioengineering of macrocyclic peptides with desirable properties.

## Supporting Information Available

Experimental procedures and supporting figures and tables (PDF)
Primer sequences (XLSX)
MS data (XLSX)
