# Supplementary material for: Accessing Diverse Pyridine-Based Macrocyclic Peptides by a Two-Site Recognition Pathway
Source: J Am Chem Soc. 2022 Jun 17;144(25):11263–9. doi: 10.1021/jacs.2c02824 (PMC9247985; doi:10.1021/jacs.2c02824)
Supplement: Supplementary file 1 — ja2c02824_si_001.pdf [file ja2c02824_si_001.pdf]

Supporting Information for:

Accessing diverse pyridine-based macrocyclic peptides by a two-site recognition pathway

Dinh T. Nguyen[†,‡,§], Tung T. Le[†,§], Andrew J. Rice[†,‡], Graham A. Hudson[†,‡], Wilfred A. van der Donk[†,‡,§*], and Douglas A. Mitchell[†,‡*]

[†]Department of Chemistry, [‡]Carl R. Woese Institute for Genomic Biology, [§]Howard Hughes Medical Institute, University of Illinois at Urbana-Champaign, Urbana, Illinois, 61801, USA.

* Corresponding authors:

Wilfred A. van der Donk (vddonk@illinois.edu), phone: 1-217-244-5360, fax: 1-217-244-8533

Douglas A. Mitchell (douglasm@illinois.edu), phone: 1-217-333-1345, fax: 1-217-333-0508

Table of Contents:

Experimental Methods....4

Table S1: Sequence of *Mro* biosynthetic genes and *T. bispora* GluRS for optimal *E. coli* expression....14

Table S2: Nucleotide sequence of open reading frames inserted into plasmids used in this study....16

Table S3: Oligonucleotide primers used in plasmid constructions for heterologous expression in *E. coli*....17

Figure S1: SDS-PAGE analysis of proteins used in this study....18

Table S4: Sequence alignment of *M. rosaria* tRNA^{Glu}(CUC) and *T. bispora* tRNA^{Glu}(CUC)....19

Figure S2: Dehydration (MroB/C) and cyclization (MroD) of the MroA1 precursor peptide....20

Figure S3: Dehydration (MroB/C) and cyclization (MroD) of the MroA2 precursor peptide....21

Figure S4: HR-ESI-MS/MS analysis of didehydrated MroA1....22

Figure S5: HR-ESI-MS/MS analysis of didehydrated MroA2....23

Figure S6: HR-ESI-MS/MS analysis of the eliminated carboxamide leader peptide of MroA1....24

Figure S7: HR/MS/MS analysis of the eliminated carboxamide leader peptide of MroA2....25

Figure S8: Analytical LC-MS analysis of enzymatically synthesized and chemically synthesized pyritide A1....26

Figure S9: Tandem MS fragmentation of enzymatically and chemically synthesized pyritide A1....27

Figure S10: Analytical LC-MS analysis of enzymatically synthesized and chemoenzymatically synthesized pyritide A2....28

Figure S11: Tandem MS fragmentation of enzymatically and chemoenzymatically synthesized pyritide A2....29

Figure S12: Phe3, Phe4, and Gly5 variants of MroA2 undergo two dehydrations after MroB/C treatment....30

Figure S13: Gly2 and Leu9 variants of MroA2 undergo two dehydrations after MroB/C treatment....31

Figure S14: Arg6, Trp8, and Ile10 variants of MroA2 undergo two dehydrations after MroB/C treatment....32

Figure S15: Gly2, Trp8, and Ile10 variants of MroA2 undergo incomplete dehydrations after MroB/C treatment....33

Figure S16: MALDI-TOF-MS analysis of Phe3 and Phe4 variants of MroA2 after MroB/C/D treatment....34

Figure S17: MALDI-TOF-MS analysis of Phe4 and Gly5 variants of MroA2 after MroB/C/D treatment....35

Figure S18: MALDI-TOF-MS analysis of Arg6 and Gly2 variants of MroA2 after MroB/C/D treatment....36

Figure S19: MALDI-TOF-MS analysis of Trp8 variants of MroA2 after MroB/C/D treatment....37

Figure S20: MALDI-TOF-MS analysis of Leu9 variants of MroA2 after MroB/C/D treatment....38

Figure S21: MALDI-TOF-MS analysis of Ile10 variants of MroA2 after MroB/C/D treatment....39

Table S5: Summary of MroB/C/D activity on MroA2 single core peptide variants....40

Figure S22: Trp8 and Ile10 variants of MroA2 MALDI-TOF-MS undergo inefficient cyclization by MroD 41
Figure S23: MALDI-TOF-MS analysis of shown MroA2 multi-site variants 1-10 after MroB/C/D assay.................42
Figure S24: MALDI-TOF-MS analysis of shown MroA2 multi-site variants 11-19 after MroB/C/D assay...............43
Figure S25: MALDI-TOF-MS analysis of shown MroA2 multi-site variants 20-29 after MroB/C/D assay...............44
Figure S26: MALDI-TOF-MS analysis of shown MroA2 multi-site variants 30-39 after MroB/C/D assay...............45
Figure S27: MALDI-TOF-MS analysis of shown MroA2 multi-site variants 40-47 after MroB/C/D assay...............46
Figure S28: MALDI-TOF-MS analysis of shown MroA2 multi-site variants 48-56 after MroB/C/D assay...............47
Table S6: Summary of MroB/C/D activity on MroA2 multi-site variants 48
Figure S29: LC-HR-ESI-MS/MS analysis of macrocyclized product of MroA2 multi-site variant 43. 50
Figure S30: LC-HR-ESI-MS/MS analysis of macrocyclized product of MroA2 multi-site variant 22. 51
Figure S31: LC-HR-ESI-MS/MS analysis of macrocyclized product of MroA2 multi-site variant 6. 52
Figure S32: LC-HR-ESI-MS/MS analysis of macrocyclized product of MroA2 multi-site variant 17. 53
Figure S33: LC-HR-ESI-MS/MS analysis of macrocyclized product of MroA2 multi-site variant 15. 54
Figure S34: LC-HR-ESI-MS/MS analysis of macrocyclized product of MroA2 multi-site variant 44. 55
Figure S35: LC-HR-ESI-MS/MS analysis of macrocyclized product of MroA2 multi-site variant 42. 56
Figure S36: LC-HR-ESI-MS/MS analysis of macrocyclized product of MroA2 multi-site variant 7. 57
Figure S37: LC-HR-ESI-MS/MS analysis of macrocyclized product of MroA2 multi-site variant 12. 58
Figure S38: MALDI-TOF-MS analysis of Arg variants of uncyclized precursor peptides after MroB/C/D treatment. 59
Table S7: Sequence alignment of pyritide precursor peptides 60
Figure S39: MALDI-TOF-MS analysis of substrates containing Thr preceding the second Ser in the core peptide after MroB/C/D treatment...61
Figure S40: Substrates containing Thr preceding the second Ser are not dehydrated at this Thr by MroB/C 62
Figure S41: LC-HR-ESI-MS/MS analysis of twice-dehydrated Thr-containing substrates in Figure S39. 67
Figure S42: LC-HR-ESI-MS/MS analysis of macrocyclized products of Thr-containing substrates in Figure S39. ..67
Figure S43: MroB/C/D do not produce 8- and 11-membered macrocycles 69
Figure S44: LC-HR-ESI-MS/MS analysis of a 32-membered macrocycle produced by MroB/C/D...........................70
Figure S45: LC-HR-ESI-MS/MS analysis of a 38-membered macrocycle produced by MroB/C/D...........................71
Figure S46: LC-HR-ESI-MS/MS analysis of a 62-membered macrocycle produced by MroB/C/D...........................72
Figure S47: LC-HR-ESI-MS/MS analysis of a 68-membered macrocycle produced by MroB/C/D...........................73
Figure S48: Large macrocycle sizes produced by MroB/C/D 74
Figure S49: MroB/C/D produces 62-membered macrocycles with different sequences...75
Figure S50: MroBCD produce a pyritide containing thiazol(in)es..76
Figure S51: LC-HR-ESI-MS analysis of the thiazol(in)es-containing pyritide and ejected leader peptide (carboxamide) 77
Figure S52: Δ12MroA1 is dehydrated by MroB/C and cyclized by MroD 78
Figure S53: Fluorescence polarization of MBP-MroB and MBP-MroD binding to fluorescein-Δ12MroA1 79
Figure S54: Both leader and core region are required for MroB binding..80
Figure S55: Both leader and core region are required for MroD binding 81
Figure S56: The C-terminal tripeptide motif is important for MroB binding..82

Figure S57: The C-terminal tripeptide motif is important for MroD binding 83
Figure S58: Extracted ion chromatogram (EIC) of the LC-MS trace of Δ12MroA1 W7G after MroB/C reaction. 84
Figure S59: Extracted ion chromatogram (EIC) of the LC-MS trace of GlyAla-MroA1 core peptide after MroB/C reaction. 85
Figure S60: Trp8 and Ile10 are important for dehydration of Ser7 in MroA2. 86
Supporting References..........87

Experimental Methods

General materials and methods. Reagents used for molecular biology experiments were purchased from New England BioLabs (NEB) (Ipswich, MA), Thermo Fisher Scientific (Waltham, MA), or Gold Biotechnology Inc. (St. Louis, MO). Other chemicals were purchased from Sigma-Aldrich (St. Louis, MO). *Escherichia coli* DH5α and BL21 (DE3) strains were used for plasmid maintenance and protein overexpression, respectively. Plasmid inserts were sequenced at ACGT Inc. (Wheeling, IL). Matrix-assisted laser desorption/ionization time-of-flight mass spectrometry (MALDI-TOF-MS) analysis was performed using a Bruker UltrafleXtreme MALDI TOF-TOF mass spectrometer (Bruker Daltonics) at the University of Illinois School of Chemical Sciences Mass Spectrometry Laboratory. MALDI-TOF-MS samples were desalted prior to analysis by using a C18 ZipTip (EMD Millipore) prior to co-crystallization in a suitable matrix. High-resolution electrospray ionization (HR-ESI) MS/MS analyses were performed using a ThermoFisher Scientific Orbitrap Fusion ESI-MS using an Advion TriVersa Nanomate 100. Tables with theoretical and observed masses for peptides and peptide fragments are provided in Supplementary Dataset 2. Liquid chromatography coupled with ESI-MS/MS (LC-ESI-MS/MS) was performed using a 6545B LC/Q-TOF MS purchased from Agilent. Lyophilization was performed using a Labconco (Kansas City, MO) freeze dryer.

Molecular biology techniques for generation of plasmids encoding precursor peptides and proteins. Oligonucleotides were purchased from Integrated DNA Technologies Inc. (Coralville, IA). Sequences of primers used in this study are provided in **Supplementary Dataset 1** and **Table S3**. Genes optimized for recombinant expression in *Escherichia coli* were synthesized by Twist Bioscience in pET28 (kanamycin, Kan) vectors with BamHI and XhoI sites flanking each gene at the 5′ and 3′ ends, respectively. The GenBank locus tag and *E. coli* optimized sequence for each gene is provided in **Table S1**.

Our general cloning strategy involved generating DNA inserts containing DNA template encoding desired peptide-protein, respectively, and a plasmid backbone for growth in *E. coli*. Primers used in cloning are summarized in Table S3. The DNA inserts were generated by PCR (50 µL scale, 34 cycles of 95 °C denaturation 30 s, annealing 30 s, and 72 °C extension 30 s) using a Q5 polymerase kit purchased from NEB, with annealing temperature calculated from NEB T_m calculator. Most expression vectors derive from a modified pET28 backbone that fuses maltose-binding protein (MBP) to the N-terminus of the protein of interest. The 5' BamHI and 3' XhoI restriction sites were used for plasmid linearization with the exception of *mroB*, where Q5 PCR described above (extension time 5 min) with primer F-Backbone and R-Backbone (**Table S3**) was used. The amplified DNA inserts and digested plasmid vectors were purified using agarose gel electrophoresis [0.7% (*w/v*)] followed by gel extraction (GeneJET).

The vectors and inserts were ligated using Gibson ligation[1] or T4 DNA ligase. If the primer used to generate inserts create BamHI and XhoI sites (**Table S3**), ligation was done using T4 DNA ligase (NEB). Otherwise, ligation was achieved using Gibson Assembly Master Mix (NEB) at 50 °C for 1 h. Ligation reactions were used to transform chemically competent DH5α cells, which were then plated on Luria-Bertani (LB) agar plates containing 50 µg/mL kanamycin and grown at 37 °C. Colonies were picked at random and grown in LB broth for 12-15 h before plasmid isolation using GeneJET Plasmid Miniprep Kit. For all MBP-Tagged precursor peptides and proteins used in this study (except for TbtE), the vector encoded a tobacco etch virus (TEV) protease-cleavable site (ENLYFQS) at the N-terminus of the peptides and proteins. All recombinant constructs featuring an MBP-tag were sequenced using a custom MBP forward primer and reverse-sequenced using a T7 reverse primer as well as an internal primer when necessary (**Table S3**).

mroA2-W8G and *mroA2-I10G* were generated by site-directed mutagenesis using the QuikChange method (Agilent) on the plasmid pET28-MBP-MroA2(S1C/S7C) developed previously.[2]

Generation of *mroA* variants templates DNA for *in vitro* translation. Linear double-stranded DNA encoding a T7 promoter and ribosome binding site upstream of the *mroA* open reading frame and mutants were synthesized by one- or multiple-step PCR from single-stranded DNA oligonucleotides using Taq polymerase (NEB). The PCR contains 10 mM Tris-HCl pH 8.3, 50 mM KCl, 1.5 mM $MgCl_2$, 200 μM each dNTPs, and 1 μM of the appropriate forward and reverse primers. The forward and reverse primers for each template DNA preparation are described in Supplementary Dataset 1.

Generally, the protocol involved three different PCR steps: 1) Primer extension; 2) 5-cycle PCR for lengthening DNA template (multiple PCRs were performed for long DNA template, according to Supplementary Dataset 1); 3) Final PCR to amplify the final PCR product that will be used for *in vitro* translation.

The first step involved primer extension to create an extension product with T7 promoter and RBS upstream of *mro*A1 leader (ExtPrimerF1). Specifically, the primer ExtPrimerF1 was mixed with Leader.R1 in the PCR mixture (100 μL scale), denatured at 95 °C (1 min) in 1 cycle, followed by 5 cycles of 54 °C annealing (1 min) and 72 °C extension (1 min).

The 5-cycle PCR was done as follows: The extension product was diluted 200-fold by the polymerase mixture and amplified using the respective forward and reverse primers (1 μM final concentration each) in a 50 μL reaction. After primer addition, the mixture was subjected to 5 cycles of 95 °C denaturation (40 s), 61 °C annealing (40 s, and 72 °C extension (40 s). Multiple 5-cycle PCRs were needed for long DNA templates, according to Supplementary Dataset 1.

The final PCR was done as follows: The resulting PCR product from 5-cycle PCRs was diluted 200-fold by the polymerase mixture followed by the addition of the appropriate forward and reverse primers (1 μM in final concentration each) in a 100 μL scale reaction. The new PCR mixture was then subjected to a final PCR reaction with 30 cycles of 95 °C denaturation (40 s), 61 °C annealing (40 s), and 72 °C extension (40 s). The final PCR reaction was carried out directly after the extension reaction for DNA templates that required only one-step PCR. For sequences Leader-SGRGKIQASWLI to Leader-SGANGVKTAWLI (Row 143 to Row 151, sheet MroA2variant-PCR, Supplementary Dataset 1), the final PCRs were done with the following cycle instead: 5 cycles of 95 °C denaturation (40 s), 54 °C annealing (40 s), and 72 °C extension (40 s), followed by 30 cycles of 95 °C denaturation (40 s), 61 °C annealing (40 s), and 72 °C extension (40 s).

The amplified DNA template was purified by ethanol precipitation. Specifically, in a 100 μL PCR, 10 μL of 3 M NaCl and 220 μL of EtOH was added, left on ice for 1 h, and subjected to centrifugation at 13,000 × g for 20 min at 4 °C. The supernatant was removed, and 500 μL of 70% EtOH was added to the resulting pellet, followed by centrifugation at 13,000 × g for 10 min at 4 °C. The supernatant was removed entirely, and the resulting pellet was dried by opening the cap of the Eppendorf tube (loosely covered by a Kimwipe) for 10 min. H_2O (10 μL) was then used to dissolve the DNA pellet, and this DNA solution was used for *in vitro* transcription/translation reactions.

MBP-tagged peptide overexpression and purification. *E. coli* BL21(DE3) cells were transformed with a pET28 plasmid encoding the MBP-tagged peptide of interest. Cells were grown for 14-16h on LB agar plates containing 50 μg/mL kanamycin at 37 °C. Single colonies were used to inoculate 10 mL of Terrific Broth (24 g/L yeast extract, 12 g/L tryptone, 0.4% glycerol (v/v), 17 mM KH_2PO_4, and 72 mM K_2HPO_4) containing 50 μg/mL kanamycin and grown at 30 °C for 14-18 h. This culture was used to inoculate 1 L of Terrific Broth (TB) containing 50 μg/mL kanamycin and grown to an optical density at 600 nm (OD_{600}) of 1.5-1.7. Protein expression was induced by addition of 0.4 mM isopropyl β-D-1-thiogalactopyranoside (IPTG, final) for 16 h at 16 °C. At the time of induction, the culture was also supplemented with 2 mM $MgCl_2$ and 100 μg/mL $FeSO_4.7H_2O$ as final concentrations. Cells were harvested by

centrifugation at 4,500 × *g* for 15 min, washed with phosphate-buffered saline (PBS; 137 mM NaCl, 2.7 mM KCl, 10 mM Na_2HPO_4, and 1.8 mM KH_2PO_4), and subjected to a second round of centrifugation. The cell pellet was flash-frozen and stored at -80 °C for a maximum of two weeks before use.

Cell pellets were resuspended in lysis buffer (50 mM HEPES-NaOH pH 7.5, 500 mM NaCl, 5% glycerol (*v/v*), and 0.1% Triton X-100) containing 4 mg/mL lysozyme, 2 μM leupeptin, 2 μM benzamidine, and 2 μM E64 in 50 mL tubes. The tubes were placed in an ice-water bath, homogenized by sonication (30 s on, 10 s off, continued with another 30 s on, followed by 10 min periods of gentle rocking at 4 °C). Sonication was repeated another two rounds for a total of three. Insoluble cellular debris was removed by centrifugation at 30,000 × g for 30 min. The supernatant was then applied to a pre-equilibrated Ni-NTA resin (His-Pur, Thermo Scientific, 2 mL of resin per L of cells). The column was washed with 10 column volumes (CV) of lysis buffer followed by 15 CV of wash buffer (50 mM HEPES-NaOH pH 7.5, 1M NaCl, 30 mM imidazole, 5% glycerol). The MBP-tagged peptides were eluted using 6 CV of elution buffer (50 mM HEPES-NaOH pH 7.5, 300 mM NaCl, 250 mM imidazole, 5% glycerol. The eluent was concentrated using a 30 kDa molecular weight cut-off (MWCO) Amicon Ultra centrifugal filter (EMD Millipore) and buffer-exchanged into protein storage buffer [(50mM HEPES pH 7.5, 300 mM NaCl, 2.5% glycerol (*v/v*)] using a PD-10 (Cytiva Life Sciences). Protein concentrations were estimated using 280 nm absorbance (theoretical extinction coefficients were calculated using the ExPASy ProtParam tool; http://web.expasy.org/protparam/protpar-ref.html). For cysteine-containing precursors, lysis, wash, elution, and storage buffers were supplemented with 0.5 mM tris-(2-carboxyethyl)-phosphine (TCEP).

Purification of precursor peptides after affinity chromatography. MBP-tagged precursor peptides (in 50 mM HEPES, 300 mM NaCl, 2.5% glycerol) were treated with TEV protease(L56V/S135G/S219V)[5] (50 mM HEPES, 300 mM NaCl, 2.5% glycerol, and 0.5 mM TCEP) with a 10:1 substrate to protease ratio at room temperature for 1 h. The mixture was then loaded to a C_{18} solid-phase extraction column (HyperSep C18 cartridges, Thermo Scientific) that was preequilibrated using 5 CV of acetonitrile and 5 CV of 20 mM NH_4OAc. The column was washed with 5 CV of 20 mM NH_4OAc before eluting with 80% acetonitrile, 4 mM NH_4OAc. For the 2000 mg and 5000 mg columns, 15 and 25 mL of elution were used, respectively. The collected eluant was then lyophilized and dissolved in 10-15 mL 150 mM NH_4HCO_3, subjected to centrifugation at 18,000 × g for 20 min at room temperature to remove any insoluble debris before injecting on an HPLC equipped with a preparative C_{18} column. (VP HPLC column (preparative), NUCLEODUR C18 HTec, 5 μm, 250 × 10 mm). Solvent A was 20 mM NH_4OAc while solvent B was acetonitrile. The gradient was as follows: 2-30% B in 5 min, 30-70% B in 20 min, 70-2% B in 1 min, 2% B in 5 min before ending the run. The desired fractions were collected, lyophilized, resuspended in H_2O, vortex, and lyophilized again to remove any residual NH_4OAc. Before further use in FP or *in vitro* assays, the lyophilized powder was dissolved in 0.5x storage buffer (25 mM HEPES, 150 mM NaCl, 1.25% glycerol, and 0.25 mM TCEP, pH 7.5). The concentration of each peptide was assayed using 280 nm absorbance (theoretical extinction coefficients were calculated using the ExPASy ProtParam tool; http://web.expasy.org/protparam/protpar-ref.html) or Pierce Quantitative Colorimetric Peptide Assay (Thermo Scientific)

MBP-tagged MroB overexpression and purification. *E. coli* BL21(DE3) cells were transformed with pET28-MBP-tagged MroB and a pTrc33 plasmid encoding GluRS and three copies of $tRNA^{Glu}$ (CUC) from *Thermobispora bispora* bearing a chloramphenicol marker. The GluRS and each copy of $tRNA^{Glu}$ gene were preceded by a T7 promoter. Cells were for grown for 16-18 h on LB agar plates containing 50 μg/mL kanamycin and 25 μg/mL chloramphenicol at 37 °C. Single colonies were used to inoculate 10 mL of LB or TB containing 50 μg/mL kanamycin and 25 μg/mL chloramphenicol and grown at 30 °C for 14-18 h. This culture was used to inoculate 1 L of LB or TB containing 50 μg/mL kanamycin and 25 μg/mL chloramphenicol grown to an optical density at 600 nm (OD_{600}) of 0.6-0.8 for LB and 1.5-1.7 for TB. Protein expression was induced by adding 0.5 mM IPTG and supplemented with 2 mM $MgCl_2$ as the final concentrations and proceeded for 18 h at 18 °C.

Cell pellets were resuspended in lysis buffer containing 4 mg/mL lysozyme, 2 μM leupeptin, 2 μM benzamidine, and 2 μM E64 on 50 ml falcon tubes. Cells on the falcon tubes were then put on an ice-water bath, homogenized by sonication (25 s on, 10 s off, continued with another 25 s on, followed by 10 min nutation periods at 4 °C). The sonication was repeated another two times, resulting in a total of three times sonication. For cultures larger than 3 L, the cells were lysed using a high-pressure homogenizer (Avestin, Inc.). Insoluble debris was removed by centrifugation at 30,000 × *g* for 30 min. The supernatant was then applied to a pre-equilibrated Ni-NTA resin (His-Pur, Thermo Scientific, 1 mL of resin per L of cell cultures). The column was washed with 10 column volumes (CV) of lysis buffer containing 0.5 mM TCEP, followed by 16 CV of wash buffer 1 (50 mM HEPES-NaOH pH 7.5, 1M NaCl, 30 mM imidazole, 5% glycerol, 0.5 mM TCEP). The Ni-NTA column was then further washed by 5 CV of wash buffer 2 (50 mM HEPES-NaOH pH 7.5, 300 mM NaCl, 50 mM imidazole, 5% glycerol, 0.5 mM TCEP). MBP-MroB was eluted from the column twice, first time using 5 CV of pre-elution buffer (50 mM HEPES-NaOH pH 7.5, 300 mM NaCl, 125 mM imidazole, 5% glycerol, 0.5 mM TCEP) and 5 CV of elution buffer (50 mM HEPES-NaOH pH 7.5, 300 mM NaCl, 250 mM imidazole, 5% glycerol, 0.5 mM TCEP). As the fraction from the elution buffer contained less impurity visualized through SDS-PAGE gel, this fraction was concentrated further using a 30 kDa MWCO Amicon Ultra centrifugal filter (EMD Millipore). A buffer exchange with 1000× volume of protein storage buffer (50mM HEPES pH 7.5, 300 mM NaCl, 2.5% glycerol (v/v), 0.5 mM TCEP) was performed. The buffer-exchanged protein batch was further purified with size exclusion chromatography by injecting it to an AKTA FPLC system equipped with a HiLoad 16/60 Superdex 200 pg column purchased from Cytiva Life Sciences. The column was preequilibrated and ran in the protein storage buffer. All fractions containing the proteins were identified using SDS-PAGE gel, collected, and concentrated using 30 kDa MWCO Amicon Ultra centrifugal filter (EMD Millipore) to 20 mg/mL. Protein concentrations were assayed using 280 nm absorbance (theoretical extinction coefficients were calculated using the ExPASy ProtParam tool; http://web.expasy.org/protparam/protpar-ref.html)

MBP-tagged MroC overexpression and purification. *E. coli* BL21(DE3) cells were transformed with pET28-MBP-tagged MroC and a chloramphenicol-resistant pACYC-Duet plasmid containing Cpn10 and Cpn60, which are chaperones from *Oleispira antarctica.*[3] Cells were for 16-18 h on LB agar plates containing 50 μg/mL kanamycin and 25 μg/mL chloramphenicol at 37 °C. Single colonies were used to inoculate 10 mL TB containing 50 μg/mL kanamycin and 25 μg/mL chloramphenicol grown at 30 °C for 14-18 h. This culture was used to inoculate 1 L of TB containing 50 μg/mL kanamycin and 25 μg/mL chloramphenicol grown to an optical density at 600 nm (OD_{600}) of 0.6-.0.8 for LB and 1.5-1.7 for TB. The expression was then induced by adding 0.5 mM IPTG and supplemented with 2 mM $MgCl_2$ as the final concentrations, and proceeded for 18 h at 18 °C.

The purification of MBP-MroC was done similarly to MBP-MroB with a few modifications. After the first wash with 10 CV of lysis buffer, the second wash was only done with 10 CV instead of 16 CV of wash buffer 1. 10 CV of chaperone-wash buffer (50 mM HEPES-NaOH pH 7.5, 10 mM $MgCl_2$, 7.5 mM ATP, and 150 mM KCl) was then applied to the column. The column was then allowed to nutate at 4 °C on a nutator for 2 h to break the interaction between the chaperone and MBP-MroC.[4] 10 CV of wash buffer was then applied, followed by 5 CV of pre-elution buffer and 6 CV of elution buffer. Three rounds of buffer exchange from elution buffer to protein storage buffer (50 mM HEPES pH 7.5, 300 mM NaCl, 2.5% glycerol (v/v), 0.5 mM TCEP) using 10× volume of protein storage buffer in each round was performed before size exclusion chromatography by Amicon 30 kDa 15 mL. The buffer-exchanged protein batch was further purified with size exclusion chromatography in a similar manner as MBP-MroB.

MBP-tagged MroD overexpression and purification. *E. coli* BL21(DE3) cells were transformed with pET28-MBP-tagged MroD and a chloramphenicol-resistant pACYC-Duet plasmid containing Cpn10 and Cpn60, which are chaperones from *Oleispira antarctica.*[3] The expression, affinity chromatography, and SEC were performed similar to MBP-MroC, but without applying chaperone-wash or pre-elution buffer. The wash steps included 10 CV of lysis

buffer, 15 CV of wash buffer, and elution was done using 6 CV of elution buffer.

Expression and purification of *T. bispora* GluRS. *E. coli* BL21(DE3) cells were transformed with pRSF-His6-*T. bispora* GluRS plasmid bearing a kanamycin-resistant marker. Expression and affinity chromatography was done similarly to MBP-tagged precursor peptide. Every buffer in this purification contained 0.5 mM TCEP.

Expression and purification of TEV protease. *E. coli* BL21(DE3) cells were transformed with pK793-TEV (L56V/S135G/S219V) plasmid bearing an ampicillin-resistant marker.[5] Expression and affinity chromatography were done similarly to MBP-tagged precursor peptide, with 100 µg/mL of ampicillin or carbenicillin used instead of kanamycin. Every buffer in this purification contained 0.5 mM TCEP. The overnight preculture was subjected to centrifugation of 4000 × *g* for 15 min, and supernatant (media) was removed, followed by resuspension in the same amount of fresh TB media prior to expression.

Expression and purification of MBP-LahS$_B$. *E. coli* BL21(DE3) cells were transformed with pET28a-MBP-LahS$_B$ plasmids bearing a kanamycin-resistant marker.[6] Expression and affinity chromatography were done similarly to MBP-tagged precursor peptide. Every buffer in this purification contained 0.5 mM TCEP.

Expression and purification of MBP-TbtE, MBP-TbtF, and MBP-TbtG. The expression and purification of these proteins are performed according to a previously reported protocol.[7]

***In vitro* transcription of *T.bispora* tRNA^{Glu} (CUC).** The protocol was done following a previous publication first describing the usage of *T.bispora* tRNA^{Glu} (CUC) in the thiopeptide thiomuracin biosynthesis.[7] Briefly, the tRNA^{Glu} dsDNA template was generated from two overlapping synthetic deoxyoligonucleotides with sequences provided in Table S3.

To make dsDNA template for *in vitro* transcription, 5′ overhangs were assembled using this reaction condition: NEB Buffer 2 (1×), primers (4 µM each), dNTP (100 µM each), DNA polymerase I large (Klenow) fragment (1 U/µg DNA) in a final volume of 50 µL. The reaction was incubated at 25 °C for 15 min, quenched with EDTA (10 mM) at 75 °C for 25 min, and dsDNA tRNA^{Glu} template was precipitated with cold EtOH overnight. The DNA template was then washed twice with 75% cold EtOH, and the supernatant was removed through centrifugation for 20 min at 13000 × g. The pellet was then air-dried for 15 min before being dissolved in H_2O. For a 50 µL PCR scale, 10 µL H_2O was used to dissolve the DNA pellet.

In vitro transcription was performed using this reaction condition: 100 mM HEPES-KOH pH 7.5, 36 mM $MgCl_2$, 50 mM DTT, 7.5 mM each rNTPs, 2 mM spermidine-HCl, 0.1 mg/mL bovine serum albumin (RNase-Free), 0.8 U/µL Ribolock RNAse Inhibitor, 0.5 mU/µL *E. coli* inorganic phosphatase, (Thermo Scientific), 100 ng/µL DNA template, and 10 U/µL T7 RNA polymerase. The reaction was incubated overnight in an air chamber at 37 °C.

The transcribed tRNA^{Glu} was then purified by acidic phenol extraction. Specifically, 0.05 U/µL of RNase-free DNase was added to the transcription mixture and incubated for 30 min at 37 °C. The reaction mixture was then buffer exchanged with 1000x volume of 100 mM HEPES pH 7.5 by Amicon filter (30 kDa) to remove residual rNTPs. The RNA was then extracted with an equal volume of acidic phenol. The phenol phase was then back extracted with an equal volume of 300 mM NaOAc pH 5.2 and combined with the aqueous phase. This extraction was repeated one more time before the aqueous phase was extracted twice with a mixture of chloroform and isoamyl alcohol (24:1). The aqueous phase (top phase) was then collected and precipitated with 2.5 times volumes of EtOH. The supernatant was removed after 13,000 × g centrifugation 15 min, and the pellet was then washed twice with 75% EtOH. The supernatant was again removed after 13,000 × g centrifugation for 10 min, and air-dried for 15 min. The pellet was then redissolved

in 2 mM NaOAc pH 5.2, and the concentration was assayed using 260 nm absorbance. This $tRNA^{Glu}$ (CUC) was then used in the MroBC-catalyzed dehydration assay.

***In vitro* translation and enzymatic assays (dehydration and cyclization).** In a 0.65 mL protein low-binding Eppendorf tube placed on ice, 0.75 µL of purified *mroA* variant template DNA was mixed with 0.75 µL of Solution B and 1 µL of Solution A of PURExpress *In vitro* Protein Synthesis Kit (E6800L) purchased from NEB (total volume of reaction is 2.5 µL). The translation reactions were performed at 37 °C for 1 h on an aluminum block.

For a full MroBCD substrate scope investigation, a total translation volume for each variant of 7.5 µL was performed. 1.5 µL of 90 mM iodoacetamide (IAA) was added to quench dithiothreitol (DTT) in the translation reaction mixture, a thiol-based nucleophile that can react with electrophilic dehydroalanines generated from MroBC-catalyzed dehydration assays. The translation product was split into two parts with these corresponding volumes: 3 µL and 6 µL. In the 6 µL part, 10.2 µL of the enzyme mix containing enzymes and cofactors was added along with 1.8 µL 90 mM ATP (pH 7.5), which was used to initiate the reaction. The enzyme mix was made by adding these components in the following order: HEPES pH 7.5, $MgCl_2$, glutamate, *T. bispora* $tRNA^{Glu}$(CUC), thermostable inorganic pyrophosphatase (TIPP), TEV (L56V/S135G/S219V) (*w/w* ratio with a total amount of MBP-tagged protein = 1: 10), MBP-MroB, MBP-MroC, and *T. bispora* GluRS. The enzyme mix was incubated 25 min at room temperature before adding to the translation product to facilitate *in situ* TEV-catalyzed cleavage of MBP from MBP-MroB and MBP-MroC. The enzymatic reaction proceeded for 1 h at room temperature. Overall, the concentration of components in the 18 µL reaction mix is as follows: 50 mM HEPES pH 7.5, 5 mM $MgCl_2$, glutamate 1 mM, 3 µM *T. bispora* $tRNA^{Glu}$, 1 µM *T. bispora* GluRS, 2 µM MBP-MroB, 2 µM MBP-MroC, 6 mM ATP, 0.027 U/µL TIPP, and 5 mM IAA. In addition, the 3 µL part was incubated with the same buffer lacking enzymes and tRNA as a translation control.

After incubation, the enzyme reaction (18 µL) was split into two equal parts. One part was treated with MBP-MroD such that the final concentration of MBP-MroD is 3 µM, and the other part was added the same volume but with buffer lacking MBP-MroD (50 mM HEPES, 300 mM NaCl, 2.5% glycerol, 0.5 mM TCEP). The reaction further proceeded for 1.5 h at room temperature. All reaction mixture was then desalted with solid-phase extraction using Ziptip with 0.6 µL C_{18} resin (EMD Millipore) and crystallized on an MTP384 steel target plate using either saturated sinapic acid or 50 mg/mL Super DHB [dissolved in 60% acetonitrile and 0.1% trifluoroacetic acid (TFA)] as the matrix. The crystallized spots were then analyzed using MALDI-TOF-MS (Bruker Ultraflex). The external standard for MALDI-TOF-MS analysis is ProteoMass Peptide and Protein MALDI-MS Calibration Kit (Sigma)

In this work, TEV was used in every variant tested. However, the dehydration and cyclization of wild-type substrate (MroA2) were also observed without TEV in the assay.

Enzymatic assays (dehydration and cyclization) with purified substrates. The reactions were performed similarly as described above, with 10 µM substrate concentration and without IAA. The total volume of each reaction is 100 µL.

Computational generation of random sequences. ExPASy RandSeq tool (https://web.expasy.org/randseq/) was utilized to generate random peptide sequences for assessing the substrate scope of large ring formation. The composition of amino acids in the peptide sequences was specified to be 5.88% for each 17 canonical amino acids (Ala, Arg, Asp, Asn, Gln, Glu, Gly, His, Ile, Leu, Lys, Met, Phe Pro, Trp, Tyr, Val) and 0% for Cys, Ser, and Thr.

C-terminal *O*-methylation using LahMet. 100 µM MBP-tagged Δ12MroA1 was incubated with 20 µM MBP-tagged $LahS_B$ in the presence of 1 mM S-adenosyl methionine (SAM) and 50 mM HEPES pH 7.5 at room temperature for 16 h.[6] The peptides generated from a 25 ml reaction were then subjected to TEV cleavage followed by solid-phase

extraction and HPLC purification, as mentioned above. Only the fractions containing the methylated peptide were collected after purification.

Dehydrothiolation of cysteines in precursor peptides to generate dehydroalanines. The MBP-tagged MroA2 precursor peptide and variants (0.1 mM) after overexpression and affinity chromatography were each incubated with TEV protease (0.01 mM) for 15 min at room temperature, followed by the addition TCEP (0.2 mM), potassium carbonate (0.1 mM), and *N, N*-dimethylformamide (DMF) (one half of the final reaction volume). Upon addition of DMF, cleaved MBP precipitated from solution. The mixture was incubated at 37 °C with agitation for 15 min. Methyl-2,5-dibromopentanoate (100 mM) was then added, and the reaction proceeded for 3 h with agitation, after which full dehydrothiolation was observed.[8] The reaction was centrifuged for 5 min at 17,000 × g, and the supernatant was subsequently collected to remove precipitated MBP from the mixture. Six times the reaction volume of diethyl ether was added, and the mixture was vortexed for 10 sec. The mixture was then centrifuged for 15 s at 6,000 × g before removing the top ether layer. This wash was repeated a second time before the tubes were incubated at 37 °C for 10 min with the cap open to remove excess diethyl ether. The dehydrothiolated substrates were then dried by a SpeedVac Vacuum concentrator (Thermo Scientific) and resuspended in 50 mM HEPES pH 7.5 prior to the [4+2] cyclization assay.

***In vitro* translation and thiazol(in)es-containing pyritide biosynthesis by TbtE/F/G and MroB/C/D.** The *in vitro* translation was performed as mentioned above in a 15 µL scale reaction. After the substrate was generated, the mixture was generated into two parts: 5 and 10 µL part. The 10 µL part was incubated with 2 µM MBP-TbtE (with no TEV cleavage site), 2 µM MBP-TbtF, 2 µM MBP-TbtG, 20 mM of $MgCl_2$, 6 mM ATP, 50 mM HEPES pH 7.5, and TEV(L56V/S135G/S219V) (*w/w* ratio with total amount of MBP-tagged protein = 1:7) for 18 h at room temperature in a 20 µL reaction. MBP-TbtF and MBP-TbtG contain a TEV cleavage site (ENLYFQS) between MBP and the protein of interest. The 5 µL part was incubated with the same mixture but did not contain any enzymes as a negative control. 2.5 out of 5 µL was incubated with 5 mM IAA for 1 h at room temperature.

The 20 µL Tbt/E/F/G reaction was then divided equally into two parts. The first half (10 µL) was incubated with 2 µM MBP-MroB, 2 µM MBP-MroC, 6 mM ATP, 50 mM HEPES pH 7.5, 1 µM of *T. bispora* GluRS, glutamate 1 mM, 3 µM *T. bispora* $tRNA^{Glu}$ (CUC), 0.027 U μL^{-1} TIPP, and 5 mM IAA for 1.5 h at room temperature in a 30 µL reaction. In the second half (10 µL), 5 µL was incubated with the same mixture but did not contain any enzymes and tRNA as a negative control. The remaining 5 µL underwent a similar incubation process but without any IAA.

The 30-µL reaction MroB/C reaction was then divided equally into two parts. One part was treated with MBP-MroD such that the final concentration of MBP-MroD is 3 µM, and the other part was added the same volume but with buffer lacking MBP-MroD (50 mM HEPES, 300 mM NaCl, 2.5% glycerol, 0.5 mM TCEP). The reaction further proceeded for 1.5 h at room temperature. All reaction mixture was then desalted with solid-phase extraction using Ziptip and analyzed with MALDI-TOF-MS as mentioned above.

For LC-HR-ESI-MS analysis, a 30 µL scale *in vitro* translation reaction was performed and incubated with Tbt/E/F/G and MroB/C/D as mentioned above but without any splitting, resulting in a 185 µL reaction after adding all enzymes and necessary components. This mixture was then desalted with solid-phase extraction using an 8 mg Pierce C18 Spin Column (Thermo-Fisher Scientific). The desalting protocol was performed following the manufacturer's instructions but omitted TFA. The eluant (in 80% acetonitrile) was centrifuged at 13,000 × g. Then, the supernatant was collected and directly injected onto LC-MS.

LC-HR-ESI-MS/MS analysis of dehydration and cyclization assays. Enzymatic assays were desalted using solid-phase extraction prior to LC-ESI-MS/MS analysis. Specifically, except for the thiazol(in)es-containing pyritide (which

utilize 8 mg Pierce C18 Spin Column), the samples were applied to Toptip C18 (10-200 μL, Glygen Corp) wetted with 50 μL of 80% acetonitrile (0.1% formic acid) and equilibrate with 150 μL of 0.1% formic acid following the manufacturer instructions. The C18 columns (tips) are then washed with 150 μL of 0.1% formic acid and eluted using 100 μL 80% acetonitrile (0.1% formic acid). The samples were then dried utilizing lyophilization and redissolved in 25% acetonitrile (80 μL for enzymatic assays of *in vitro*-translated substrates and 200 μL for enzymatic assays of purified substrates). Then 20 μL of each sample was injected into an Agilent AdvanceBio Peptide Plus column (2.1 x 150 mm, 2.7 μm) equipped with an Agilent 6545B Q-TOF interfaced with an Agilent 1290 Infinity II LC system. Mobile phase solvents were composed of H_2O, 0.1% formic acid (Solvent A), and acetonitrile 0.1% formic acid (Solvent B). The column compartment was maintained at 35 °C during all experiments. The column was equilibrated with 5 column volumes of starting mobile phase (95% A and 5% B) between injections. The gradient of all LC runs was as follows: 0-2 min: 95% A 5% B, 2-3 min: 70% A 30% B, 3-18 min: 20% A 80% B, 18-20 min: 5% A 95% B. The samples were run to waste for the first 3 min before applying to the mass spectrometer. Mass range was set from 100 to 1700 m/z (except for Δ12MroA1 W7G:100-3000 m/z). MS parameters were as follows: gas, 320 °C at 8 L/min; nebulizer, 35 psig; nozzle voltage, 1000 V; sheath gas, 350°C at 11 L/min; capillary, 3500 V; fragmentor, 125 V; skimmer, 65 V; MS scan rate (10 spectra/s); MS-MS scan rate (5 spectra/s); and isolation width (MS/MS), 1.3 *m/z*. The MS was operated in positive ionization mode for all samples analyzed, and fragmentation was performed using collision-induced dissociation (CID) at 25 eV. For the thiazol(in)es-containing pyritides, the nozzle voltage utilized was 0 V. Data analysis was conducted using Agilent MassHunter Qualitative Analysis 10.0. The exact mass lists are exported and analyzed using IPSA[9] and mMass.[10]

For Thr-containing substrates in Figure S39, *in vitro* translation reactions (12.5 μL) were performed followed by MroB/C/D assays as mentioned above and GluC (NEB) digestion (500 ng) in NEB's GluC Reaction Buffer for 12 h at room temperature. These mixtures were then desalted using solid-phase extraction with Toptip C18 as mentioned above prior to LC-MS/MS analysis. Fragmention was performed as mentioned above using CID at 22 or 25 eV. For GluC-digested dehydrated MroA1 G5T (VGADhaWLTDhaWVI), fragmention was performed using CID at 10 eV instead.

HR-ESI-MS/MS (non-LC) analysis of dehydration and cyclization assays. MroB/C and MroB/C/D assays of triArg-MroA1 and triArg-MroA2 were analyzed with high-resolution tandem-mass spectrometry without liquid chromatography on a ThermoFisher Scientific Orbitrap Fusion ESI-MS using an Advion TriVersa Nanomate 100. The assays were desalted using C_{18} Ziptip (EMD Millipore) and eluted using 80% acetonitrile with 1% acetic acid. The MS was calibrated and tuned with Pierce LTQ Velos ESI Positive Ion Calibration Solution (ThermoFisher). Samples were directly infused into a ThermoFisher Scientific Orbitrap Fusion ESI-MS using an Advion TriVersa Nanomate 100. The MS was calibrated and tuned with Pierce LTQ Velos ESI Positive Ion Calibration Solution (ThermoFisher). The MS was operated using the following parameters: mass range, 100-2000 m/z; resolution, 120,000; isolation width (MS/MS), 1 *m/z*; normalized collision energy (MS/MS), 30 (didehydrated MroA1, MroA1 and MroA2 ejected leader peptide) or 70 (didehydrated MroA2); activation q value (MS/MS), 0.4; activation time (MS/MS), 30 ms. Fragmentation was performed using collision-induced dissociation (CID) at 30% or 70%. Data analysis was conducted using the Qualbrowser application of Xcalibur software (Thermo-Fisher Scientific). The exact mass lists are exported and analyzed using IPSA[9] and mMass.[10]

Solid-Phase Peptide Synthesis (SPPS) protocol of Gly-Ala-MroA1 core peptide. Manual fluorenylmethyloxycarbonyl (Fmoc) SPPS was performed at room temperature using a 25 mL fritted glass funnel as a reaction vessel, dimethylformamide (DMF) as a solvent, 2-(6-Chloro-1-H-benzotriazole-1-yl)-1,1,3,3-tetramethylaminium hexafluorophosphate (HCTU) as an activator, 20:80 *N*-methylmorpholine: DMF as coupling solution, 20:80 piperidine: DMF as deprotection solution, and 60:40 acetic anhydride: pyridine as a capping solution. The peptide was synthesized on a 0.05 mmol scale starting from the Fmoc-Ile Wang resin. The resin was bubbled

twice with 5 mL of deprotection solution for each coupling cycle, followed by washing five times with DMF. Next, a 5-molar equivalence of Fmoc-amino acid and HCTU was dissolved in a 5-mL coupling solution and added to the resin. Coupling was performed for 15-20 min, followed by washing 5 times with DMF. After the last amino acid was coupled, the peptide was deprotected and capped with 5 mL of capping solution for 30 min. Finally, the resin was washed with DMF and dichloromethane then dried under vacuum. For global deprotection and cleavage from the linker, the resin was resuspended in 5 mL of deprotection solution (TFA: Triisopropylsilane: H_2O 95: 2.5: 2.5) for 2 h at room temperature. The solution was filtered by passing through a glass wool-packed pipet, then gently dried under nitrogen to ~1 mL final volume and added dropwise to 10 mL of ice-cold diethyl ether to precipitate the peptide. The precipitate was collected by centrifugation, dissolved in ~5 mL of DMF, and further purified by RP-HPLC (Shimadzu LC system) using the following condition:

Column	Macherey Nagel-C18 HTec, 250 x 10 mm, 5 µm
Solvent	A: 0.1% TFA in H_2O<br>B: 0.1% TFA in acetonitrile
Gradient	1 mL injection, 4 mL/min<br>0-15 min : 2-30 % B<br>15-45min: 30-60% B

Under these conditions, the MroA1 core peptide elutes around 32-34 min.

Protocol to produce fluorescein-labeled Δ12MroA1. HPLC-purified Δ12MroA1 was dissolved in 50 µL of 100 mM sodium borate pH 8.4 to 0.5 -2 mM. To this solution, 50 µL of 5/6-carboxyfluorescein succinimidyl ester (Thermo Fisher) in DMF (10 mg/mL) was slowly added. The reaction was quickly mixed and protected from light. After 2-4 h, the reaction progress was checked with MALDI-TOF MS (successful labeling was indicated by an +358 adduct). The reaction was diluted 10-fold with 100 mM Tris pH 8, then subjected to centrifugation to remove insoluble materials. The supernatant was injected onto the RP-HPLC Phenomenex Luna C5 column (250-10cm, 100 Angstrom, 5 microns) connected to an HPLC system (Shimadzu) running at 4 mL/min of solvent A (H_2O + 20 mM ammonium acetate) and solvent B (acetonitrile). The following gradient was used: 0-15 min: 2-30 % B, 15-45 min: 30-60 % B. HPLC fractions were monitored by MALDI-TOF MS (Bruker Ultraflex). Labeled peptide elutes around 22-25 min. These fractions were collected, protected from light, and lyophilized to dryness.

Fluorescence polarization to measure K_D of MroA1 with MroB or MroD. All proteins and peptides were prepared in the 0.5x storage buffer before concentration/FP measurement. Experiments were done in triplicates. Stock fluorescein-labeled peptide was measured concentration using A_{490} (ε: 70,000 $M^{-1}cm^{-1}$). Initial sample was prepared: 5 nM labelled Δ12MroA1 (for MroD; for MroB, only 2 nM labelled Δ12MroA1 was used), 10 µM MBP-MroB (A_{280} ε: 178,885 $M^{-1}cm^{-1}$) or 10uM MBP-MroD (A_{280} ε: 115,740 $M^{-1}cm^{-1}$). In a black 96-well plate (Corning 3686), 50 µL of initial sample was added to the first well, followed by 11 3-fold dilutions into subsequent wells containing 5 nM labeled Δ12MroA1. The plate was covered from light and incubated at room temperature for 1 h, and then fluorescence polarization was measured (Biotek Synergy H4 hybrid reader) using the following filter (Excitation: Emission – 485 nm / 20 nm: 518 nm / 20 nm). The obtained data was converted to anisotropy value and plotted against protein concentration. Using the OriginPro software, the data were fitted to receptor depletion equation:[11]

$$y = A1 + (A2 - A1)\frac{(Lt + Kd + x) - \sqrt{(Lt + Kd + x)^2 - 4 * Lt * x}}{2Lt}$$

Where: y = anisotropy value, A1 = minimum anisotropy, A2 = maximum anisotropy, Lt = probe concentration, and x = total enzyme concentration.

Competition fluorescence polarization. The initial sample contained 80 nM enzyme, 5 nM labeled Δ12MroA1 peptide, and competitor peptide (concentration is from 20 µM to 100 µM, depending on the experiment). In a black 384-well plate (Corning 3575), 30 uL of initial sample was added to the first well, followed by 13 2-fold dilutions into subsequent wells containing 80 nM MBP-MroB and 5nM labeled Δ12MroA1. The plate was covered from light and incubated at room temperature for 10 min, and then fluorescence polarization was measured (Biotek Synergy H4 hybrid reader) using the following filter (Ex: Em – 485 nm / 20 nm: 518 nm / 20 nm). The obtained data was converted to anisotropy value and plotted against peptide concentration, and fitted to dose-response function:

$$y = A1 + \frac{(A2 - A1)}{1 + (\frac{IC50}{x})^p}$$

Where: y = anisotropy value, A1 = minimum anisotropy, A2 = maximum anisotropy, p = Hill's coefficient, x = competitor concentration. The obtained IC_{50} was used to calculate inhibitor constant K_I using the following equation:[12]

$$Ki = \frac{IC50}{1 + \frac{Lt(y+2)}{2 * Kd(y+1)}} - Kd(\frac{y}{y+2})$$

Where: Lt is labeled peptide concentration, y = initial bound/free ratio of the labeled peptide before adding competitor, and Kd is the binding constant.

In cases where competitor concentration was not high enough to achieve a plateau, the competition curve was fitted with minimum anisotropy fixed to be the same value as the anisotropy value of the free probe solution. This calculation assumed that the labeled peptide was completely displaced from the enzyme binding pocket at a very high concentration of the competitor. Hence, the anisotropy value was similar to the sample with only the labeled peptide. The specific minimum anisotropy used in this experiment for both MroB and MroD was 65.

Table S1: Sequence of *Mro* biosynthetic genes and *T. bispora* GluRS for optimal *E. coli* expression. All sequences are provided 5′ to 3′. Restriction sites for cloning are underlined (5′ BamHI, 3′ XhoI). These gene constructs were synthesized by GenScript (Piscataway, NJ, USA).

mroA1 (NCBI accession of encoded peptide: WP_157527607.1)
GGATCCATGGACAACGTCGTGACTGAAGCCGCCGAGTTCGCGGATTTGGACATTGTTGATCTGGACCTTGCAGTAGATGAAGAACT
TGCCGCTTTGAGCGTCGGGGGTTTGGGTAATACAGAGGTCGGTGCGaGCGGTTGGCTGGGCaGCTGGGTGATTTGACTCGAG

mroA2 (NCBI accession of encoded peptide: WP_157527609.1)
GGATCCATGGACAATGCGGCTACGGAAGCTACGGAGTTTGCAGACCTTGATATTGTGAACCTGGACCTCCCTATCGACGAAGAAC
TTGCCGCTGTCAGCGTCGGGGGTTTGGGTAATACAGAGGTCGGTGCGTCCGGATTTTTTGGTCGTTCCTGGTTAATCTGACTCGA
G

mroB (NCBI accession of encoded protein: WP_067368389.1)
GGATCCATGCCGGCACCGCCCACGGCAGCGACGGGCCCAGTTGTCGTGGTGCGTGTCGCGGCATTGCCGACGCAGGCCTTAGATG
ATACTGCGGCGCCCGCCAGCTGGGCAATGGTACAGGCTATTCTGGCGTCCCGCCGTCGTGTAGCGGAAGCCGGCGCCCGTCTTGC
GGATGAAGTTCACGGTTTCATTGCCGATCCGGCGCTTGCGGGTGCCCGTCCTGAACTGGTTGCTTTACGCCGTGCCTTACATAAT
CGTCGCCGTCCTGGCCATCGCGCATGGCCTGGCGGCCATGCTGAATTACTTCCGGCCCGGTTTCGCAGTGAGTTAACCGGTTGGA
CGGCTGAGTTGGACCGTGCGGCCGCGCTGACCCGTCAGCTGCCAGATCTGCTGGATGCTGAGTGGACACGCTCTTTGCGCGCGCT
GCGCCGTTGGAGTGCGACCGAAGTTTTCGAATTTGGCTTATTGCAGTCATCTGAAGACCTGTTGCATGCACTTCTGCGTTGGCGT
GATCAGCCGGAAGGAACCCCACCGCGTGCTCAGGTCGCACTGCGTCTGGCGAAATATCTGGCGCGCGCAGTGGCAAAGACCTCTC
CGCAAGCCACCTTTATGATGTCTGGCCTGTGCCGCTGGAGTGATGCACCGACCGCGGTGCAGCCTACAGGTCGCTGGGCGTGGCG
CTCCGTAGTCGAGCCGAACGTCCCGCTCCTGCAGGCGCTGACTCGCCGTCTGCCAGCTGATTCAACGGCCACCGGTCGCCTGATG
GTGGTAACCACGCCGGACGCGGACGTGGCTGATGGTCGGATCTGGTTTGCTCATCCGCGTCGTGAGCGCATTCAGGGTCTGTTAC
TCACGCCTCCGGTGCGCACTGCGATCGAAGCAGTTGGCGGCGGGGCCCCACTGGCTGAAGTAGGACGCCGCCTTGCGGGCGATAC
GGCCGTCACGTTCGATCGTCTCGTCGAGGTCGGCTTATTCGAAATCCAGCCACCTTTTGCCGATCAAGCAGCGGATCACCTTCAC
GACTTACGCGCATGGCTGGGGCAGGAAGCACCTGCGGCCGGACCGCTGCGTGCGTTAGCGGACGCCCTGGATGCCTACCCTGCAC
CAGGTACCGATGCGTCTCGCCGTCGTGAGCTCCACCGCCGTATTCGCACAGAATTTCAGGCCGTCCACGGGCCTGTTGGCGATAC
GCCGCTGCCTGCGCGCAACCTGTTTCGCGAAAACGCCGTATTCACCGCTGAACCCGTGCGCCTGGGCGGGGTGCCGTGGCGGCCG
GCGGTCGCTGATTTGCACGTAGTTCATCGTTTACTCGCGCTGTTTCAACATTCTACCGTAGTCCGCCTGGTGGCACATCGTGTTG
TCGTGGATCGCTATGGTGTAGATACGCGTGTTGGGTTACTCGAACTGCATCGGGAACTGAGCCGTCTGGCGGTTGATGCGGAGCC
CGGCACAGCCGTGGCAGAATTGGCCGGCCTGTTACTGCTGCGTCCGGGGCAGCCTCCGGACCCAGCTCGCAGCACCCTTCCGGAG
GTCCGTTGGCTGCACCGCTTACGTCAACAAGCATTGGCTGCTCTTTGGGCTGGGCCTGAAACGCCGCCGGAGWGGCGTGACGGTG
GATCCGGCTCGCGTGCGTGCTCTGGCCGATGGCTGGCCGCCATGGGTCCGTCCGGCGCCGGCGCTTGCATGCTTTGTGCAGCAGG
TTCCGGCAGCGGACGGCTTGCGCCTGGTATTGAACGGCGTGGGTGCGGGACACGGCGCTGTTCGTTCACGCGTCCATCGCTTACT
GGCAAAGGTGGCGCCAGAAGGTGCCGGCACCGGAGAGCTGCCTAAAGCCACGCCGACCCCTGCGCCGGCCGGCTACCTGGCGGAG
ATTACCGGCTTGTTTGGTTCGACCGTGAACCTGCGTAGCCCGGCTGTGGATTTTGAAATTTCGCATCCCGGTACTGTATCTGCAC
GCCCTCCGCAGCATCGTATCGCCCTCGACGATCTGGTTGTTACTGCGGATGGTACGACCCGTAGTCTTCGCCTGCGTCGCCGTTC
AGATGATGGCGAGGTACGCATTGCGCATACAGGTATGATGGCAGGCGCTCGGTTGCCGCGCTTGGCGCGGTTCCTGATTCACTTG
TTTGGCGATGTCCCGAACGCCGCCGCGCCTACGGCGCAGATCTTTGCCGGTACCCCGGAGCACCAGCCGGGCGCAGTGCGGCGCC
AACCGCGCCTGTCTATTGGCCGTGTAACTGTAAGTCGCGCTTCATGGCGCGTCCGTGCCGACCTCGTTCCGCGTCGTGCTAAAGG
CGTCGATGATGCGGGTTACCTGTTGGCCCTGGCTGCATGGTTCGCCGATCACCGCATCCCGCCACGCACCTATGTAACCGCAACC
GGCCCGACGCCGCCCGGTACGCCTCCGGTGCCAGTAAAACCGATCTATTTGGACCTGGCCAATCTGCTGCTGGTGCGCCTGTTTG
AACGGCTGCTGCGTGAACCGGATAGCGTGCTGGTATTCACTGAAGCGCTGCCTCAGCTGGCTGATGCTCCATGTTTTGGAACGGA
AGGACGCCATGTAACAGAATACGTGGTAGAGATTGGACTGCCATGACTCGAG

mroC (NCBI accession of encoded protein: WP_083978639.1)
GGATCCATGTTACGTCACCGCGGTCAAGCGCGCGATCGTGTTCGTCGTCGCGATCGTCCGCCTGTAACACCACCGCCCACTTGGCT
TAGTTGGCACGCGTTCCACCACGGTGACCATACGCGCCTGATTACCGGTGCAGTAGACCCGCTCGTTGCGAGTCTGCAGGCAGACG
GCCGTATTGCACAGTTCTTCTTTATCCGTTACTGGGAAGGCGGTCCTCACCTGCGCTTACGTCTGTTGCCCACTCACGCGGAGTAC
TCAACTGAAGTGGCGGCACGCGCTCGTTCGGCCTTAGAGCGTCATCTTGCGCGCTTTCCGTCGCCTCCCTCCGCGCCTGCGGGCCA
ATATGCCGCTTTGGCTGAGCGCTACGCGCGCTTAGAAGGGTTAGCCGATCATGACCGCCGCCTGCGCCCGCCGGATGTAGTTGAGG

CAGTCCCTTACCACCCAGAGTACCCCGTGTTTGGCGGCCGCGCCGCCACTCGTGCCGTAGAGCGTCACTTCACGGACTCTAGCCGC
CTGGCGTTGAGCGTCCTTGCGCGTCGCGCGCCCCAACACCGTAGCCGCCTCGCAGCGCTGGCCCTTGCCTCGACCCTTGCGGCGTG
GCAGCCAGACCGTCGCCGCTTAGCGCACCTGTTGACTCGTTCCCGCTCATTTTGGGAGCCAGCCGAGGGCCGCGGTCTGCGTCGCG
AGAGCTACCAACGTCAACGCGCAGCCCTGCGTCGTCTCGTTTCGGGCTGTTGGCCGCCGACCCCTGGCCCACCACCCCAAGATCCC
GATCCGTTGGCCGCTGCCTGGTCTGATAGCGTCCACCGTCTTCACGCGGAGCTGACAGACCTGCGTCGTGCCGGGGATTTCCATCC
TGACTTGCGTGCGGTGGCTACGCGCTTTGCCGCCGATACCGGTCCCACGCGCGACGAGCAAGGTTTACTGGTTGTGCTTCTCCGCT
GCGTGCACCTTCTGTGTAATCGCTTAGGTCTTGGTGTGGACCAGGAGACCCAATTACGTTACTTAGTGTGTGCCGCCTGTGCGGAC
ATCGAGGCCGTGCCAGAGCAAGAGACACCATAACTCGAG

mroD (NCBI accession of encoded protein: WP_067368384.1)
GGATCCATGACACGTACCCCGCCGCATCGCGAAGAACCGGGTTGGCATAGTATCCAAATTCGGTATCATGCTGAGAACAAAGACGA
TTTGATTCTGGATGCGGTTCACCCATTACTTACGGCCCTGGCCGACGCGGTTGACCAACCTCACCTGTTACGCCACTGGCGCCGGG
GCCCGCACCTCCGCATCAACCTGCGTACCACCCCGCAGCTCTGGGCAGAAGTTGTTCGCCCGACCGCCCAGCGCGATCTTACCGCG
TACCTGCATGATAACCCATCAACCGCCAGCTTACGCGAAGCCGAACATCTGGCGGCCCACCGCCGCCTGGCAGTGCGTGAGGCGGA
TCATGGACCTCTGACTCCCTGGTACCCTGATAATACAGTTCAATTTGAACCGCATGAAGATCGGCAGCATGTTCTGGGTAGCCCGG
CGCTTGCAGGTTTGCTGGCGGATGGCTATGCACGCTCGACTGCCTTAACCGTGGATACGCTGGCTGGCGTTCGTTCTGGAGCGGTG
GATCGCGTAGGGCATGCTCTTGATTTAATGTTTGCATTTGGTCATCTGTCTGTGCCGCCGATTTCTCGCGGCTACATGTCCTTCCG
CTCACATGTGGAAAGTTTTTTGGGTTATACCGCTGATCCGGATGCCGTCCGCTCGACCTTCGATGAGCGTTACCATCGGCACCGGC
CCGCGCTGCGCGACCGTTTACATACTACGCGCGCCATTGTTGCGGGAAGTCGCCGTGATCCGCTGGTGGAGGCATGGCTGGAAATC
GTGCGTCGCCAGAAAGCTCTGGCGGAACCGCTGTTTACGTCTGGCGCCATTGATCTGGACCATCTTGATCAGCGCGGGATGGAATT
ACGCCACCGCATCGAGTTTCACGATATTCTGCGCGAGACCACTGCACATCGTCGTGAGGTACTTTCACAGGTGTGGTTTCGCTGTC
ATCGCTTAGCCATTAACCAACTGTACAGCCATTTGTCTCGCATCGGTATCGTGCCCACTCAGCGCTACTTACTCTGCCATCTGGTA
GCTCGCACCGTGGAGGAAGAATATGATATCAGTCCGGTGGCGCTCGCCCGTCAGTTTGTCAACGACCGTCGCTGACTCGAG

T. bispora **GluRS** (NCBI accession of encoded protein: ADG89504.1)
ATGATTCGTGTGCGTTTCGCACCGTCTCCGACCGGCATGTTCCACGTCGGTGGTGCCCGCTCTGCCCTGTACAACTGGGCCTTCG
CCCTGCGTCACGGCGGCACCTTCGTCCTGCGCATCGAGGACACCGACCAGTCTCGCAACCGTCCGGAGTGGACCGAGGGCATCAT
CTCTGCCCTGGCCTGGCTGGGCATCAGCAAGGAGTCTCCGCACTTCGAGGGTCCGTACTTCCAGTCCTCTTACGAGAAGCTGCAC
CGCGAGGCGGCCCAGCGTCTGCTGTCTGAGGGTAAGGCATACTACTGCACCTGCACCCGTGACGACGTGAAGGCCCGTACCGGCT
CCGAGTACCAGGGTTACGACGGTTACTGCCGTGATCGTGGCCTGACCGAGGGTGCGATCCGTTTCCGCACCCCGGACGACGGTGT
TACCGTTGTGGACGACGTGGTCCGTGGCCGTGTGGAGTTCCCGAACGCGGCGATGGAGGACTTCGTCATCGTGCGCGGTGACGGC
TCTCCGCTGTTCATCCTGGCCAACGTTGTGGACGACATGGAGATGCGTATCACCCACGTGATCCGTGCCGAAGAGCATCTGAGCA
ACACTCCGAAGCAGCAACTGCTGTGGGAAGCTCTGGGTGCTCGTCCGCCGGTGTGGGCTCACGTCCCGGTGATCGTCAACGAGAA
GCGTCAGAAGCTGTCCAAGCGTCGTGACAAGGTCGCGCTGGAGTCCTACCGCGAGGAGGGCTACCTGCCGGAGGCGATGGTCAAC
TACCTGATGCTGCTGGGCTGGGGTCCGGGTGGCGATCGTGAGATCATGCCGTGGCCGGAGATGGTGCAGTGCTTCCGTCTGGAGG
ACGTCAACCCGTCTCCGGCGTTCTTCGACGAGAAGAAGCTGCGCGCGTTCAACGGTGAGTACATCCGTGCCCTGTCTCCGGAGGA
CTTCGCCGCCCGTTGCGAGCCGTACCTGGATCCGTCTTGGGATCGTAAGGTGTTCGCACGCGTCGCTCCGCTGGCACAGACCCGT
ATCTCCGTGCTGTCTGAGATCCGTGACTACGTAGACTTCCTGTTCCTGGACGAACCGGTTTTCGATCAGGCATCCTGGGACAAGG
CTATGAAGCCGGGTGCAGCCGAGATCCTGGCCGAGTACGCTGAGCGTCTGGAGTCTGTGGAGTGGACCCCGGAAGCGCTGAAGAC
TGCGCTGGAGGAGGTGGGTGCAGCTCACGGTCTCAAACTGGCTAAAGCTCAGGCTCCGGTTCGTGTGGCTGTAACCGGTCGTACT
GTCGGTCTGCCGCTGTTCGAATCTATCGAGGTGCTGGGTCGTGAGCGCTCTCTGGCACGTGTGCGCGCTGCGCTGGCCAAGCTGT
CCGCTTAA

Table S2: Nucleotide sequence of open reading frames inserted into plasmids used in this study.

a) Maltose-binding protein in **pET28-MBP**

```
ATGGGCAGCAGCCATCATCATCATCATCACAGCAGCGGCCTGGTGCCGCGCGGCAGCCATATGAAAATCGAAGAAGGTAAACTGG
TAATCTGGATTAACGGCGATAAAGGCTATAACGGTCTCGCTGAAGTCGGTAAGAAATTCGAGAAAGATACCGGAATTAAAGTCAC
CGTTGAGCATCCGGATAAACTGGAAGAGAAATTCCCACAGGTTGCGGCAACTGGCGATGGCCCTGACATTATCTTCTGGGCACAC
GACCGCTTTGGTGGCTACGCTCAATCTGGCCTGTTGGCTGAAATCACCCCGGACAAAGCGTTCCAGGACAAGCTGTATCCGTTTA
CCTGGGATGCCGTACGTTACAACGGCAAGCTGATTGCTTACCCGATCGCTGTTGAAGCGTTATCGCTGATTTATAACAAAGATCT
GCTGCCGAACCCGCCAAAAACCTGGGAAGAGATCCCGGCGCTGGATAAAGAACTGAAAGCGAAAGGTAAGAGCGCGCTGATGTTC
AACCTGCAAGAACCGTACTTCACCTGGCCGCTGATTGCTGCTGACGGGGGTTATGCGTTCAAGTATGAAAACGGCAAGTACGACA
TTAAAGACGTGGGCGTGGATAACGCTGGCGCGAAAGCGGGTCTGACCTTCCTGGTTGACCTGATTAAAAACAAACACATGAATGC
AGACACCGATTACTCCATCGCAGAAGCTGCCTTTAATAAAGGCGAAACAGCGATGACCATCAACGGCCCGTGGGCATGGTCCAAC
ATCGACACCAGCAAAGTGAATTATGGTGTAACGGTACTGCCGACCTTCAAGGGTCAACCATCCAAACCGTTCGTTGGCGTGCTGA
GCGCAGGTATTAACGCCGCCAGTCCGAACAAAGAGCTGGCGAAAGAGTTCCTCGAAAACTATCTGCTGACTGATGAAGGTCTGGA
AGCGGTTAATAAAGACAAACCGCTGGGTGCCGTAGCGCTGAAGTCTTACGAGGAAGAGTTGGCGAAAGATCCACGTATTGCCGCC
ACCATGGAAAACGCCCAGAAAGGTGAAATCATGCCGAACATCCCGCAGATGTCCGCTTTCTGGTATGCCGTGCGTACTGCGGTGA
TCAACGCCGCCAGCGGTCGTCAGACTGTCGATGAAGCCCTGAAAGACGCGCAGACTAATTCGAGCTCCCACCATCACCATCACCA
CGCGAATTCGGTACCGCTGGTTCCGCGTGGATCTGAGAACCTGTACTTCCAATCC-Insert
```

(Insert represents the nucleotides of desired precursor peptide or protein)

b) *T. bispora* GluRS – 3copytRNA^{Glu} (in the pTrc33 helper plasmid used for MBP-MroB expression)

```
ATGATTCGTGTGCGTTTCGCACCGTCTCCGACCGGCATGTTCCACGTCGGTGGTGCCCGCTCTGCCCTGTACAACTGGGCCTTCG
CCCTGCGTCACGGCGGCACCTTCGTCCTGCGCATCGAGGACACCGACCAGTCTCGCAACCGTCCGGAGTGGACCGAGGGCATCAT
CTCTGCCCTGGCCTGGCTGGGCATCAGCAAGGAGTCTCCGCACTTCGAGGGTCCGTACTTCCAGTCCTCTTACGAGAAGCTGCAC
CGCGAGGCGGCCCAGCGTCTGCTGTCTGAGGGTAAGGCATACTACTGCACCTGCACCCGTGACGACGTGAAGGCCCGTACCGGCT
CCGAGTACCAGGGTTACGACGGTTACTGCCGTGATCGTGGCCTGACCGAGGGTGCGATCCGTTTCCGCACCCCGGACGACGGTGT
TACCGTTGTGGACGACGTGGTCCGTGGCCGTGTGGAGTTCCCGAACGCGGCGATGGAGGACTTCGTCATCGTGCGCGGTGACGGC
TCTCCGCTGTTCATCCTGGCCAACGTTGTGGACGACATGGAGATGCGTATCACCCACGTGATCCGTGCCGAAGAGCATCTGAGCA
ACACTCCGAAGCAGCAACTGCTGTGGGAAGCTCTGGGTGCTCGTCCGCCGGTGTGGGCTCACGTCCCGGTGATCGTCAACGAGAA
GCGTCAGAAGCTGTCCAAGCGTCGTGACAAGGTCGCGCTGGAGTCCTACCGCGAGGAGGGCTACCTGCCGGAGGCGATGGTCAAC
TACCTGATGCTGCTGGGCTGGGGTCCGGGTGGCGATCGTGAGATCATGCCGTGGCCGGAGATGGTGCAGTGCTTCCGTCTGGAGG
ACGTCAACCCGTCTCCGGCGTTCTTCGACGAGAAGAAGCTGCGCGCGTTCAACGGTGAGTACATCCGTGCCCTGTCTCCGGAGGA
CTTCGCCGCCCGTTGCGAGCCGTACCTGGATCCGTCTTGGGATCGTAAGGTGTTCGCACGCGTCGCTCCGCTGGCACAGACCCGT
ATCTCCGTGCTGTCTGAGATCCGTGACTACGTAGACTTCCTGTTCCTGGACGAACCGGTTTTCGATCAGGCATCCTGGGACAAGG
CTATGAAGCCGGGTGCAGCCGAGATCCTGGCCGAGTACGCTGAGCGTCTGGAGTCTGTGGAGTGGACCCCGGAAGCGCTGAAGAC
TGCGCTGGAGGAGGTGGGTGCAGCTCACGGTCTCAAACTGGCTAAAGCTCAGGCTCCGGTTCGTGTGGCTGTAACCGGTCGTACT
GTCGGTCTGCCGCTGTTCGAATCTATCGAGGTGCTGGGTCGTGAGCGCTCTCTGGCACGTGTGCGCGCTGCGCTGGCCAAGCTGT
CCGCTTAAACTACTTTATGTAGTCTCCGCCGTGTAGCAAGAAATTGAGAAGTTAATACGACTCACTATAGGTCCCGTCGTCTAGA
GGCCTAGGACGCCGCCCTCTCAAGGCGGTAACGGCGGTTCGAATCCGCTCGGGACTACCAATCCTTAGCGAAAGCTAAGGATTTT
TTTTAAATTTGCACGGCAAATTTGAATTCGAGGTTTTAACTACATGTTATTAATACGACTCACTATAGGTCCCGTCGTCTAGAGG
CCTAGGACGCCGCCCTCTCAAGGCGGTAACGGCGGTTCGAATCCGCTCGGGACTACCAATCCTTAGCGAAAGCTAAGGATTTTTT
TTAAATTCTGAATGTATCGAATATGTTCGAAGCTTAAATTCAAAACCAATTTGTTAATACGACTCACTATAGGTCCCGTCGTCTA
GAGGCCTAGGACGCCGCCCTCTCAAGGCGGTAACGGCGGTTCGAATCCGCTCGGGACTACCAATCCTTAGCGAAAGCTAAGGATT
TTTTTTA
```

Table S3: Oligonucleotide primers used in plasmid constructions for heterologous expression in *E. coli*. All sequences are provided 5′ to 3′ (left to right). F indicates a forward primer, while R indicates the reverse primer. Lowercase m indicates 2′ O-methylation of the following residue.

Primer Name	Oligonucleotide Sequence
mbp-F (sequencing)	GAGGAAGAGTTGGCGAAAGATCCACGTA
T7-F (sequencing)	TAATACGACTCACTATAGGG
T7-R (sequencing)	GCTAGTTATTGCTCAGCGG
mroB internal sequencing 1	GAGCGCATTCAGGGTCTGTT
mroB internal sequencing 2	TGCTTTGTGCAGCAGGTTCC
mroC internal sequencing	GTGCTGGTATTCACTGAAGC
mroD internal sequencing	TGTTGACTCGTTCCCGCTCA
mroA1 Gibson-F	CTTCCAATCCCGTCGTCGTGGATCCATGGACAACGTCGTGACTGAAGCCGCCG
mroA1 Gibson-R	CAGTGGTGGTGGTGGTGGTGCTCGAGTCAAATCACCCAGCtGCCCAGCCAACCG
mroA2 BamHI-F	AAGGATCCATGGACAATGCGGCTACGG
mroA2 XhoI-R	AACTCGAGTCAGATTAACCAGGAACGACCAAAAAATC
mroB Gibson F-Backbone	CAGAATACGTGGTAGAGATTGGACTGCCATGACTCGAGTCGACAAGCTTGCGG
mroB Gibson R-Backbone	GTGGGCGGTGCCGGCATGGATCCGGATTGGAAGTACAGGTTC
mroB Gibson F-Insert	GAACCTGTACTTCCAATCCGGATCCATGCCGGCACCGCCCAC
mroB Gibson R-Insert	CCGCAAGCTTGTCGACTCGAGTCATGGCAGTCCAATCTCTACCACGTATTCTG
mroC BamHI-F	AAGGATCCATGTTACGTCACCGCGGTCAAG
mroC XhoI-R	AACTCGAGTTATGGTGTCTCTTGCTCTGGCAC
mroD BamHI-F	AAGGATCCATGACACGTACCCCGCCG
mroD XhoI-R	AACTCGAGTCAGCGACGGTCGTTGACAAAC
Δ12mroA1 Gibson-F	GGATCTGAGAACCTGTACTTCCAATCCGATTTGGACATTGTTGATCTGGACCT
Δ12mroA1 Gibson-R	CAGTGGTGGTGGTGGTGGTGCTCGAGTCAAATCACCCAGCTGCCCAGCCAACCG
Δ12mroA1 W7G Gibson-F	GGATCTGAGAACCTGTACTTCCAATCCGATTTGGACATTGTTGATCTGGACCT
Δ12mroA1 W7G Gibson-R	CAGTGGTGGTGGTGGTGGTGCTCGAGTCAAATCACGCCGCTGCCCAGCCAACC
Δ12mroA1 V8G Gibson-F	GGATCTGAGAACCTGTACTTCCAATCCGATTTGGACATTGTTGATCTGGACCT
Δ12mroA1 V8G Gibson-R	CAGTGGTGGTGGTGGTGGTGCTCGAGTCAAATGCCCCAGCTGCCCAGCC
Δ12mroA1 I9G Gibson-F	GGATCTGAGAACCTGTACTTCCAATCCGATTTGGACATTGTTGATCTGGACCT
Δ12mroA1 I9G Gibson-R	GCTGGGCAGCTGGGTGGGCTGACTCGAGCACCACCACCACCACCACTG
mroA2 W8G (S1C/S7C)-F	GATTTTTTGGTCGTTGTGGGTTAATCTGACTCGAGCACCACCACCAC
mroA2 W8G (S1C/S7C)-R	GAGTCAGATTAACCCACAACGACCAAAAAATCCGCACGCACC
mroA2 I10G (S1C/S7C)-F	GTTGTTGGTTAGGCTGACTCGAGCACCACCACCACCACC
mroA2 I10G (S1C/S7C)-R	GCTCGAGTCAGCCTAACCAACAACGACCAAAAAATCCGCAC
T. bispora tRNA^{Glu}(CUC)-F	AATTCCTGCAGTAATACGACTCACTATAGGTCCCGTCGTCTAGAGGCCTAGGACGC
T. bispora tRNA^{Glu}(CUC)-R	mUmGGTAGTCCCGAGCGGATTCGAACCGCCGTTACCGCCTTGAGAGGGCGGCGTCCTAGG

Figure S1: SDS-PAGE analysis of proteins used in this study. Enrichment occurs due to the use of Ni-NTA resin for affinity purification, followed by size exclusion chromatography. MBP-MroB was only successfully purified when coexpressed with *T. bispora* GluRS and tRNA^{Glu}(CUC), as demonstrated in the two rightmost lanes. The band between 50 kDa and 37 kDa found in the rightmost lane (MBP-MroB) probably resulted from cleavage occurring in the linker region between MBP and MroB. Precision Plus Protein All Blue Prestained Protein Standard was used.

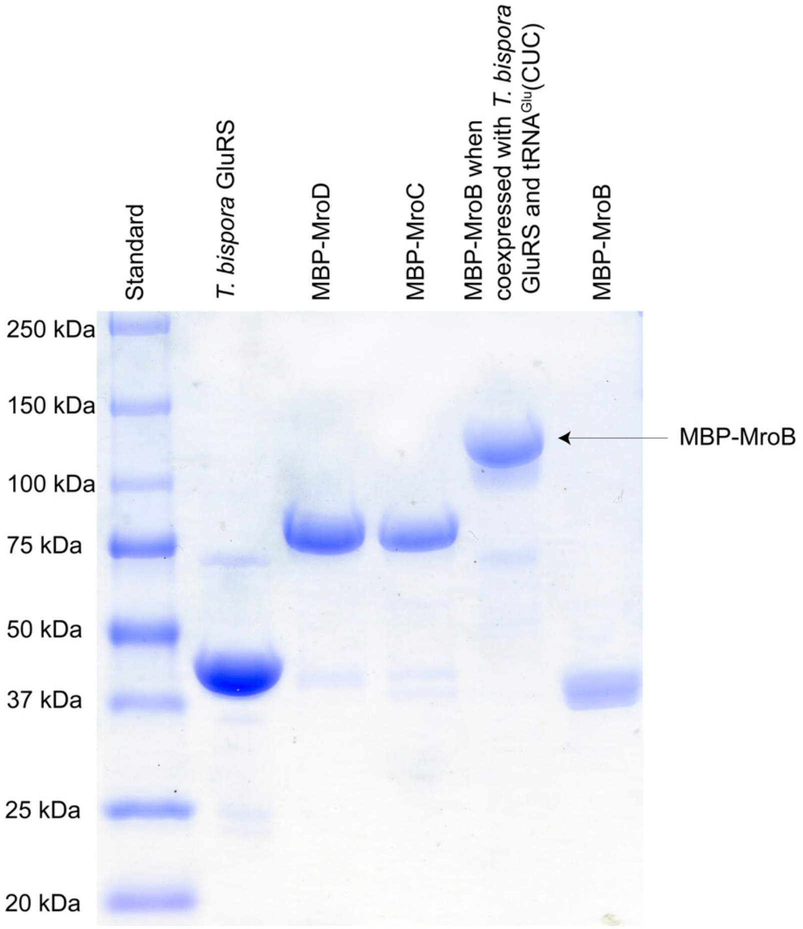

Table S4: Sequence alignment of *M. rosaria* tRNA^{Glu}(CUC) and *T. bispora* tRNA^{Glu}(CUC). The sequence identity is 91%.

M.rosaria tRNA^{Glu}(CUC) GGCCCCGTCGTCTAGCGGCCCAGGACGCCGCCCTCTCAAGGCGGTAGCGCCGGTTCGAAT
T.bispora tRNA^{Glu}(CUC) GGTCCCGTCGTCTAGAGGCCTAGGACGCCGCCCTCTCAAGGCGGTAACGGCGGTTCGAAT
                        ** ************ **** ************************* ** **********

M.rosaria tRNA^{Glu}(CUC) CCGGTCGGGGCTACCA
T.bispora tRNA^{Glu}(CUC) CCGCTCGGGACTACCA
                        *** ***** ******

Figure S2: Dehydration (MroB/C) and cyclization (MroD) of the MroA1 precursor peptide. All spectra were acquired using reflector positive mode MALDI-TOF-MS. Unless otherwise stated, all indicated peaks are $[M+H]^+$. The top MALDI-TOF mass spectrum shows unmodified triArg-MroA1 **(1a)**. The second and third spectrum **(2a)** show **1a** treated with MroB and MroB/C, respectively, in the presence of other necessary components. The fourth spectrum (**2a** + MroD) shows **1a** treated with MroB/C and MroD. The last four spectra are different mass regions of **2a** and **2a** + MroD. The # mark in the mass spectrum represents a -17 Da artifact resulting from deamination specific to reflector positive mode in MALDI-TOF-MS.[13, 14] The x mark in the mass spectrum denotes $[M+Na]^+$ ions and the xx mark denotes $[M+K]^+$ ions. The asterisk (*) in the mass spectrum represents a +16 Da species caused by air oxidation during sample preparation.[15] Ser residues bolded in purple undergo dehydration by MroB/C followed by macrocyclization by MroD.

triArg-MroA1 **(1a):** SRRRGSMDNVVTEAAEFADLDIVDLDLAVDEELAALSVGGLGNTEVGA | **S**GWLG**S**WVI

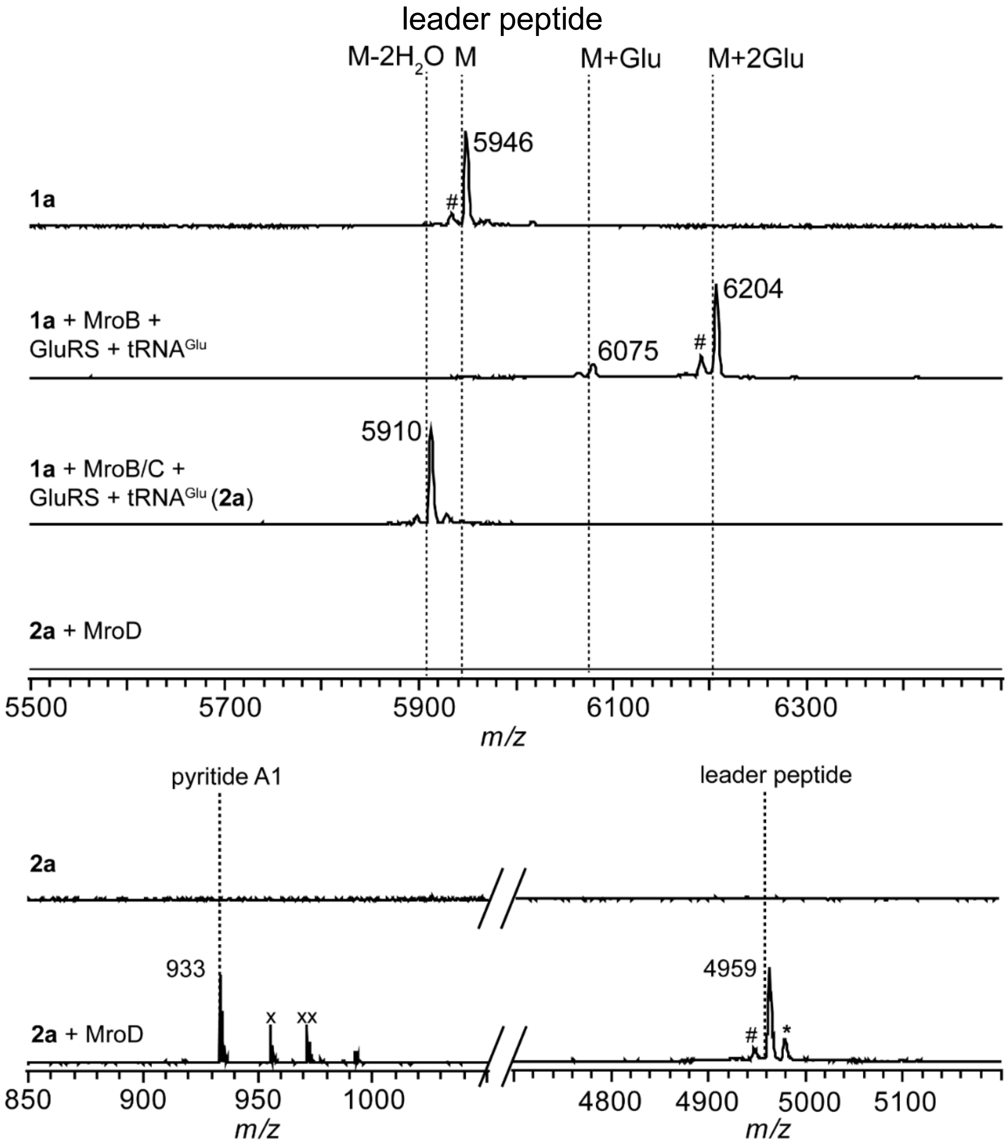

Figure S3: Dehydration (MroB/C) and cyclization (MroD) of the MroA2 precursor peptide. All spectra were acquired using reflector positive mode MALDI-TOF-MS. Unless otherwise stated, all indicated peaks are $[M+H]^+$. The top MALDI-TOF mass spectrum shows unmodified triArg-MroA2 **(1b)**. The second and third spectrum **(2b)** show **1b** treated with MroB and MroB/C, respectively, in the presence of other necessary components. The fourth spectrum (**2b** + MroD) shows **1b** treated with MroB/C and MroD. The last four spectra are different mass regions of **2b** and **2b** + MroD. The # mark in the mass spectrum represents a -17 Da MALDI artifact, resulting from deamination specific to reflector positive mode in MALDI-TOF-MS.[13, 14] Ser residues bolded purple undergo dehydration by MroB/C followed by macrocyclization by MroD.

triArg-MroA2 **(1b)**: SRRRGSMDNAATEATEFADLDIVNLDLPIDEELAAVSVGGLGNTEVGA|**S**GFFGR**S**WLI

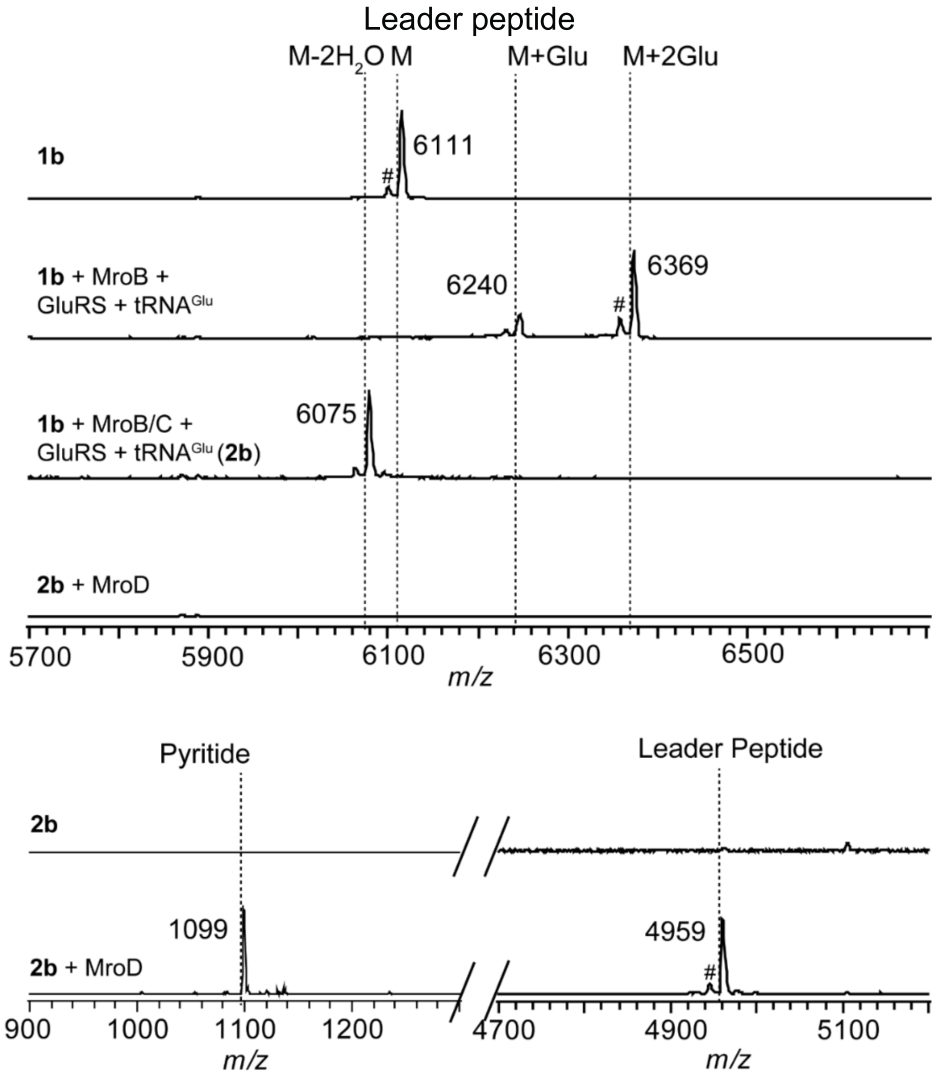

Figure S4: HR-ESI-MS/MS analysis of didehydrated MroA1. (A) HR-ESI-MS of didehydrated triArg-MroA1 formed after treatment of triArg-MroA1 with MroB/C. (B) Schematic representation of the bond cleavage of didehydrated triArg-MroA1 after CID. (C) HR-MS/MS of didehydrated triArg-MroA1 peptide after CID analysis. A table comparing observed and theoretical *m/z* values for fragments may be found in **Supplementary Dataset 2**.

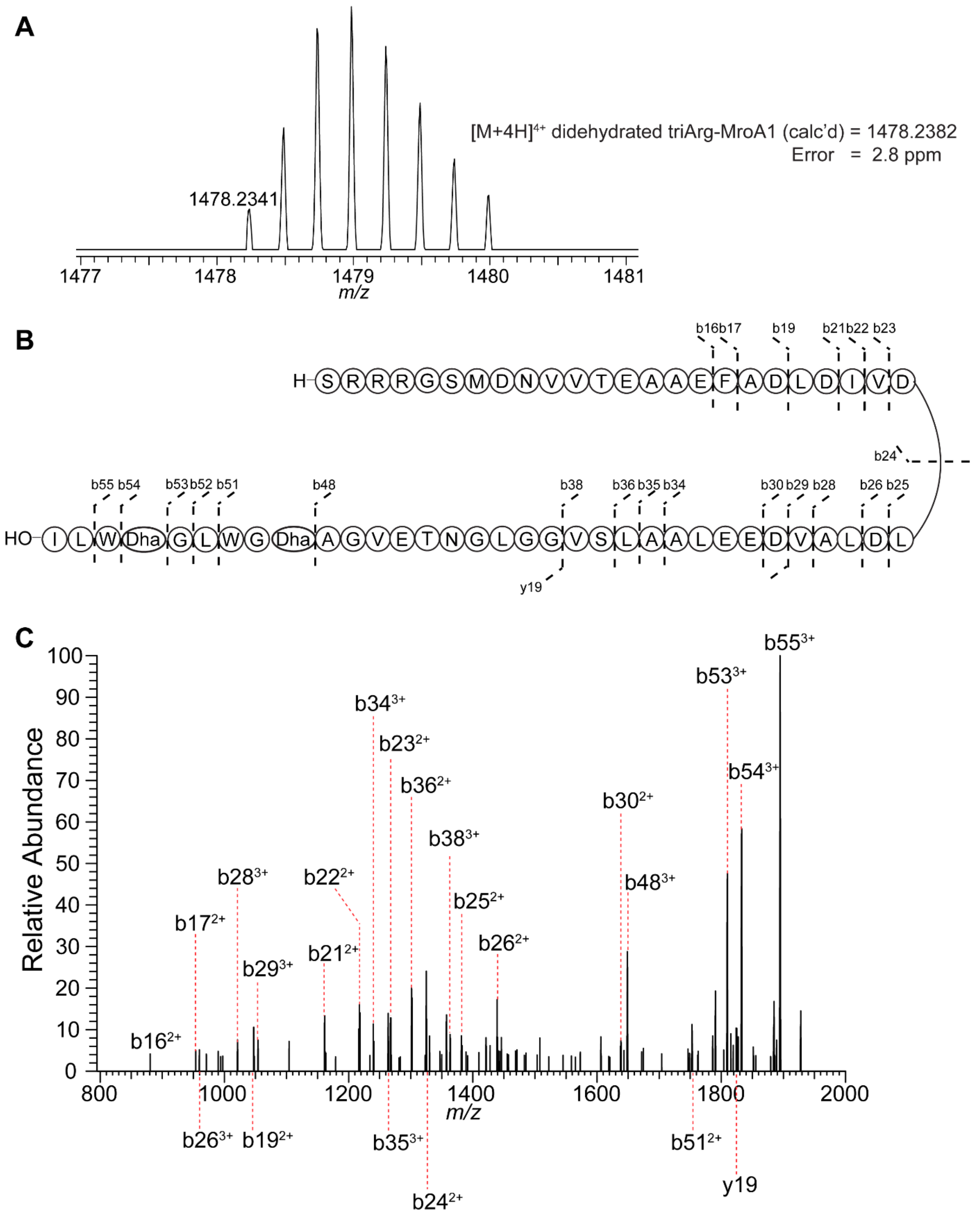

Figure S5: HR-ESI-MS/MS analysis of didehydrated MroA2. (A) HR-ESI-MS of didehydrated triArg-MroA2 formed after treatment of triArg-MroA2 with MroB/C. (B) Schematic representation of the bond cleavage of didehydrated triArg-MroA2 after CID. (C) HR-ESI-MS/MS of didehydrated triArg-MroA2 peptide after CID analysis. A table comparing observed and theoretical *m/z* values for fragments may be found in **Supplementary Dataset 2**.

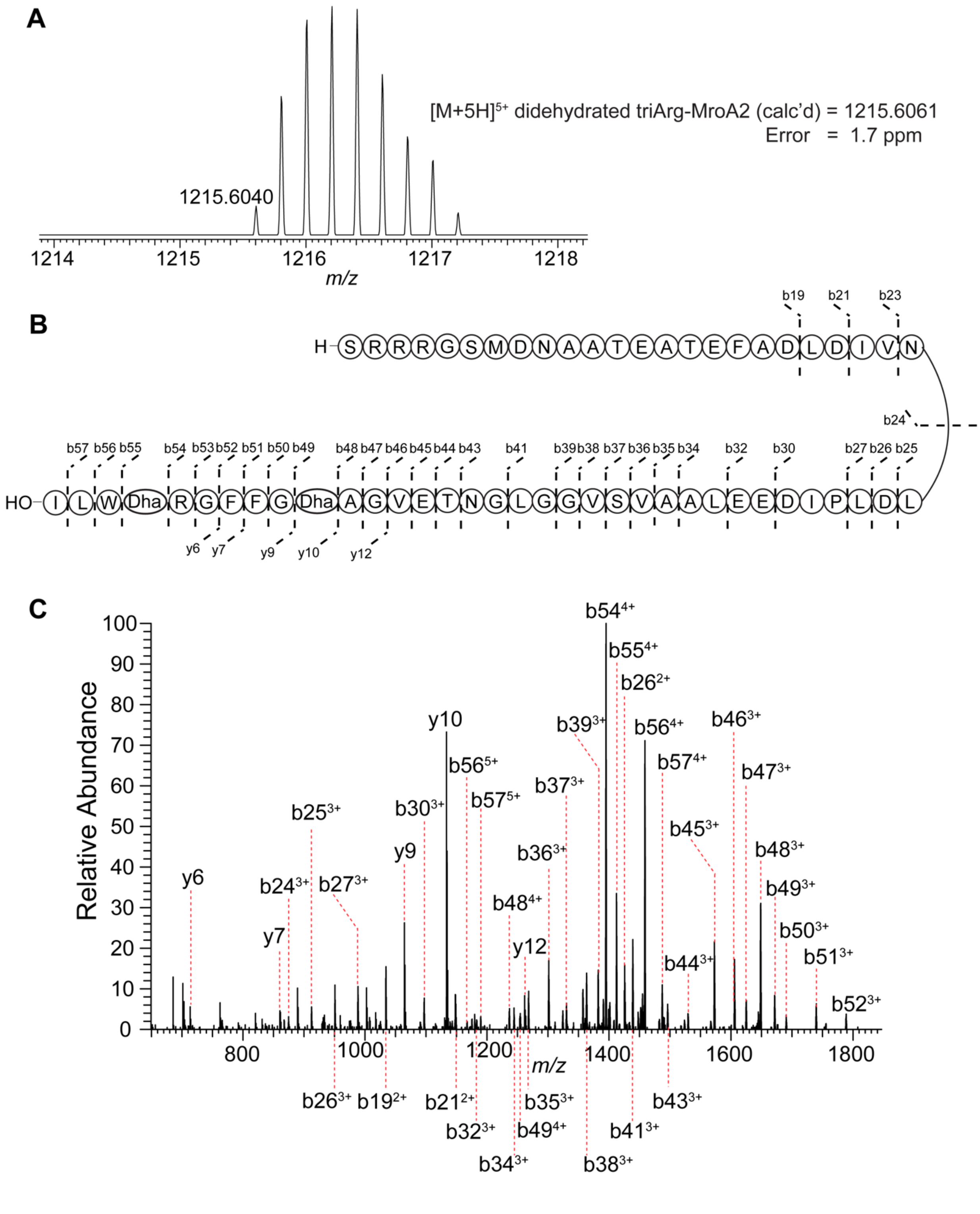

Figure S6: HR-ESI-MS/MS analysis of the eliminated carboxamide leader peptide of MroA1. (A) HR-ESI-MS of the carboxamide leader peptide formed after treatment of triArg-MroA1 with MroB/C/D. (B) Schematic representation of the bond cleavage of MroA1 carboxamide leader peptide after CID. (C) High-resolution tandem mass spectrum of MroA1 carboxamide leader peptide after CID analysis. A table comparing observed and theoretical *m/z* values for fragments may be found in **Supplementary Dataset 2.**

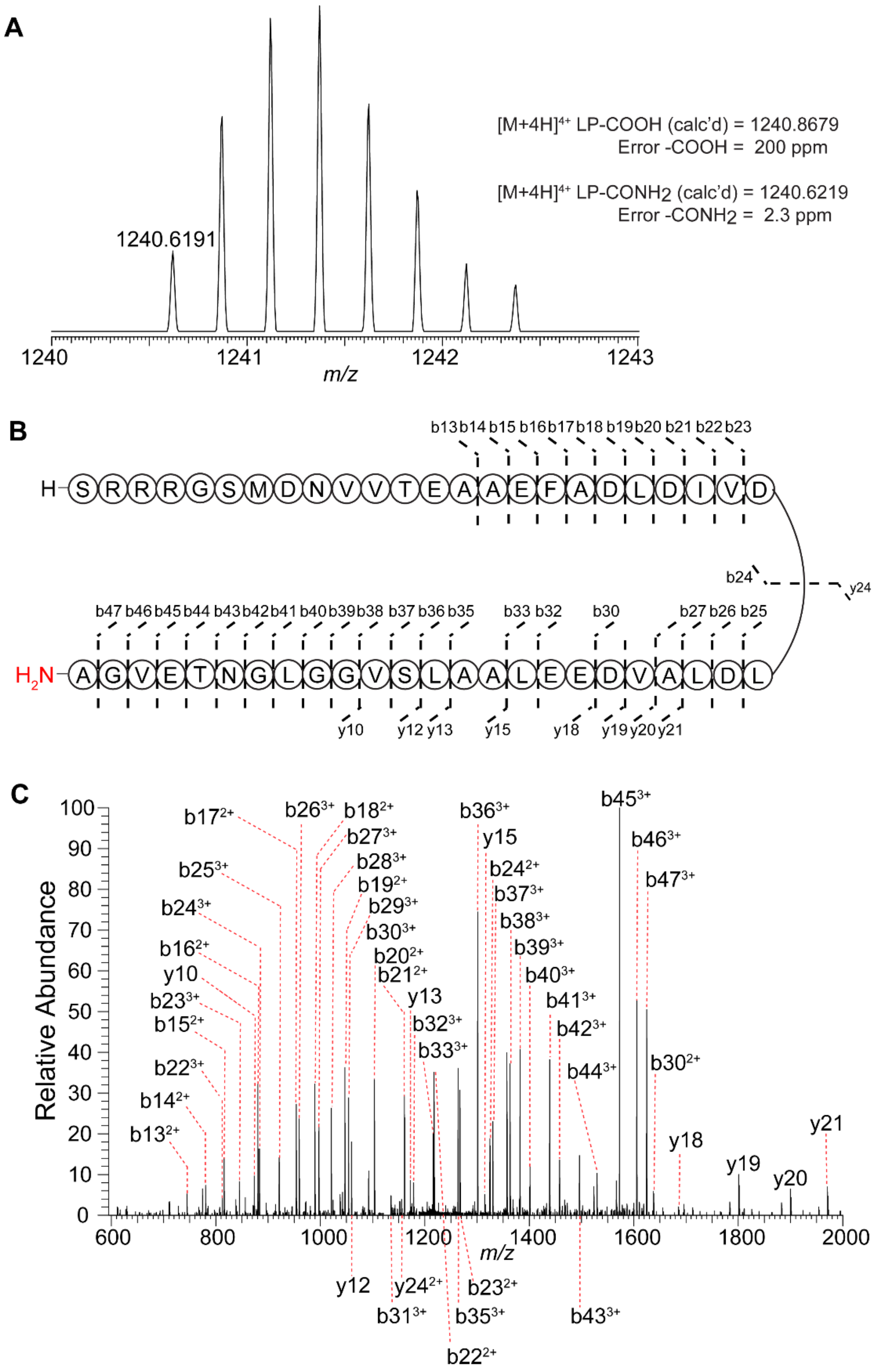

Figure S7: HR/MS/MS analysis of the eliminated carboxamide leader peptide of MroA2. (A) HR-ESI-MS of the carboxamide leader peptide formed after treatment of triArg-MroA2 with MroB/C/D. (B) Schematic diagram of the CID fragmentation pattern of MroA2 carboxamide leader peptide. (C) High-resolution tandem mass spectrum of MroA2 carboxamide leader peptide after CID analysis. A table comparing observed and theoretical *m/z* values for fragments may be found in **Supplementary Dataset 2**.

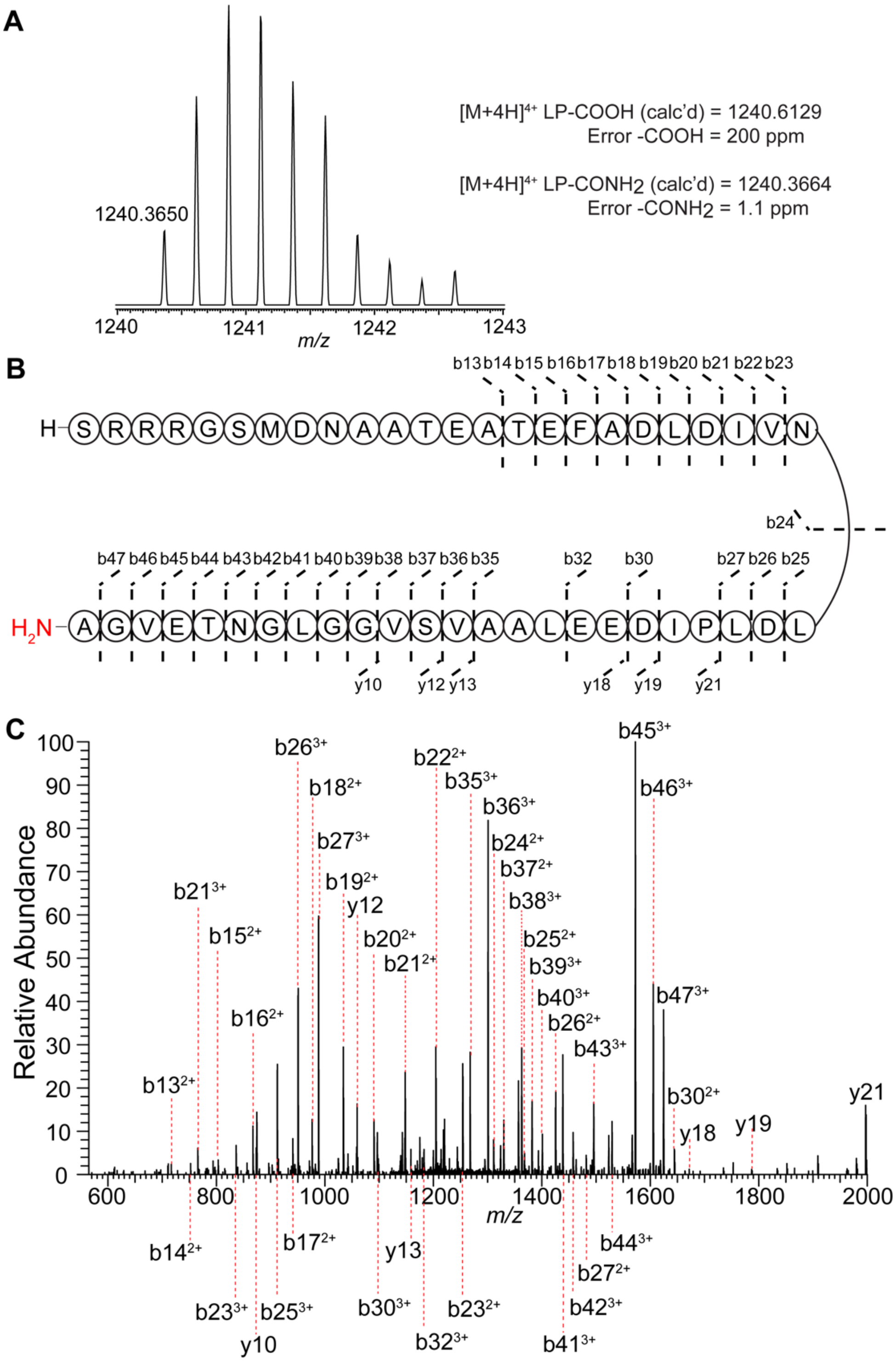

Figure S8: Analytical LC-MS analysis of enzymatically synthesized and chemically synthesized pyritide A1. Separate samples of enzymatically synthesized (blue) or chemically synthesized (orange) pyritide A1 were analyzed using reversed-phase analytical LC-MS on a C_{18} column. The structure of chemically synthesized pyritide A1 was verified with ^{1}H NMR spectroscopy in a previous study.[2] Coinjection of both samples yielded one symmetrical peak at the same retention time (black). Chromatograms shown below are extracted ion chromatograms at m/z = 933.4616, which corresponds to the *m/z* of $[M+H]^+$ of pyritide A1.

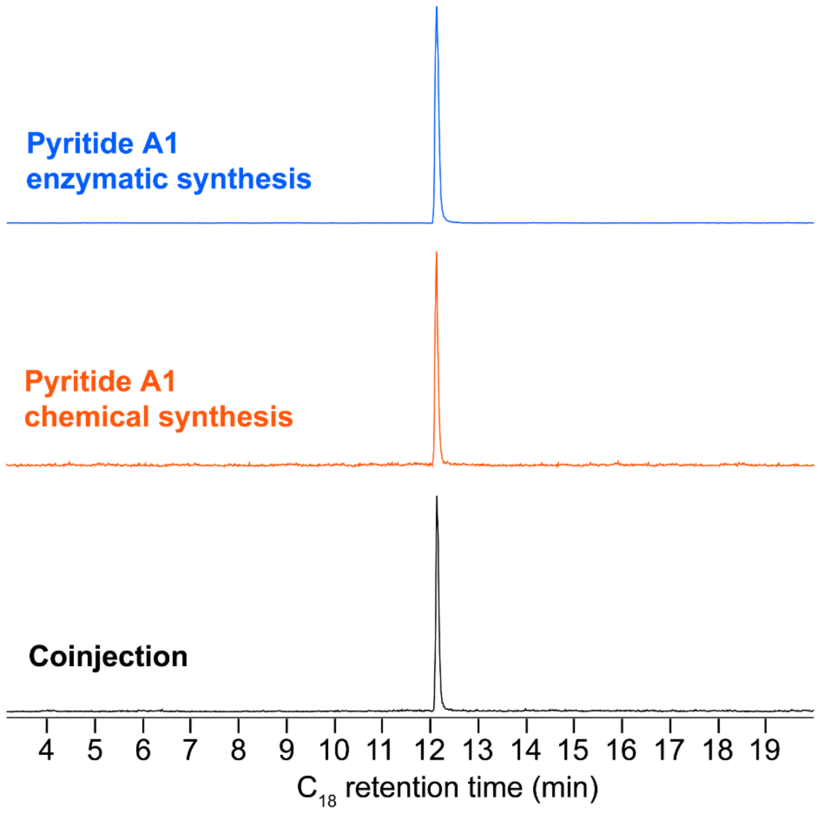

Figure S9: Tandem MS fragmentation of enzymatically and chemically synthesized pyritide A1. (A) Samples of pyritide A1 synthesized enzymatically or chemically were analyzed by CID. (B) Comparison of the CID patterns for enzymatically synthesized pyritide A1 (blue) or chemically synthesized pyritide A1 (orange). The structure of chemically synthesized pyritide A1 was verified by ^{1}H NMR spectroscopy in a previous study.[2] A table comparing observed and theoretical *m/z* values for fragments may be found in **Supplementary Dataset 2**.

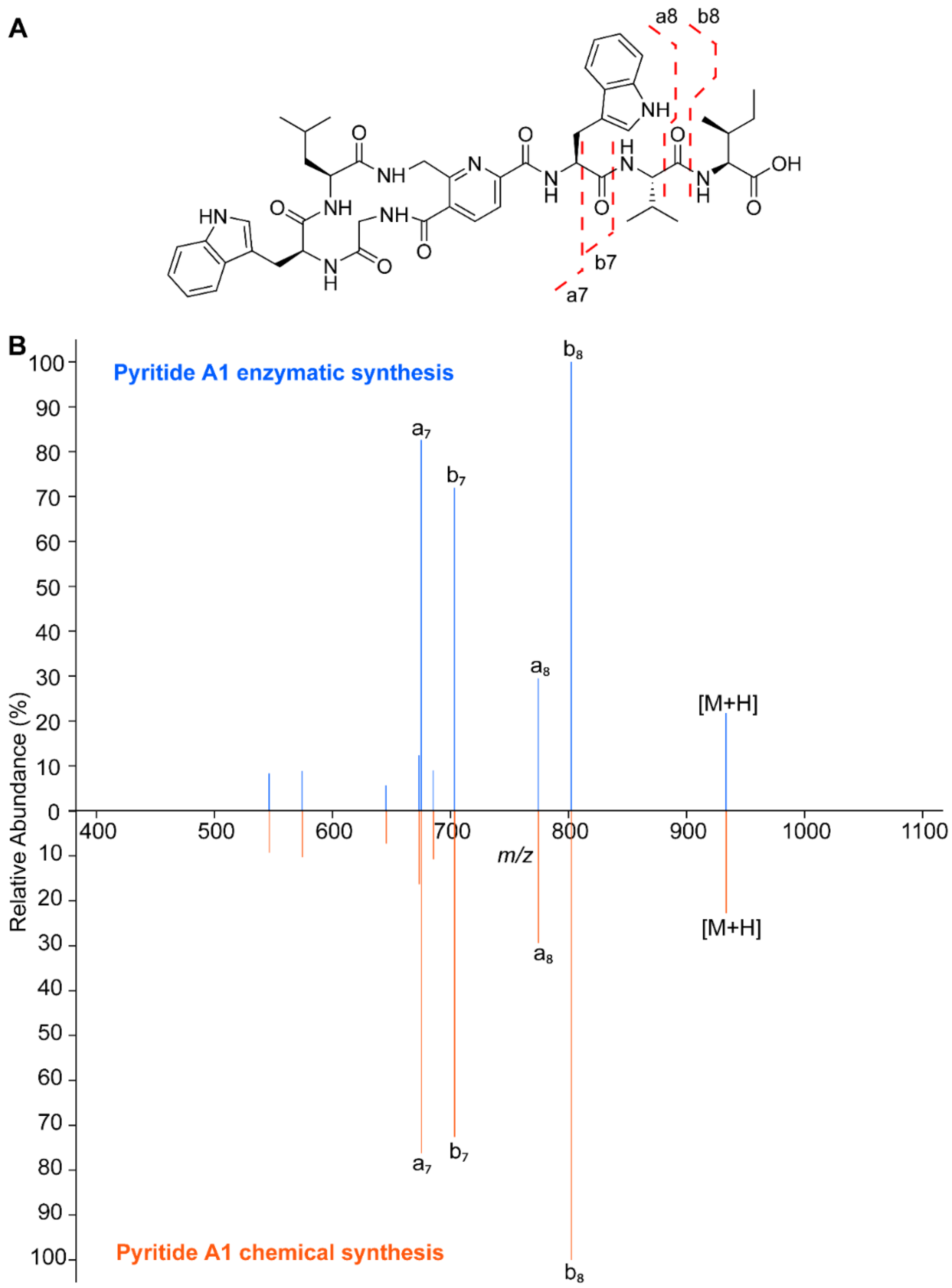

Figure S10: Analytical LC-MS analysis of enzymatically synthesized and chemoenzymatically synthesized pyritide A2. Samples of enzymatically synthesized (blue) or chemoenzymatically synthesized (orange) pyritide A2 were analyzed using reversed-phase analytical HPLC on a C_{18} column. The structure of chemoenzymatically synthesized pyritide A2 was verified with ^1H NMR spectroscopy in a previous study.[2] Coinjection of both samples yielded one symmetrical peak at the same retention time (black). Chromatograms shown below are extracted ion chromatograms at m/z = 549.7826, which corresponds to the *m/z* of $[M+2H]^{2+}$ of pyritide A2.

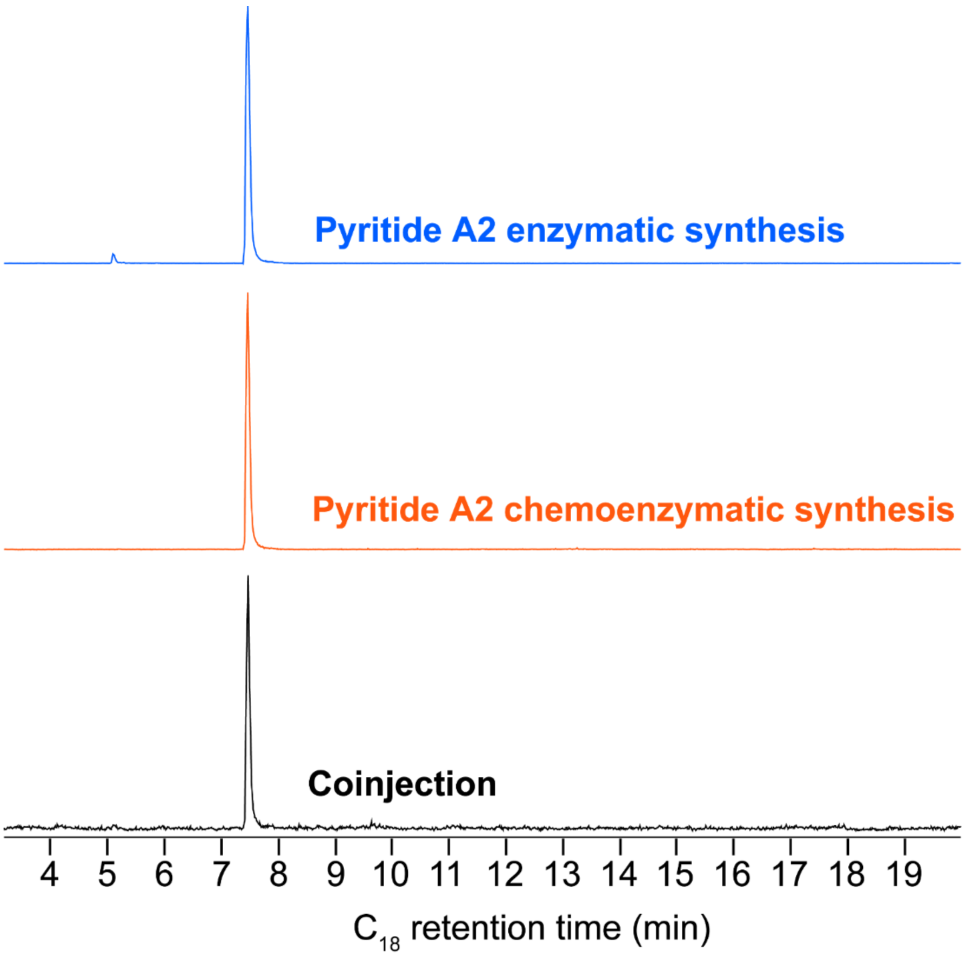

Figure S11: Tandem MS fragmentation of enzymatically and chemoenzymatically synthesized pyritide A2. (A) Samples of pyritide A1 synthesized enzymatically or chemoenzymatically were analyzed by CID. (B) Comparison of the CID patterns for enzymatically synthesized pyritide A2 (blue) or chemoenzymatically synthesized pyritide A2 (orange). The structure of chemically synthesized pyritide A2 was verified by ^{1}H NMR spectroscopy in a previous study.[2] A table comparing observed and theoretical *m/z* values for fragments may be found in **Supplementary Dataset 2**.

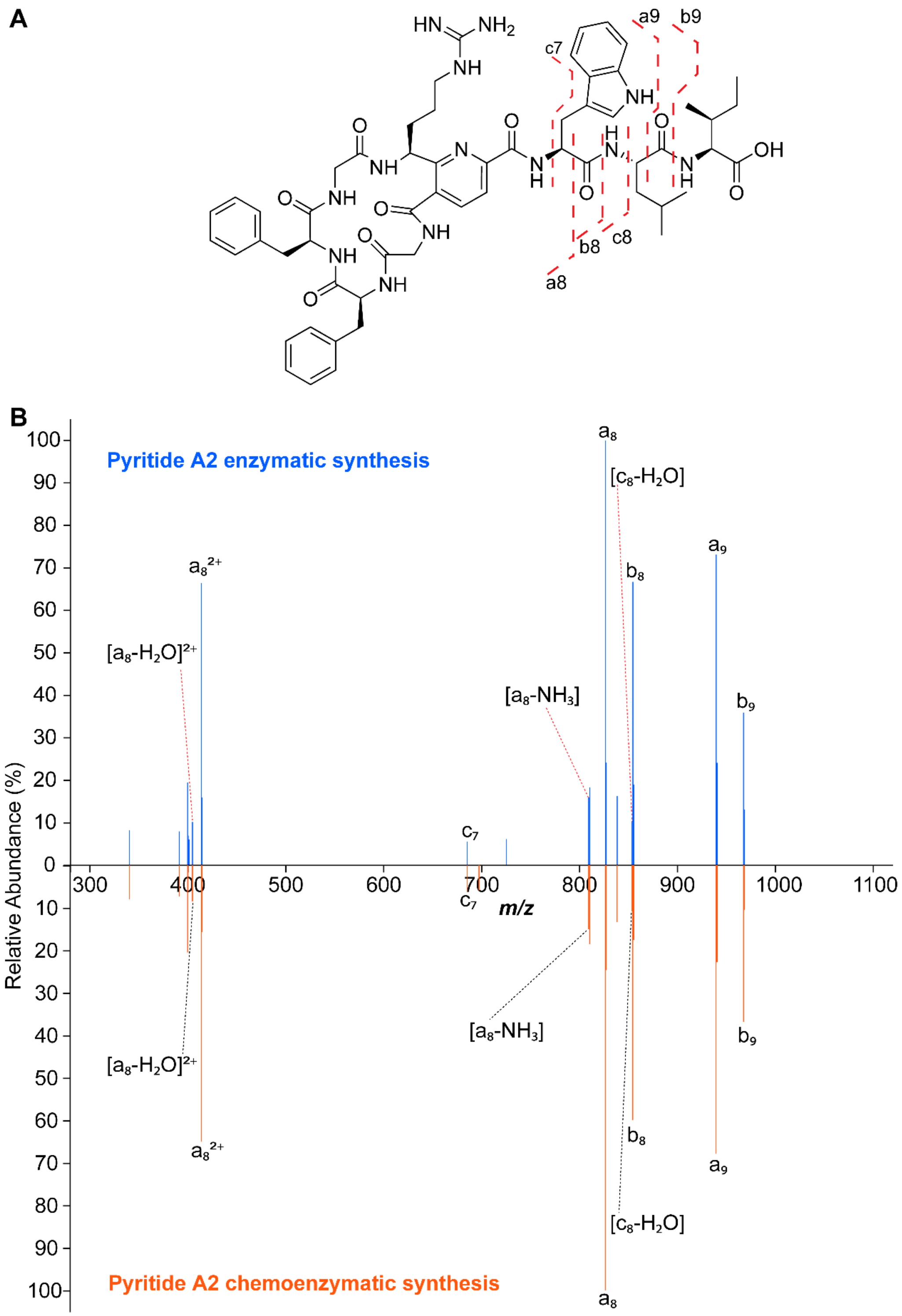

Figure S12: Phe3, Phe4, and Gly5 variants of MroA2 undergo two dehydrations after MroB/C treatment. All spectra were acquired using reflector positive mode MALDI-TOF-MS. Unless otherwise stated, all peaks are $[M+H]^{+}$. The precursor peptides were generated using *in vitro* translation (see Experimental Methods). The f in the precursor peptide sequence represents a formyl group resulting from formyl-methionine utilized in *in vitro* translation. The unreacted precursor peptides are colored blue and the didehydrated peptides are colored yellow. The # mark represents a -17 Da artifact resulting from deamination specific to reflector positive mode in MALDI-TOF-MS.[13, 14]

345

f-MRRRGSMDNVVTEAAEFADLDIVDLDLAVDEELAALSVGGLGNTEVGA|SGFFGRSWLI

Leader peptide

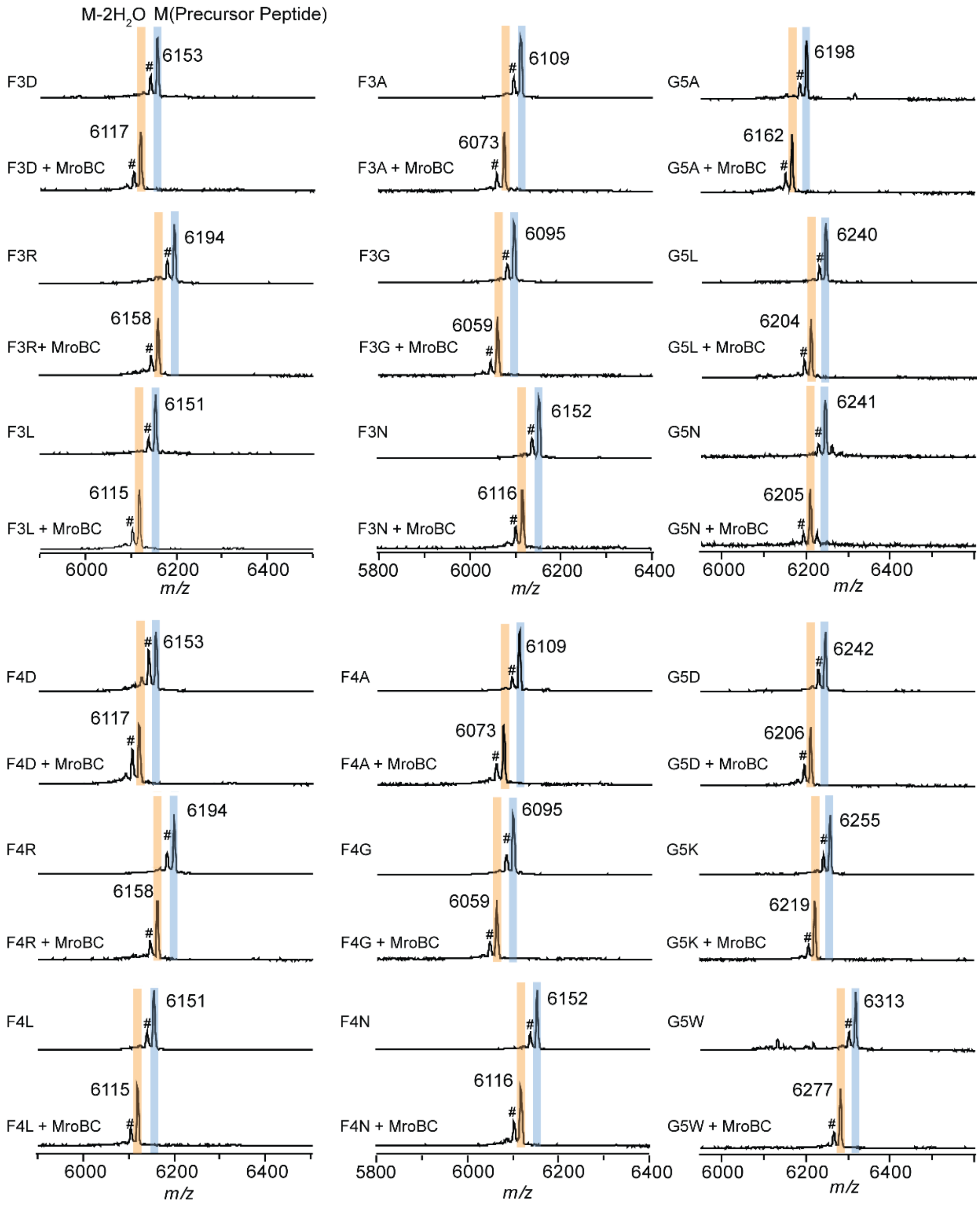

Figure S13: Gly2 and Leu9 variants of MroA2 undergo two dehydrations after MroB/C treatment. All spectra were acquired using reflector positive mode MALDI-TOF-MS. Unless otherwise stated, all peaks are $[M+H]^+$. The precursor peptides were generated through *in vitro* translation (see Experimental Methods). The f in the precursor peptide sequence represents a formyl group, which results from formyl-methionine utilized in *in vitro* translation. The unreacted precursor peptides are colored blue and the didehydrated peptides are colored yellow. The # mark represents a -17 Da artifact resulting from deamination specific to reflector positive mode in MALDI-TOF-MS.[13, 14]

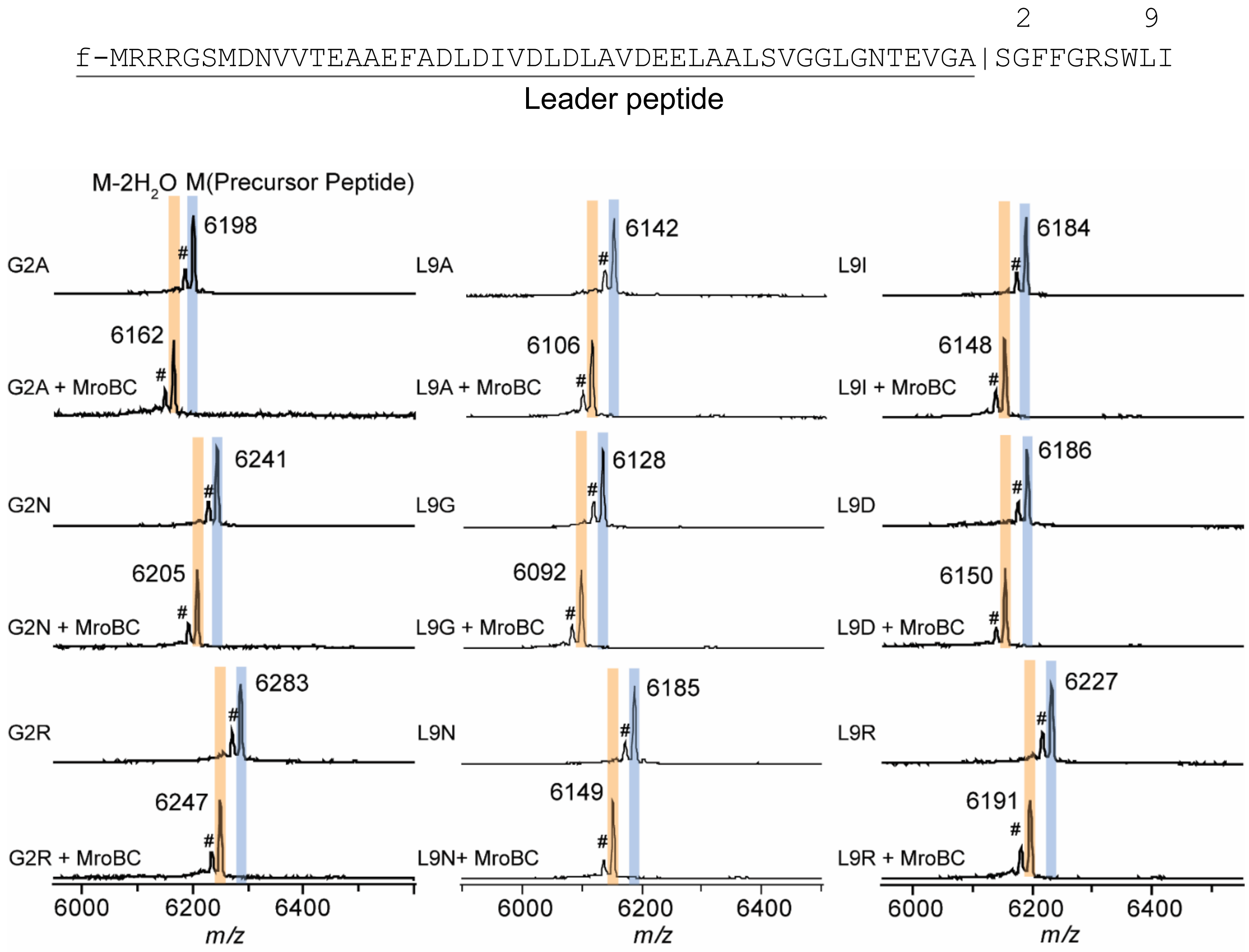

Figure S14: Arg6, Trp8, and Ile10 variants of MroA2 undergo two dehydrations after MroB/C treatment. All spectra were acquired by reflector positive mode MALDI-TOF-MS. Unless otherwise stated, all peaks are $[M+H]^+$. The precursor peptides were generated through *in vitro* translation (see Experimental Methods). The f in the precursor peptide sequence represents a formyl group, which results from formyl-methionine utilized in *in vitro* translation. The unreacted precursor peptides are colored blue and the didehydrated peptides are colored yellow. The # marks represent -17 Da artifacts resulting from deamination specific to reflector positive mode MALDI-TOF-MS.[13, 14] The x mark represents a minor +211 Da adduct caused by the addition of DTT to Dha residues in the peptide and carboxymethylation of the DTT adduct by IAA.

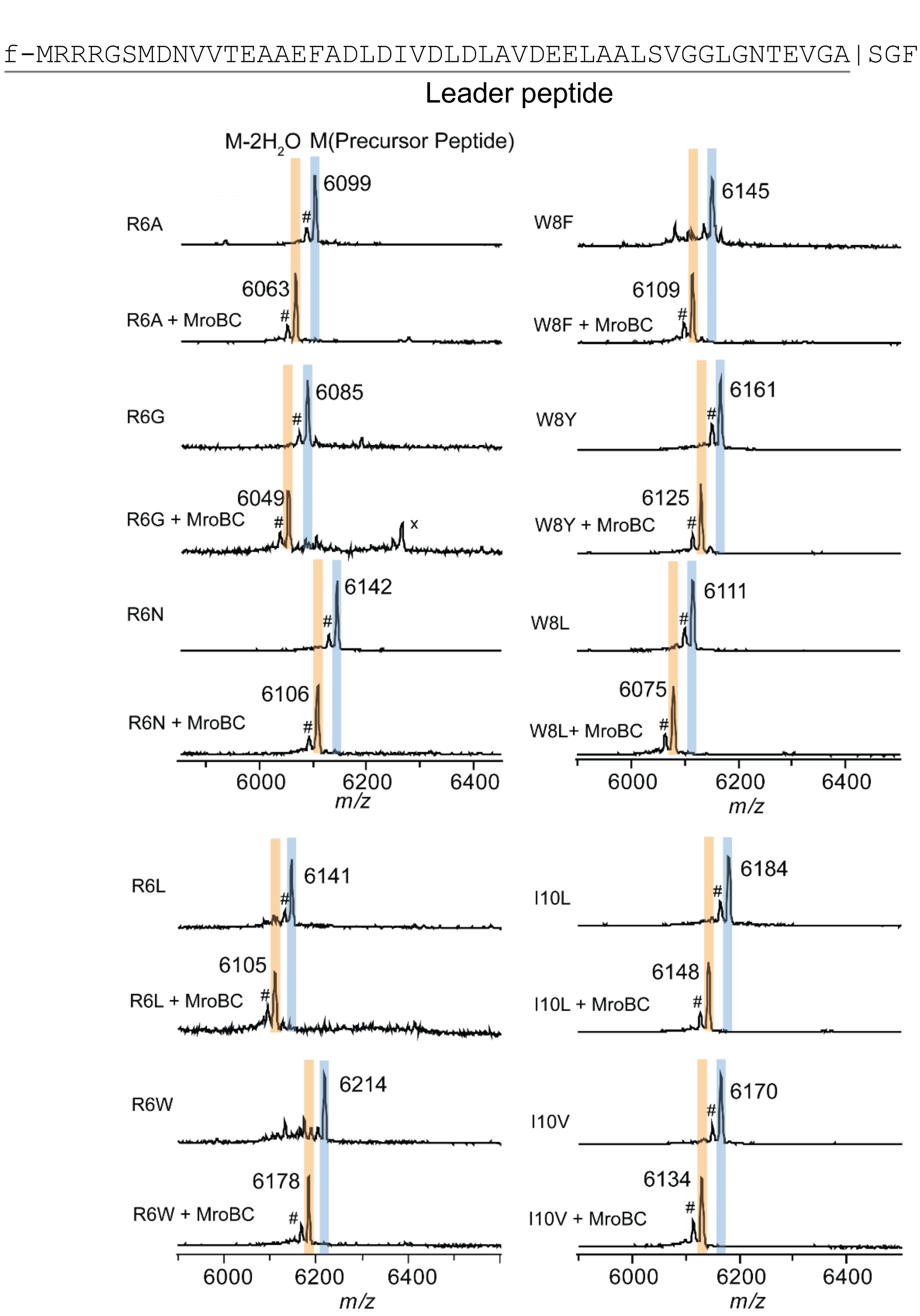

Figure S15: Gly2, Trp8, and Ile10 variants of MroA2 undergo incomplete dehydrations after MroB/C treatment. All spectra were acquired using linear positive mode MALDI-TOF-MS. Unless otherwise stated, all peaks are $[M+H]^+$. The precursor peptides were generated through *in vitro* translation (see Experimental Methods). The f in the precursor peptide sequence represents a formyl group, which results from formyl-methionine utilized in *in vitro* translation. The *m/z* values indicated represent the observed centroid *m/z* values. The unreacted precursor peptides are colored blue, the monodehydrated peptides are colored red and the didehydrated peptides are colored yellow.

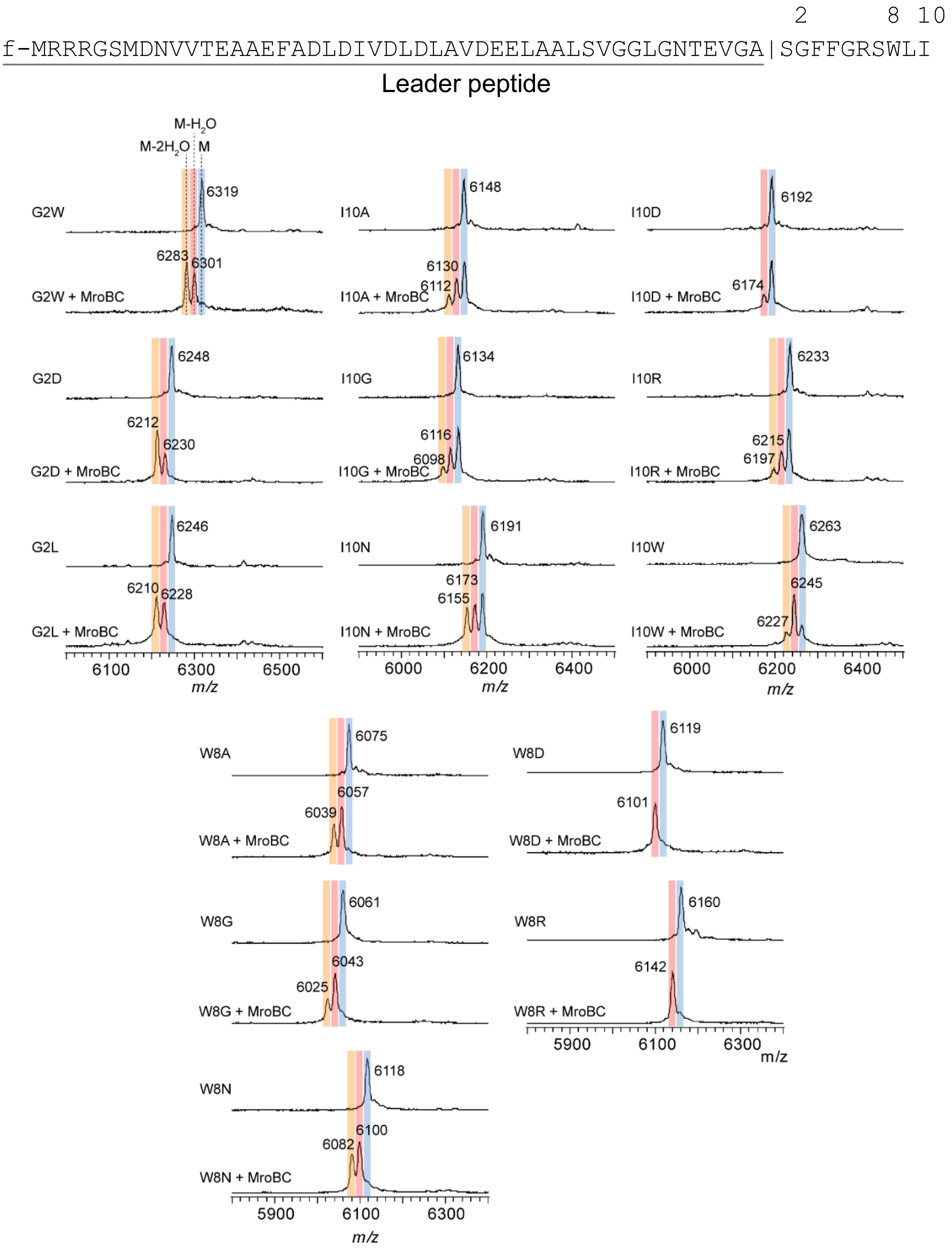

Figure S16: MALDI-TOF-MS analysis of Phe3 and Phe4 variants of MroA2 after MroB/C/D treatment. All spectra were acquired using reflector positive mode MALDI-TOF-MS. Unless otherwise stated, all peaks are $[M+H]^+$. The precursor peptides were generated through *in vitro* translation (see Experimental Methods). The f in the precursor peptide sequence represents a formyl group, which results from formyl-methionine utilized in *in vitro* translation. The pyritide macrocycles and the ejected leader peptides are annotated accordingly. The single asterisk (*) and the double-asterisk (**) in the mass spectra represent +16 Da and +32 Da species caused by oxidation during sample preparation.[15]

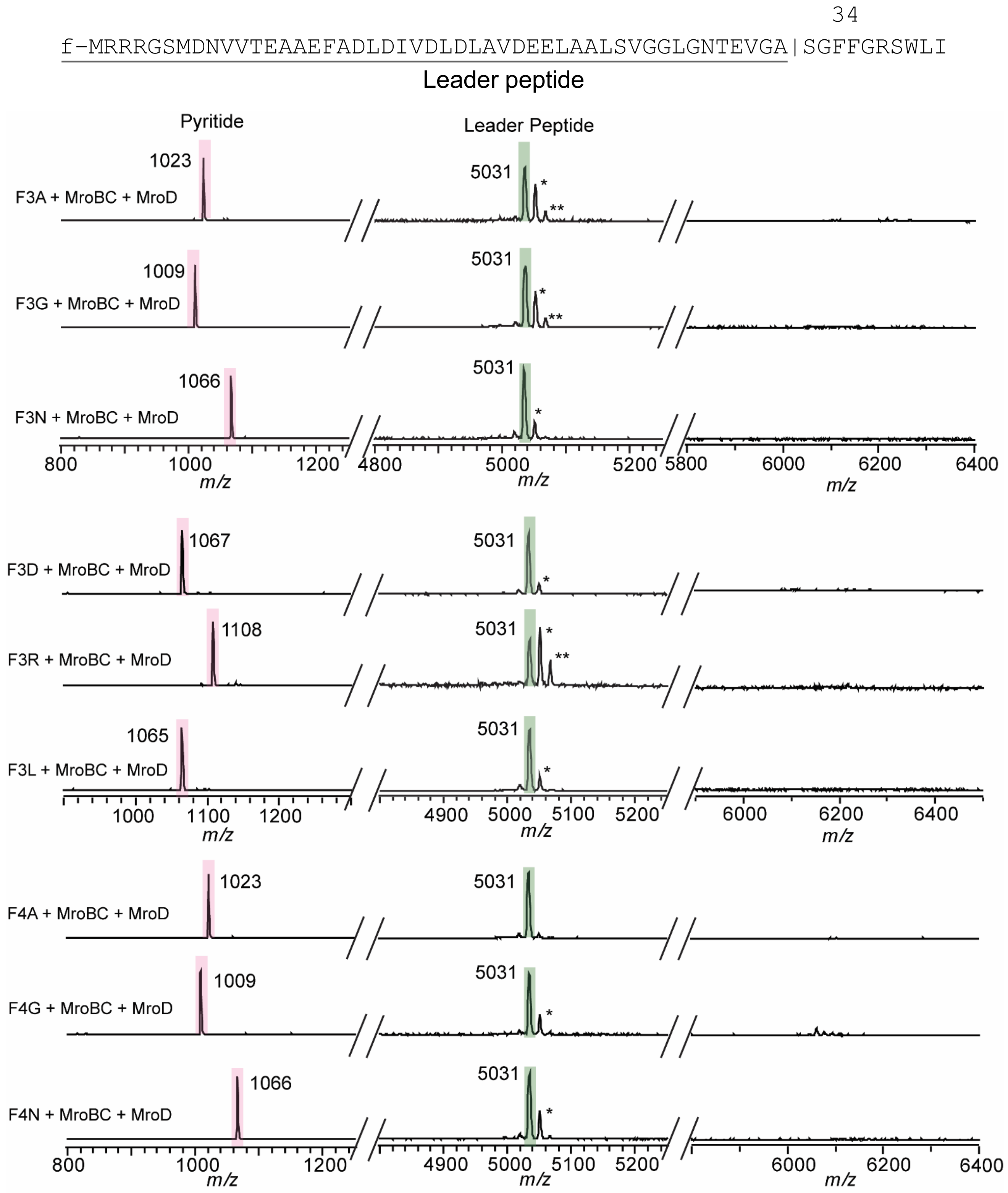

Figure S17: MALDI-TOF-MS analysis of Phe4 and Gly5 variants of MroA2 after MroB/C/D treatment. All spectra were acquired using reflector positive mode MALDI-TOF-MS. Unless otherwise stated, all peaks are $[M+H]^+$. The precursor peptides were generated through *in vitro* translation (see Experimental Methods). The f in the precursor peptide sequence represents a formyl group, which results from formyl-methionine utilized in *in vitro* translation. The pyritide macrocycles, the ejected leader peptides, and the remaining didehydrated intermediates are annotated accordingly. The single asterisk (*) and the double-asterisk (**) in the mass spectra represent +16 Da and +32 Da species caused by oxidation during sample preparation.[15]

45
f-MRRRGSMDNVVTEAAEFADLDIVDLDLAVDEELAALSVGGLGNTEVGA|SGFFGRSWLI
Leader peptide

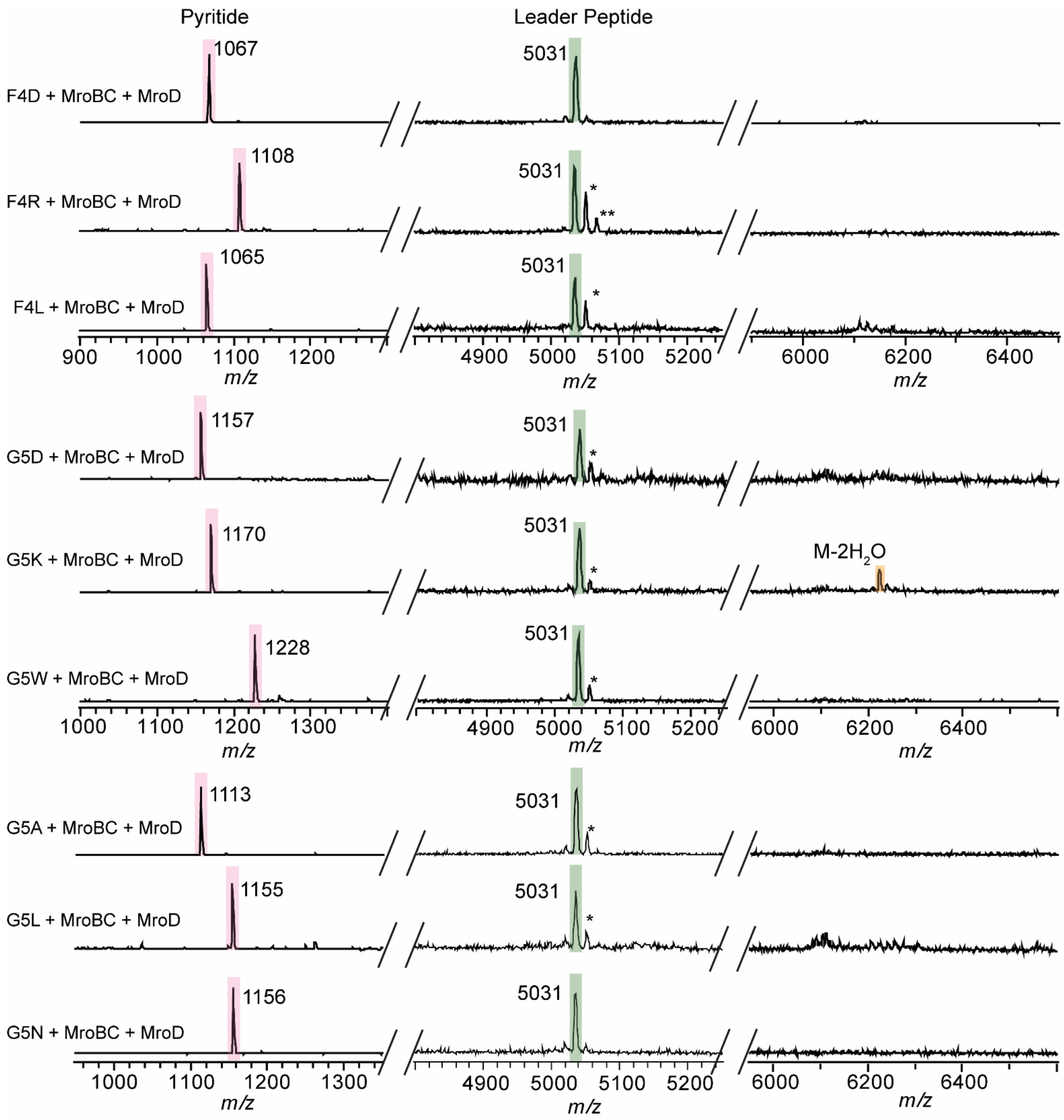

Figure S18: MALDI-TOF-MS analysis of Arg6 and Gly2 variants of MroA2 after MroB/C/D treatment. All spectra were acquired using reflector positive mode or negative mode MALDI-TOF-MS. Unless otherwise stated, all peaks are $[M+H]^+$. The precursor peptides were generated through *in vitro* translation (see Experimental Methods). The f in the precursor peptide sequence represents a formyl group, which results from formyl-methionine utilized in *in vitro* translation. The pyritide macrocycles, the ejected leader peptides, and the remaining didehydrated and monodehydrated intermediates are annotated accordingly. The # marks in the mass spectra represent -17 Da artifacts resulting from deamination specific to reflector positive mode in MALDI-TOF-MS.[13, 14] The single asterisk (*) in the mass spectra denotes +16 Da species caused by oxidation during sample preparation.[15]

2 6

f-MRRRGSMDNVVTEAAEFADLDIVDLDLAVDEELAALSVGGLGNTEVGA|SGFFGRSWLI

Leader peptide

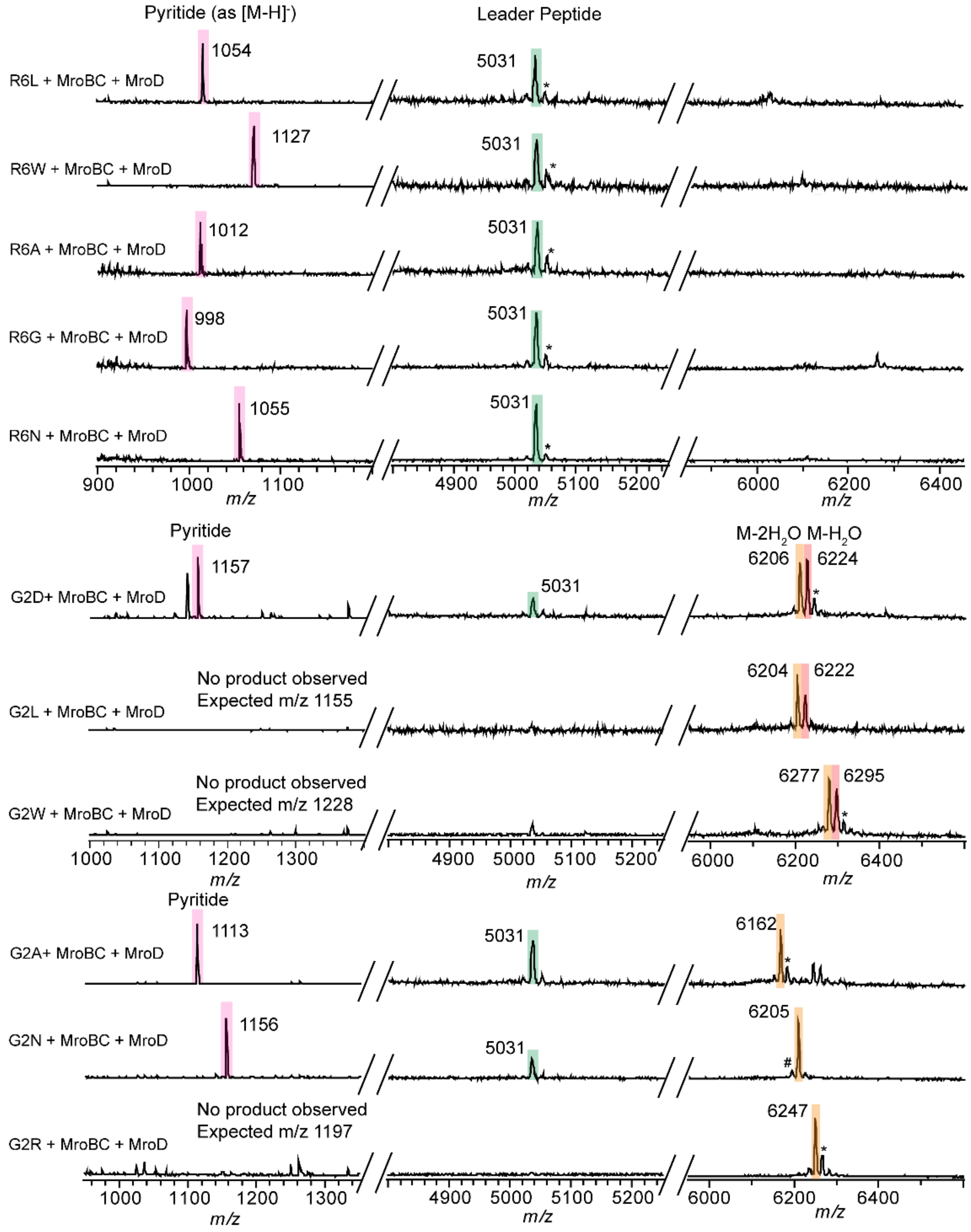

Figure S19: MALDI-TOF-MS analysis of Trp8 variants of MroA2 after MroB/C/D treatment. All spectra were acquired using reflector positive mode MALDI-TOF-MS. Unless otherwise stated, all peaks are $[M+H]^+$. The precursor peptides were generated through *in vitro* translation (see Experimental Methods). The f in the precursor peptide sequence represents a formyl group, which results from formyl-methionine utilized in *in vitro* translation. The pyritide macrocycles, the ejected leader peptides, and the remaining didehydrated and monodehydrated intermediates are annotated accordingly. The single asterisk (*) in the mass spectra represents +16 Da species caused by oxidation during sample preparation.[15]

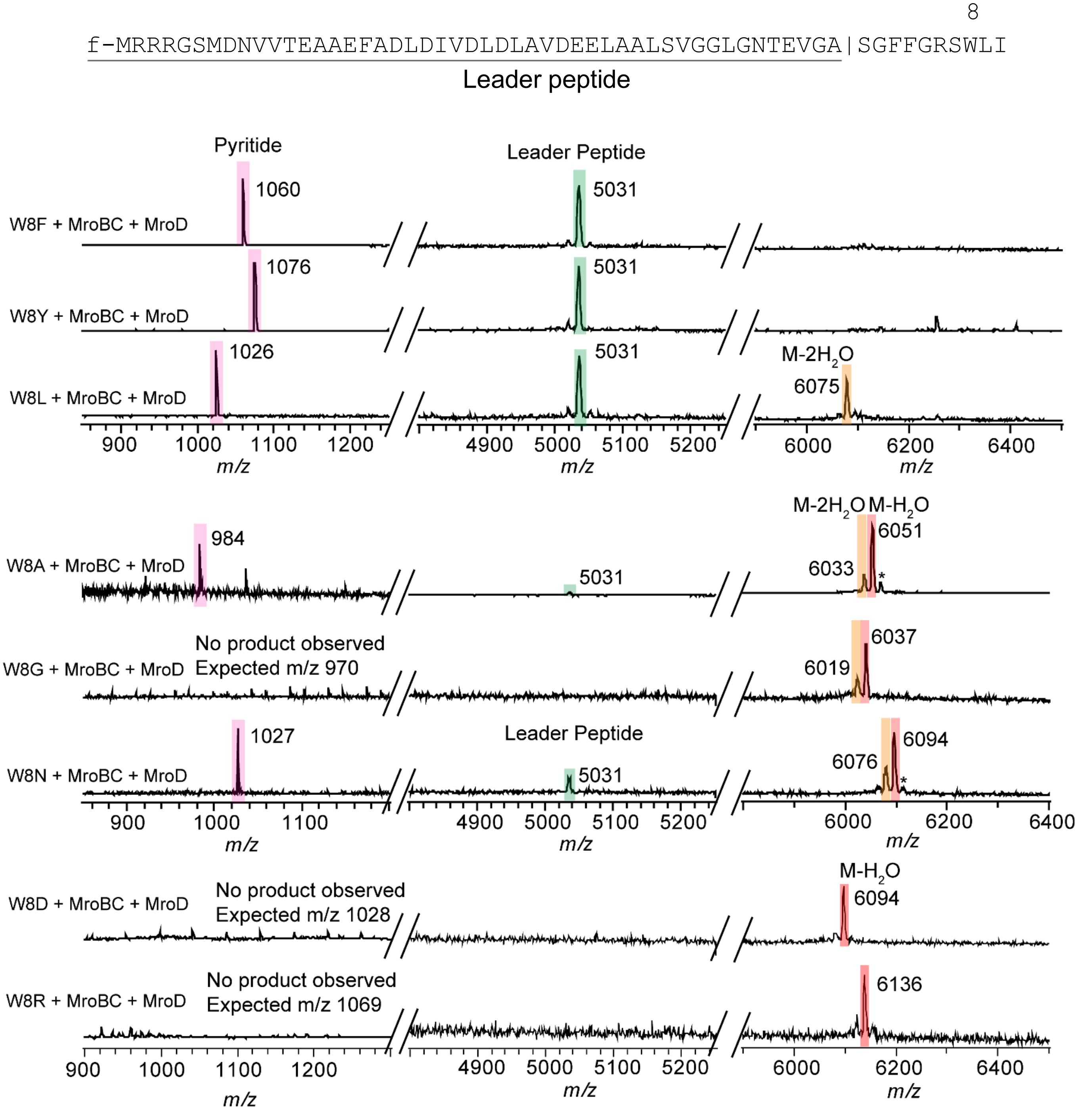

Figure S20: MALDI-TOF-MS analysis of Leu9 variants of MroA2 after MroB/C/D treatment. All spectra were acquired using reflector positive mode MALDI-TOF-MS in MALDI-TOF-MS. The crystallization matrix utilized in this experiment was Super DHB. Unless otherwise stated, all peaks are $[M+H]^+$. The precursor peptides were generated through *in vitro* translation (see Experimental Methods). The f in the precursor peptide sequence represents a formyl group, which results from formyl-methionine utilized in *in vitro* translation. The pyritide macrocycles, the ejected leader peptides, and the remaining didehydrated and monodehydrated intermediates are annotated accordingly. The single asterisk (*) in the mass spectra represents +16 Da species caused by oxidation during sample preparation.[15]

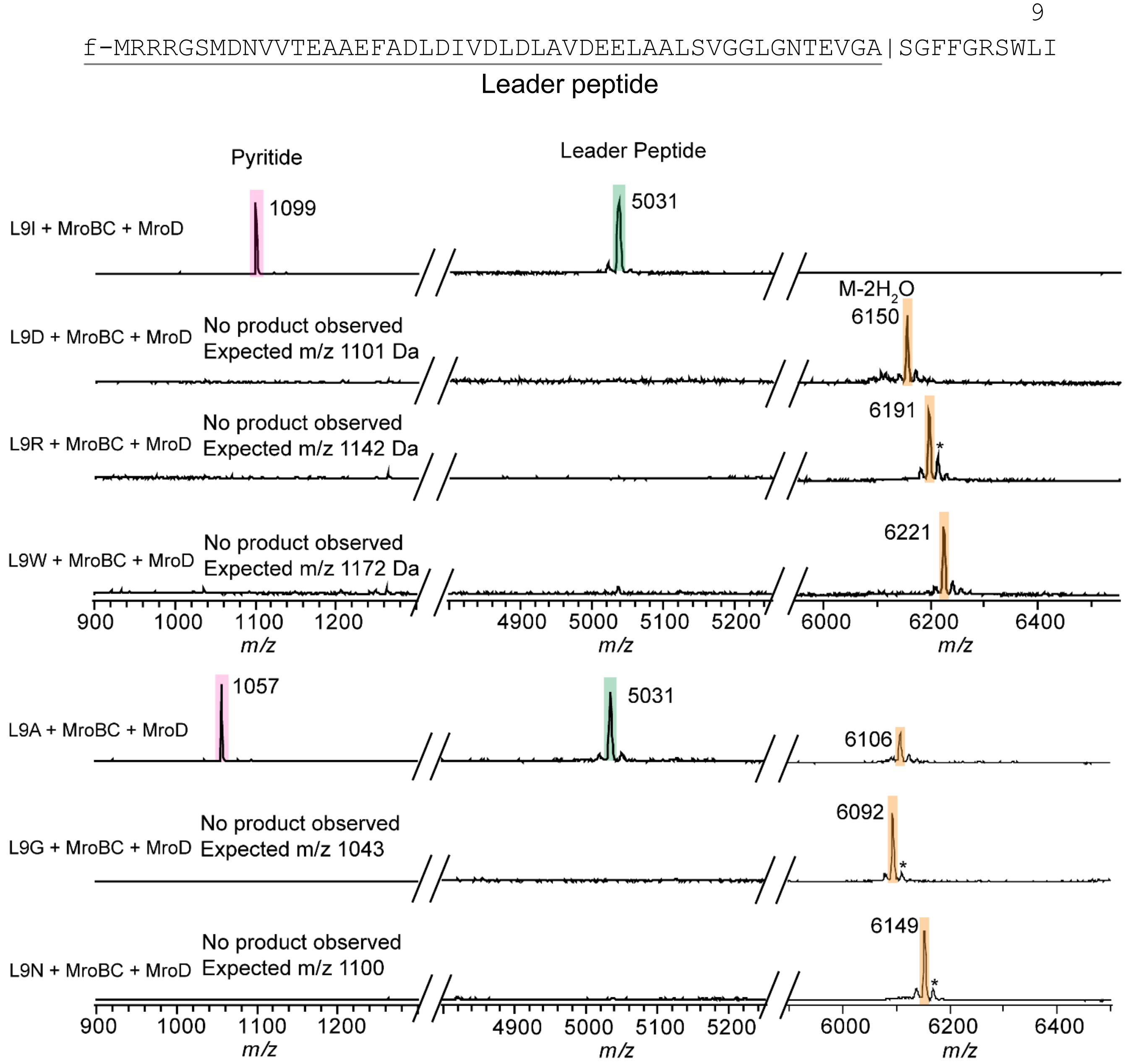

Figure S21: MALDI-TOF-MS analysis of Ile10 variants of MroA2 after MroB/C/D treatment. All spectra were acquired using reflector positive mode MALDI-TOF-MS. The crystallization matrix utilized in this experiment was Super DHB. Unless otherwise stated, all peaks are $[M+H]^+$. The precursor peptides were generated through *in vitro* translation (see Experimental Methods). The f in the precursor peptide sequence represents a formyl group, which results from formyl-methionine utilized in *in vitro* translation. The pyritide macrocycles, the ejected leader peptides, and the remaining didehydrated, monodehydrated and nondehydrated intermediates are annotated accordingly. The single asterisk (*) in the mass spectra represents +16 Da species caused by oxidation during sample preparation. [15]

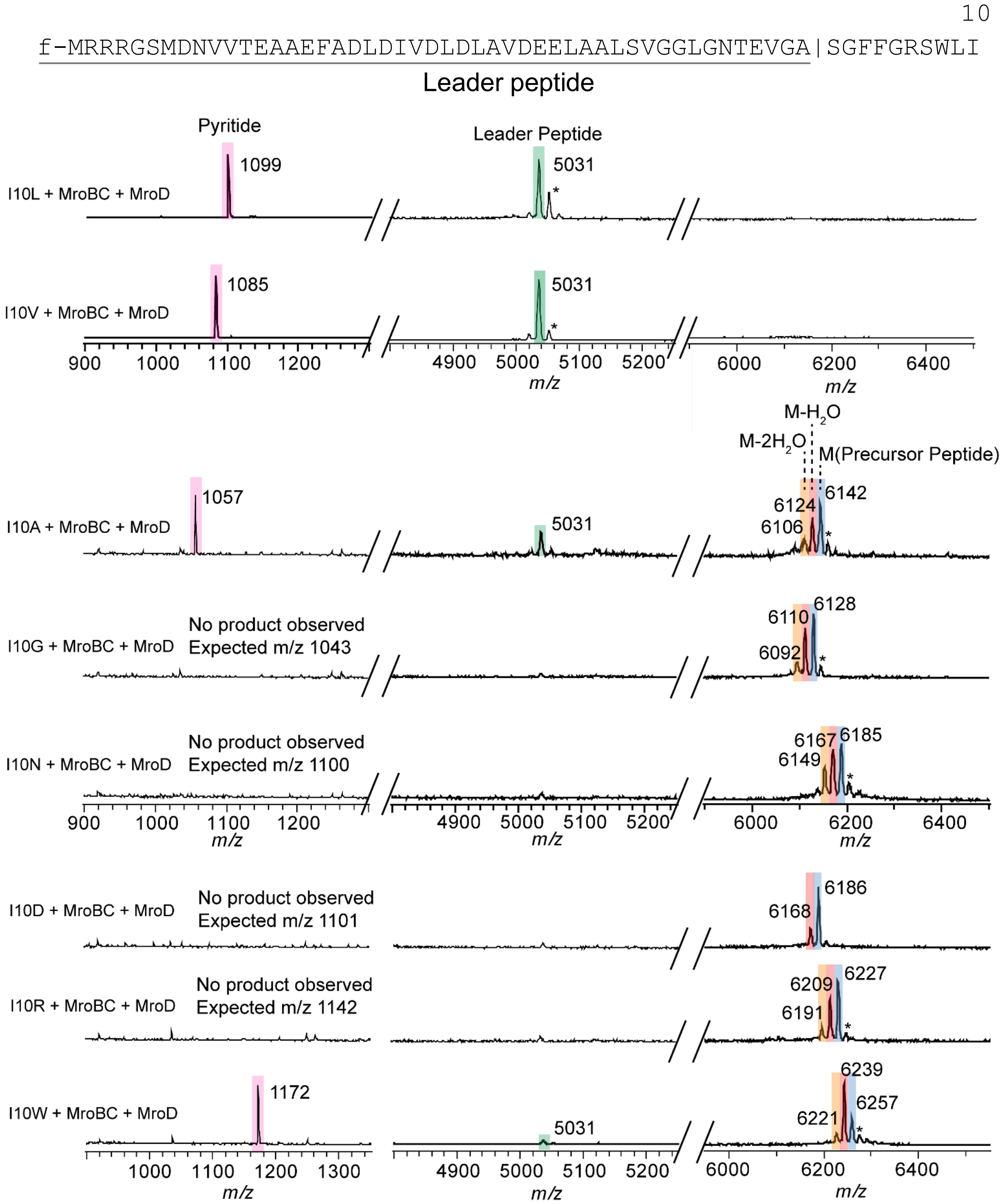

Table S5: Summary of MroB/C/D activity on MroA2 single core peptide variants. MroD relative activity was qualitatively estimated by comparing the intensity of leader peptide and remaining didehydrated intermediates. +++ indicates enzyme activity roughly equal to wild-type MroA2 (major species are ejected leader peptides and produced macrocycles; insignificant amount of remaining intermediates are observed); ++ indicates modestly reduced enzyme activity (both significant amount of ejected leader peptides and intermediates are observed); + indicates severely reduced enzyme activity (a high-intensity peak of remaining didehydrated intermediate, a low-intensity peak of ejected leader peptide, and observable produced macrocycles); - indicates no detectable enzyme activity (no macrocycles observed); N/A = not applicable.

Variation	Dha formation	Relative MroD conversion	Variation	Dha formation	Relative MroD conversion
G2A	2 Dha	++	W8Y	2 Dha	+++
G2N	2 Dha	+	W8F	2 Dha	+++
G2L	Mix of 1,2 Dha	-	W8L	2 Dha	++
G2D	Mix of 1,2 Dha	+	W8A	Mix of 1,2 Dha	+
G2R	2 Dha	-	W8G	Mix of 1,2 Dha	+
G2W	Mix of 1,2 Dha	-	W8N	Mix of 1,2 Dha	+
F3A	2 Dha	+++	W8D	1 Dha	N/A (No 2 Dha formation)
F3N	2 Dha	+++	W8R	1 Dha	N/A (No 2 Dha formation)
F3L	2 Dha	+++	L9I	2 Dha	+++
F3D	2 Dha	+++	L9A	2 Dha	++
F3R	2 Dha	+++	L9G	2 Dha	-
F3G	2 Dha	+++	L9N	2 Dha	-
F4A	2 Dha	+++	L9W	2 Dha	-
F4N	2 Dha	+++	L9D	2 Dha	-
F4L	2 Dha	+++	L9R	2 Dha	-
F4D	2 Dha	+++	I10L	2 Dha	+++
F4R	2 Dha	+++	I10V	2 Dha	+++
F4G	2 Dha	+++	I10A	Mix of 0,1,2 Dha	+
G5A	2 Dha	+++	I10G	Mix of 0,1,2 Dha	-
G5N	2 Dha	+++	I10N	Mix of 0,1,2 Dha	-
G5L	2 Dha	+++	I10W	Mix of 0,1,2 Dha	-
G5D	2 Dha	+++	I10D	Mix of 0,1,2 Dha	N/A (No 2 Dha formation)
G5K	2 Dha	+++	I10R	Mix of 0,1,2 Dha	-
G5W	2 Dha	+++			
R6A	2 Dha	+++			
R6N	2 Dha	+++			
R6L	2 Dha	+++			
R6G	2 Dha	+++			
R6W	2 Dha	+++			

Figure S22: Trp8 and Ile10 variants of MroA2 MALDI-TOF-MS undergo inefficient cyclization by MroD. MALDI-TOF-MS analysis of MroA2 S1C/S7C, MroA2 S1C/S7C/W8G, and S1C/S7C/I10G reacted with MroD after Dhas were installed by chemical dehydrothiolation using methyl 2,5-dibromopentanoate (MDBP). All spectra were acquired using reflector positive mode MALDI-TOF-MS. Unless otherwise stated, all peaks are $[M+H]^+$. The pyritide macrocycles, the ejected leader peptides, the didehydrothiolated intermediates, and the unmodified precursor peptides are annotated accordingly. The # mark in the mass spectrum represents a -17 Da MALDI artifact, a result of deamination specific to reflector positive mode in MALDI-TOF-MS.[13,14] Cys residues colored brown underwent dehydrothiolation to form Dhas.

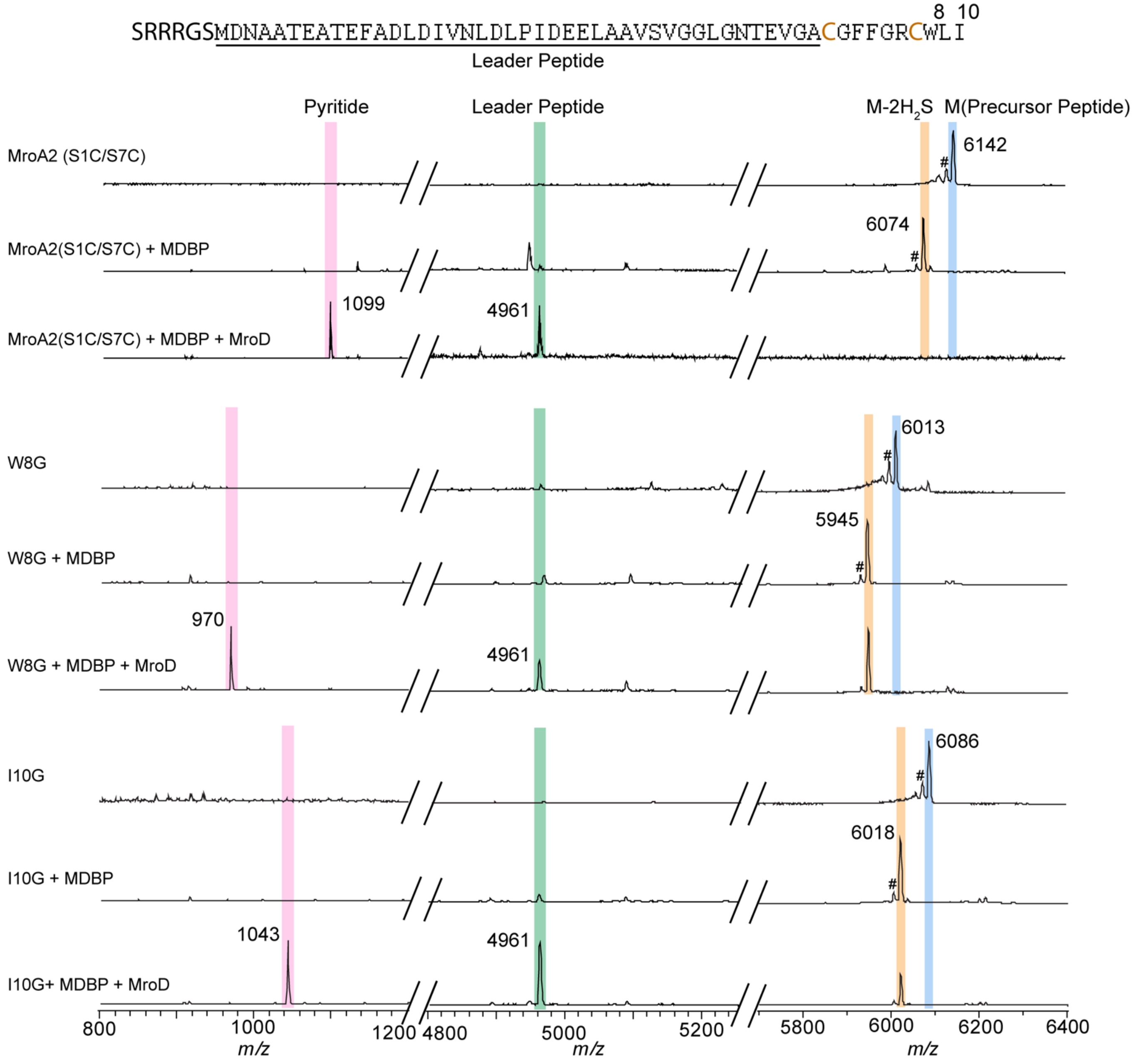

Figure S23: MALDI-TOF-MS analysis of shown MroA2 multi-site variants 1-10 after MroB/C/D assay The peptide sequences corresponding to the variant numerical orders are provided in Table S6. The varied region is highlighted in blue in the precursor peptide sequence below. The sequence of each variant that replaces the FFGR sequence is indicated in each mass spectrum. All spectra were acquired using reflector positive mode MALDI-TOF-MS. Unless otherwise stated, all peaks are $[M+H]^+$. The precursor peptides were generated through *in vitro* translation (see Experimental Methods). The f in the precursor peptide sequence represents a formyl group, which results from formyl-methionine utilized in *in vitro* translation. The pyritide macrocycles, the ejected leader peptides, and the remaining didehydrated intermediates are annotated accordingly. The # mark in the mass spectra represents a -17 Da artifact resulting from deamination specific to reflector positive mode MALDI-TOF-MS.[13, 14] The x mark represents a +211 Da adduct caused by a DTT addition to Dha residues and carboxymethylation of the adduct.

f-MRRRGSMDNVVTEAAEFADLDIVDLDLAVDEELAALSVGGLGNTEVGA|SG**FFGR**SWLI

Leader peptide

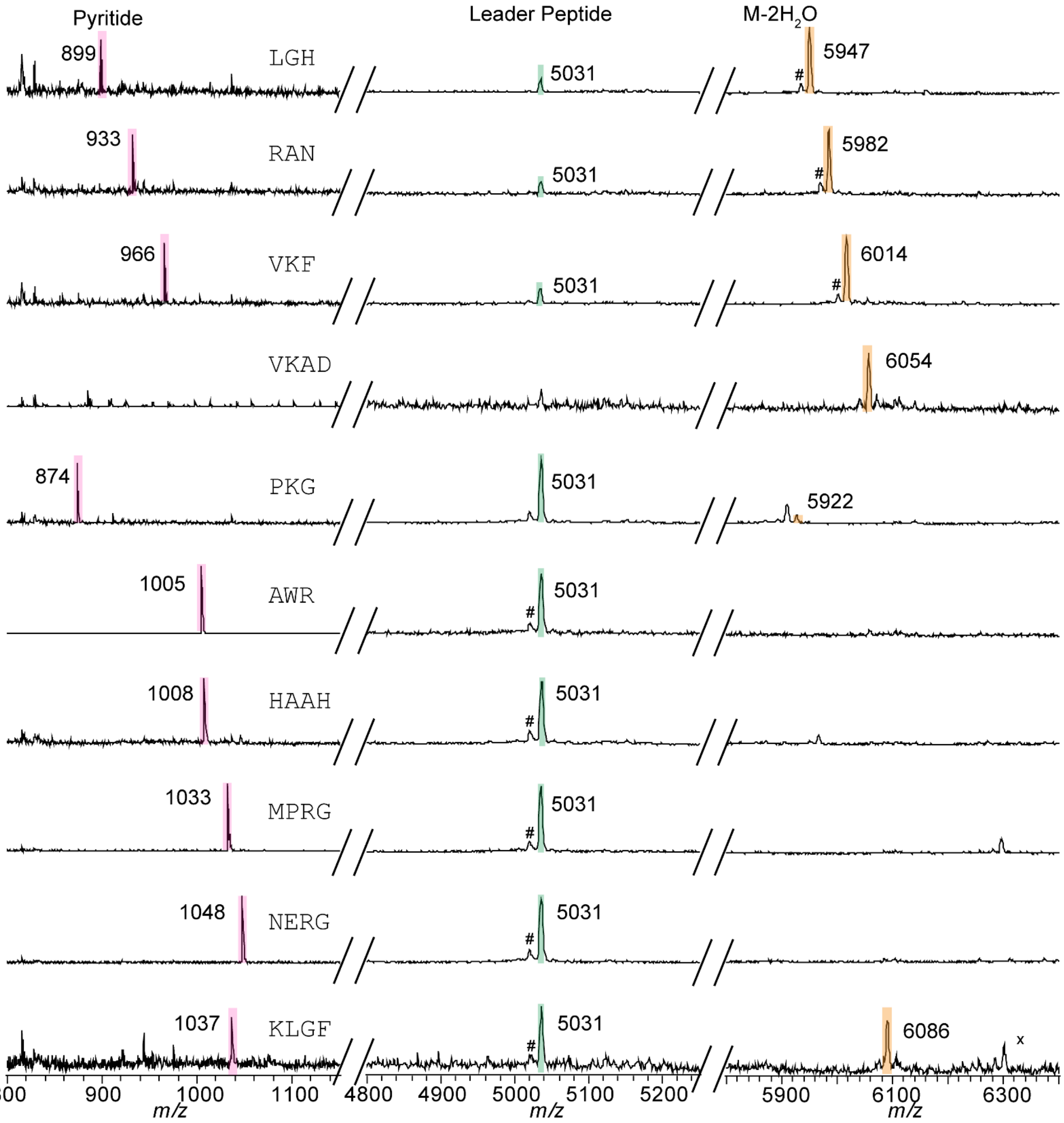

Figure S24: MALDI-TOF-MS analysis of shown MroA2 multi-site variants 11-19 after MroB/C/D assay. The peptide sequences corresponding to the variant numerical orders are provided in Table S6. The varied region is highlighted in blue in the precursor peptide sequence. The sequence of each variant that replaces the FFGR sequence is indicated in each mass spectrum. All results were acquired using reflector positive mode MALDI-TOF-MS. Unless otherwise stated, all peaks are $[M+H]^+$. The precursor peptides were generated through *in vitro* translation (see Experimental Methods). The f in the precursor peptide sequence represents a formyl group, which results from formyl-methionine utilized in *in vitro* translation. The pyritide macrocycles, the ejected leader peptides, and the remaining didehydrated intermediates are annotated accordingly. The # marks in the mass spectra represent a -17 Da artifact resulting from deamination specific to reflector positive mode in MALDI-TOF-MS.[13, 14]

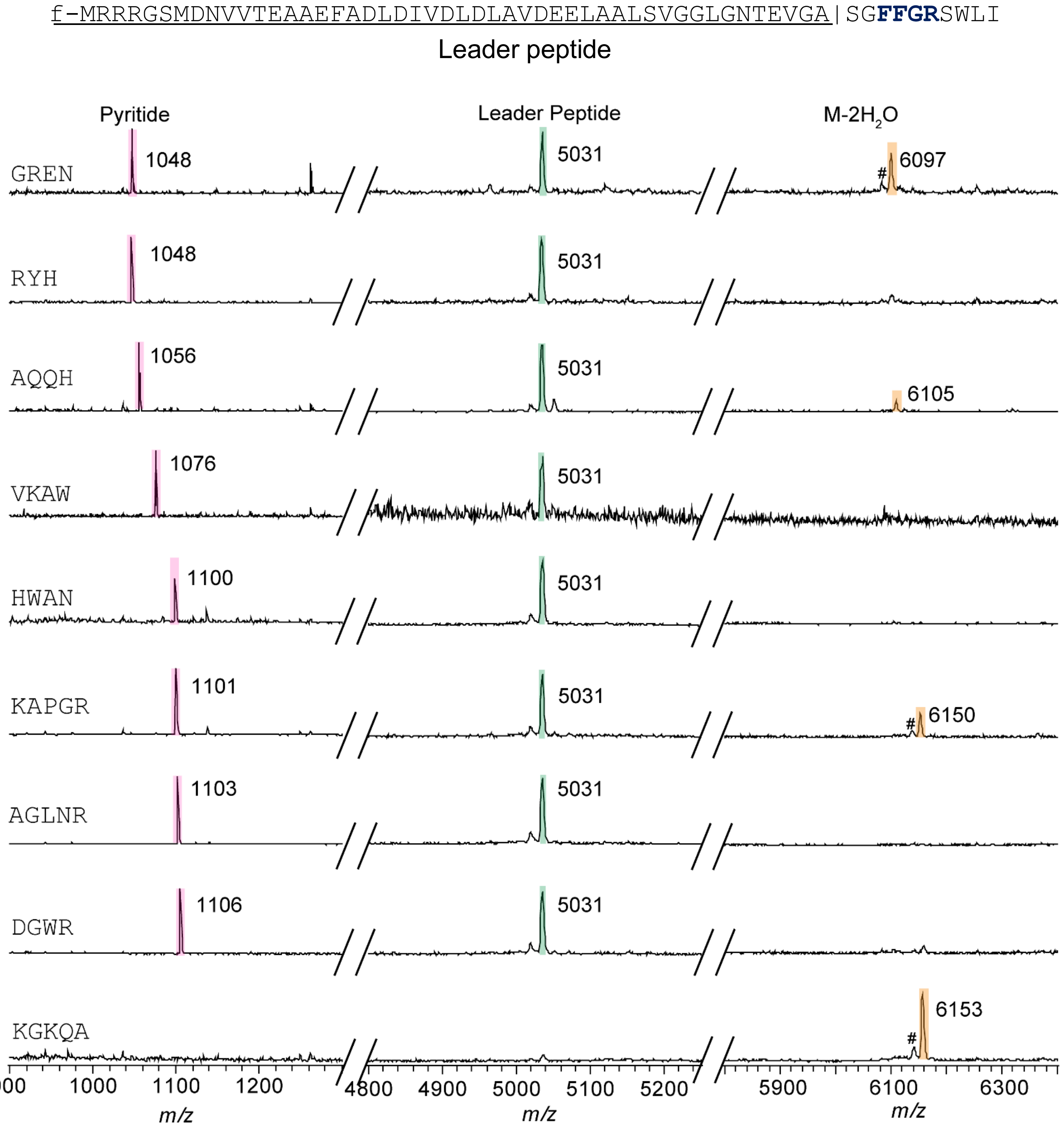

Figure S25: MALDI-TOF-MS analysis of shown MroA2 multi-site variants 20-29 after MroB/C/D assay. The peptide sequences corresponding to the variant numerical orders are provided in Table S6. The varied region is highlighted in blue in the precursor peptide sequence. The sequence of each variant that replaces the FFGR sequence is indicated in each mass spectrum. All results were acquired using reflector positive mode MALDI-TOF-MS. Unless otherwise stated, all peaks are $[M+H]^+$. The precursor peptides were generated through *in vitro* translation (see Experimental Methods). The f in the precursor peptide sequence represents a formyl group, which results from formyl-methionine utilized in *in vitro* translation. The pyritide macrocycles, the ejected leader peptides, and the remaining didehydrated and monodehydrated intermediates are annotated and colored accordingly. The * and ** marks in the mass spectra represent +16 Da and +32 Da species caused by oxidation during sample preparation.[15]

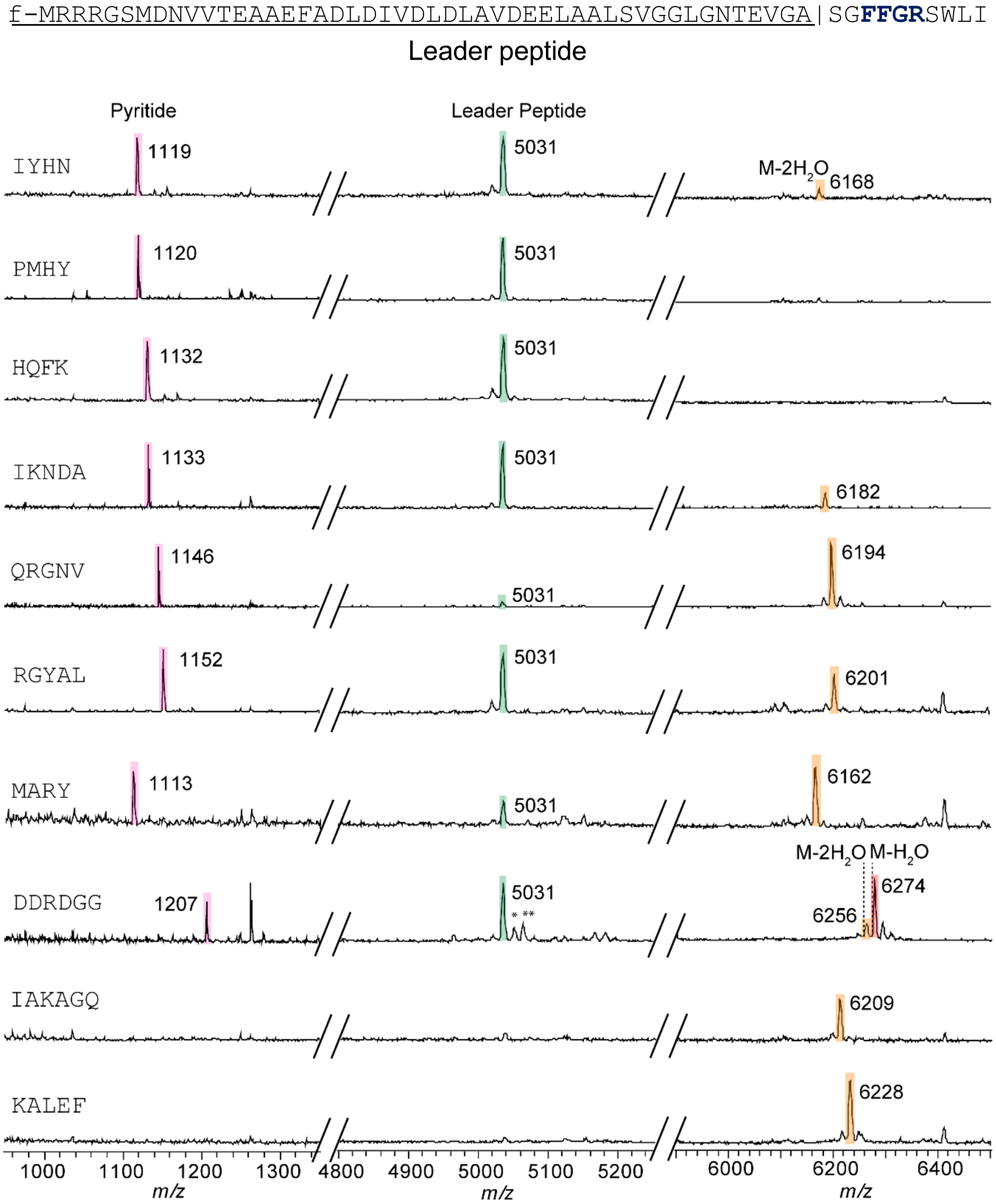

Figure S26: MALDI-TOF-MS analysis of shown MroA2 multi-site variants 30-39 after MroB/C/D assay. The peptide sequences corresponding to the variant numerical orders are provided in Table S6. The varied region is highlighted in blue in the precursor peptide sequence. The sequence of each variant that replaces the FFGR sequence is indicated in each mass spectrum. All results were acquired using reflector positive mode MALDI-TOF-MS. Unless otherwise stated, all peaks are $[M+H]^+$. The precursor peptides were generated through *in vitro* translation (see Experimental Methods). The f in the precursor peptide sequence represents a formyl group, which results from formyl-methionine utilized in *in vitro* translation. The pyritide macrocycles, the ejected leader peptides, and the remaining didehydrated intermediates are annotated accordingly.

f-MRRRGSMDNVVTEAAEFADLDIVDLDLAVDEELAALSVGGLGNTEVGA|SG**FFGR**SWLI

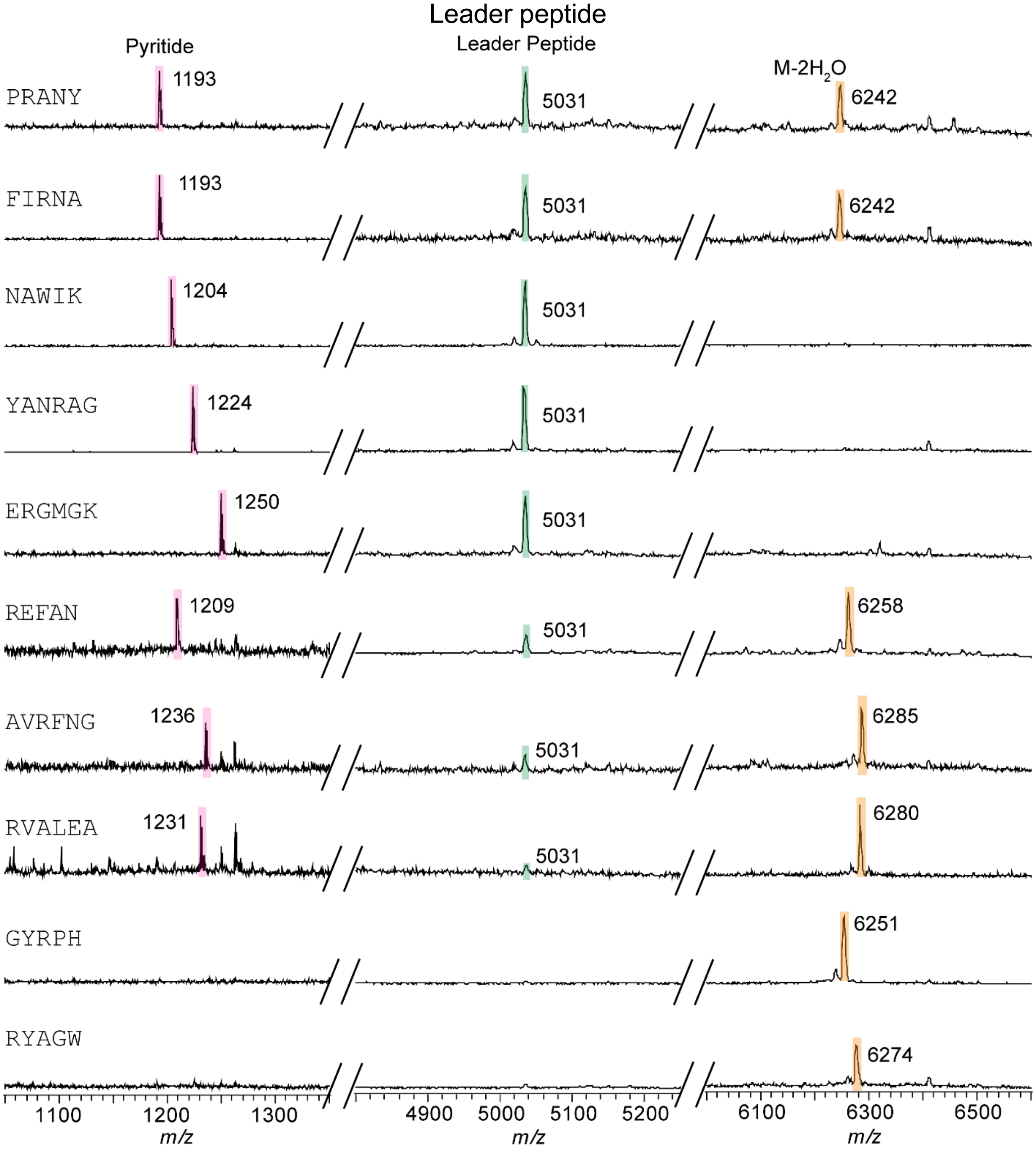

Figure S27: MALDI-TOF-MS analysis of shown MroA2 multi-site variants 40-47 after MroB/C/D assay. The peptide sequences corresponding to the variant numerical orders are provided in Table S6. The varied region is highlighted in blue in the precursor peptide sequence. The sequence of each variant that replaced the FFGR sequence is indicated in each mass spectrum. All spectra were acquired using reflector positive mode MALDI-TOF-MS. Unless otherwise stated, all peaks are $[M+H]^+$. The precursor peptides were generated through *in vitro* translation (see Experimental Methods). The f in the precursor peptide sequence represents formyl, which results from formyl-methionine utilized in *in vitro* translation. The pyritide macrocycles, the ejected leader peptides, and the remaining didehydrated intermediates are annotated accordingly.

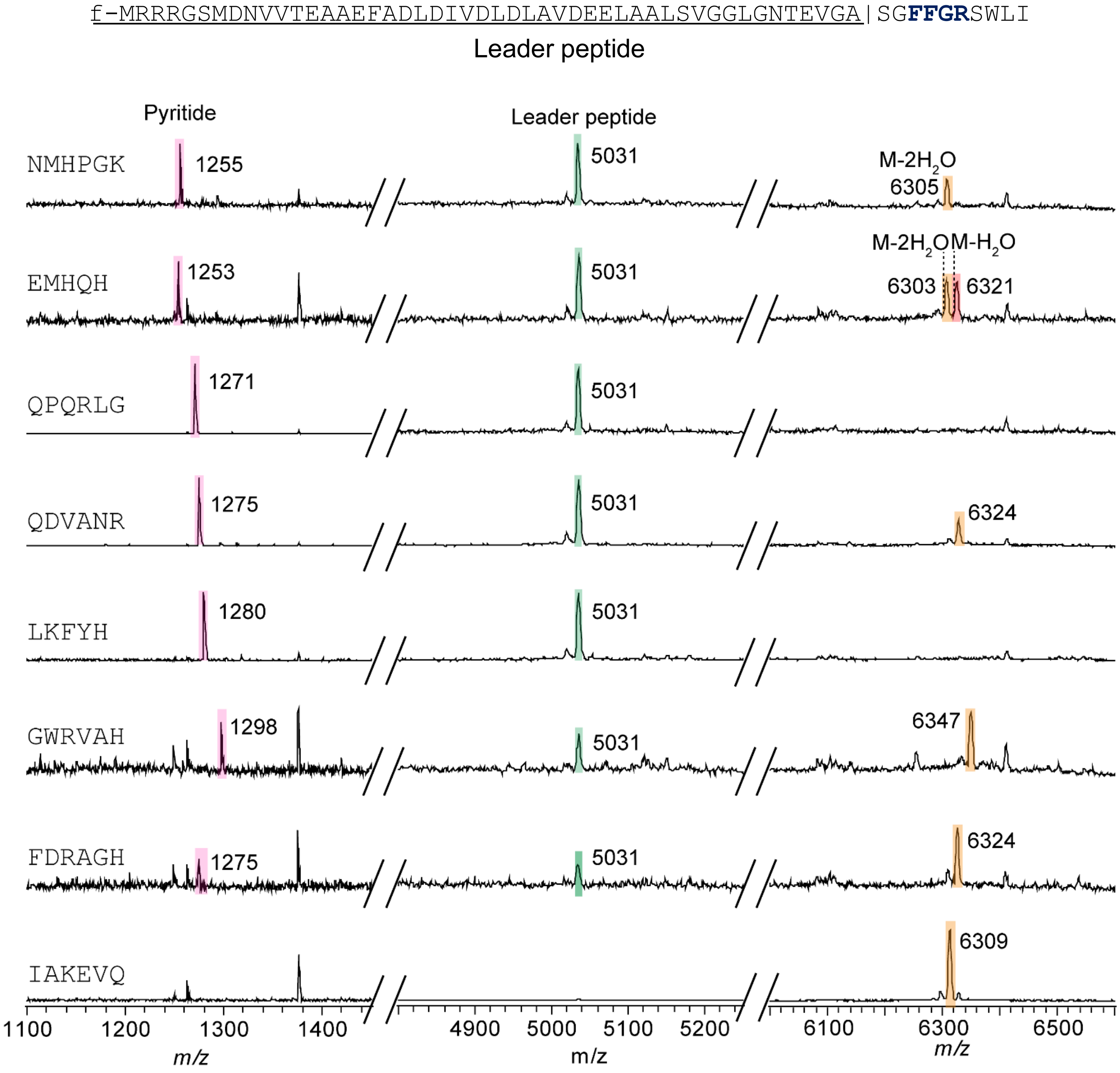

Figure S28: MALDI-TOF-MS analysis of shown MroA2 multi-site variants 48-56 after MroB/C/D assay. The peptide sequences corresponding to the variant numerical orders are provided in Table S6. The varied region is highlighted in blue in the precursor peptide sequence. The sequence of each variant that replaced the FFGR sequence is indicated in each mass spectrum. All spectra were acquired using reflector positive mode MALDI-TOF-MS. Unless otherwise stated, all peaks are $[M+H]^+$. The precursor peptides were generated through *in vitro* translation (see Experimental Methods). The f in the precursor peptide sequence represents a formyl group, which results from formyl-methionine utilized in *in vitro* translation. The pyritide macrocycles, the ejected leader peptides, and the remaining didehydrated intermediates are annotated accordingly.

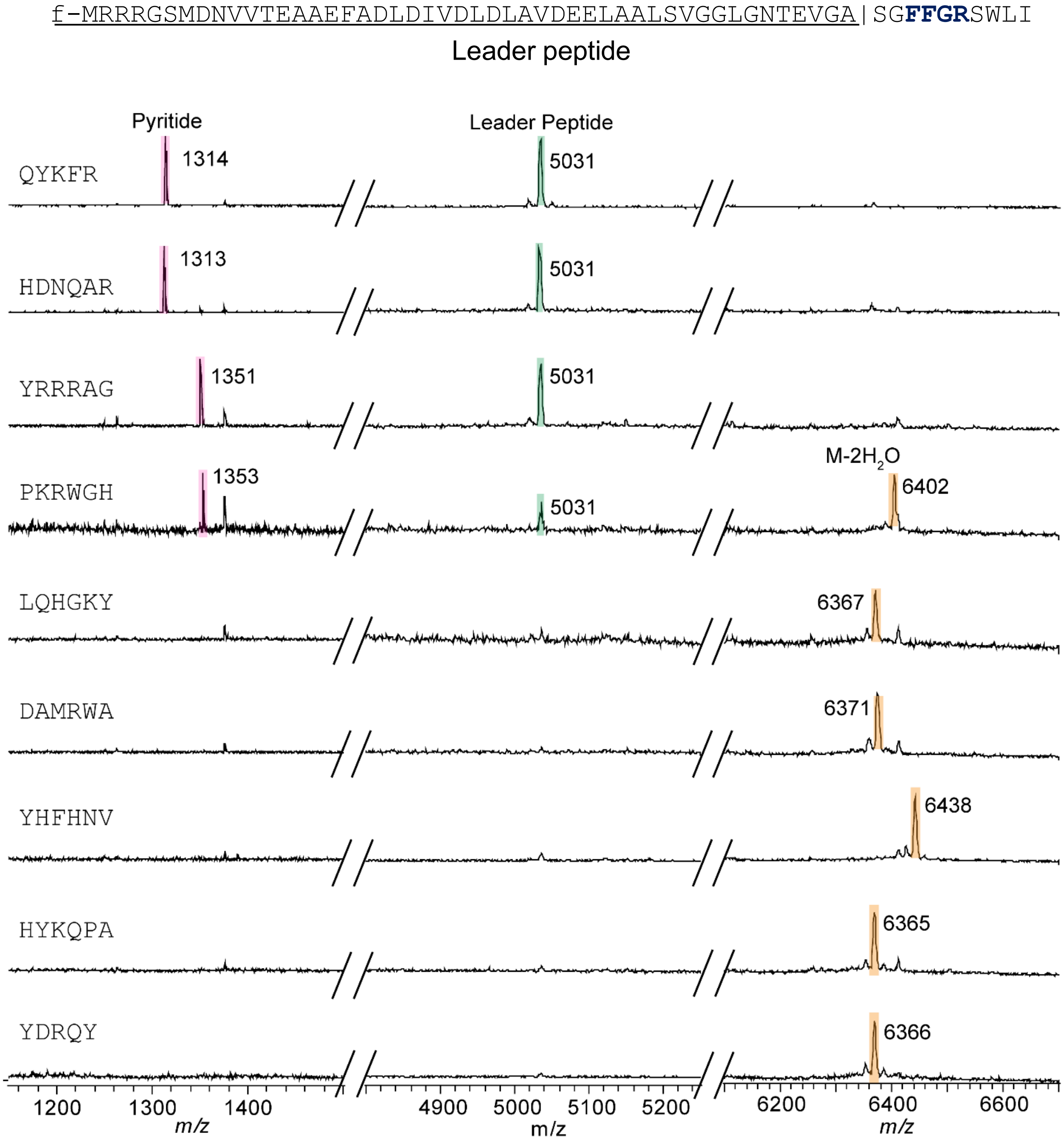

Table S6: Summary of MroB/C/D activity on MroA2 multi-site variants. The varied region is highlighted in blue in the precursor peptide sequence. MroD relative activity was qualitatively estimated by comparing the intensity of leader peptide and remaining didehydrated intermediates. +++ indicates enzyme activity roughly equal to wild-type MroA2 (major species are ejected leader peptides and produced macrocycles; insignificant amount of remaining intermediates are observed); ++ indicates modestly reduced enzyme activity (both significant amount of ejected leader peptides and intermediates are observed); + indicates severely reduced enzyme activity (a high-intensity peak of remaining didehydrated intermediate, a low-intensity peak of ejected leader peptide, and observable produced macrocycles); - indicates no detectable enzyme activity (no macrocycles detected).

f-MRRRGSMDNVVTEAAEFADLDIVDLDLAVDEELAALSVGGLGNTEVGA|SG**FFGR**SWLI

Leader peptide

Number	Core peptide sequence	Sequence of the varied region	Ring size	Dha formation	Relative MroD conversion
MroA2	SGFFGRSWLI	FFGR	17	2 Dha	+++
1	SGLGHSWLI	LGH	14	2 Dha	+
2	SGRANSWLI	RAN	14	2 Dha	+
3	SGVKFSWLI	VKF	14	2 Dha	+
4	SGVKADSWLI	VKAD	17	2 Dha	-
5	SGPKGSWLI	PKG	14	2 Dha	+++
6	SGAWRSWLI	AWR	14	2 Dha	+++
7	SGHAAHSWLI	HAAH	17	2 Dha	+++
8	SGMPRGSWLI	MPRG	17	2 Dha	+++
9	SGNERGSWLI	NERG	17	2 Dha	+++
10	SGKLGFSWLI	KLGF	17	2 Dha	+
11	SGGRENSWLI	GREN	17	2 Dha	++
12	SGRYHSWLI	RYH	14	2 Dha	+++
13	SGAQQHSWLI	AQQH	17	2 Dha	+++
14	SGVKAWSWLI	VKAW	17	2 Dha	+++
15	SGHWANSWLI	HWAN	17	2 Dha	+++
16	SGKAPGRSWLI	KAPGR	20	2 Dha	+++
17	SGAGLNRSWLI	AGLNR	20	2 Dha	+++
18	SGDGWRSWLI	DGWR	17	2 Dha	+++
19	SGKGKQASWLI	KGKQA	20	2 Dha	-
20	SGIYHNSWLI	IYHN	17	2 Dha	+++
21	SGPMHYSWLI	PMHY	17	2 Dha	+++
22	SGHQFKSWLI	HQFK	17	2 Dha	+++
23	SGIKNDASWLI	IKNDA	20	2 Dha	+++
24	SGQGRNVSWLI	QGRNV	20	2 Dha	+
25	SGRGYALSWLI	RGYAL	20	2 Dha	++
26	SGMARYSWLI	MARY	17	2 Dha	+
27	SGDDRDGGSWLI	DDRDGG	23	Mix of 1,2 Dha	+
28	SGIAKAGQSWLI	IAKAGQ	23	2 Dha	-

**29**	SGKALEFSWLI	KALEF	20	2 Dha	-
**30**	SGPRANYSWLI	PRANY	20	2 Dha	++
**31**	SGFIRNASWLI	FIRNA	20	2 Dha	++
**32**	SGNAWIKSWLI	NAWIK	20	2 Dha	+++
**33**	SGYANRAGSWLI	YANRAG	23	2 Dha	+++
**34**	SGERGMGKSWLI	ERGMGK	23	2 Dha	+++
**35**	SGREFANSWLI	REFAN	20	2 Dha	+
**36**	SGAVRFNGSWLI	AVRFNG	23	2 Dha	+
**37**	SGRVALEASWLI	RVALEA	23	2 Dha	+
**38**	SGGYRPHSWLI	GYRPH	20	2 Dha	-
**39**	SGRYAGWSWLI	RYAGW	20	2 Dha	-
**40**	SGNMHPGKSWLI	NMHPGK	23	2 Dha	++
**41**	SGEMHQHSWLI	EMHQH	20	Mix of 1,2 Dha	+
**42**	SGQPQRLGSWLI	QPQRLG	23	2 Dha	+++
**43**	SGQDVANRSWLI	QDVANR	23	2 Dha	+++
**44**	SGLKFYHSWLI	LKFYH	20	2 Dha	+++
**45**	SGGWRVAHSWLI	GWRVAH	23	2 Dha	+
**46**	SGFDRAGHSWLI	FDRAGH	23	2 Dha	+
**47**	SGIAKEVQSWLI	IAKEVQ	23	2 Dha	-
**48**	SGQYKFRSWLI	QYKFR	20	2 Dha	+++
**49**	SGHDNQARSWLI	HDNQAR	23	2 Dha	+++
**50**	SGYRRRAGSWLI	YRRRAG	23	2 Dha	+++
**51**	SGPKRWGHSWLI	PKRWGH	23	2 Dha	+
**52**	SGLQHGKYSWLI	LQHGKY	23	2 Dha	-
**53**	SGDAMRWASWLI	DAMRWA	23	2 Dha	-
**54**	SGYHFHNVSWLI	YHFHNV	23	2 Dha	-
**55**	SGHYKPQASWLI	HYKPQA	23	2 Dha	-
**56**	SGYDRQYSWLI	YDRQY	20	2 Dha	-
**24**-V7R	SGQGRNRSWLI	QGRNR	20	2 Dha	+++
**29**-F7R	SGKALERSWLI	KALER	20	Mix of 1,2 Dha	++
**35**-N7R	SGREFARSWLI	REFAR	20	2 Dha	+++
**38**-H7R	SGGYRPRSWLI	GYRPR	20	2 Dha	+++
**39**-W7R	SGRYAGRSWLI	RYAGR	20	2 Dha	+++
**47**-Q8R	SGIAKEVRSWLI	IAKEVR	23	2 Dha	-
**52**-Y8R	SGLQHGKRSWLI	LQHGKR	23	2 Dha	-
**53**-A8R	SGDAMRWRSWLI	DAMRWR	23	2 Dha	-
**54**-V8R	SGYHFHNRSWLI	YHFHNR	23	2 Dha	-
**55**-A8R	SGHYKPQRSWLI	HYKPQR	23	2 Dha	-
**56**-Y7R	SGYDRQRSWLI	YDRQR	20	2 Dha	+++

Figure S29: LC-HR-ESI-MS/MS analysis of macrocyclized product of MroA2 multi-site variant 43. The product was generated through MroB/C/D assays with substrate synthesized in 15 μL scale *in vitro* translation. The number of the compound is based on Table S6. A table comparing observed and theoretical *m/z* values for fragments may be found in **Supplementary Dataset 2**.

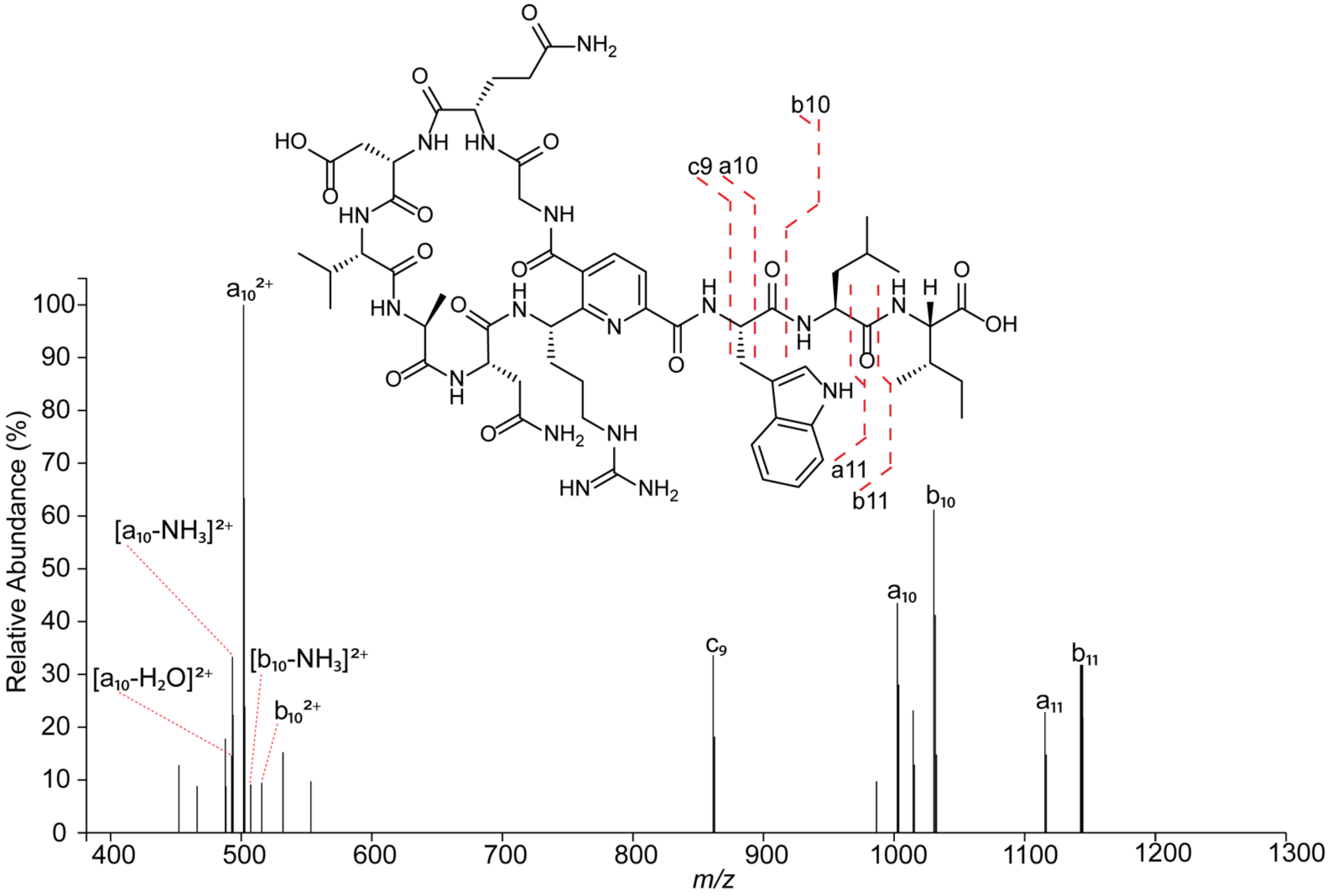

Figure S30: LC-HR-ESI-MS/MS analysis of macrocyclized product of MroA2 multi-site variant 22. The product was generated through MroB/C/D assays with substrate synthesized in 15 µL scale *in vitro* translation. The number of the compound is based on Table S6. A table comparing observed and theoretical *m/z* values for fragments may be found in **Supplementary Dataset 2**.

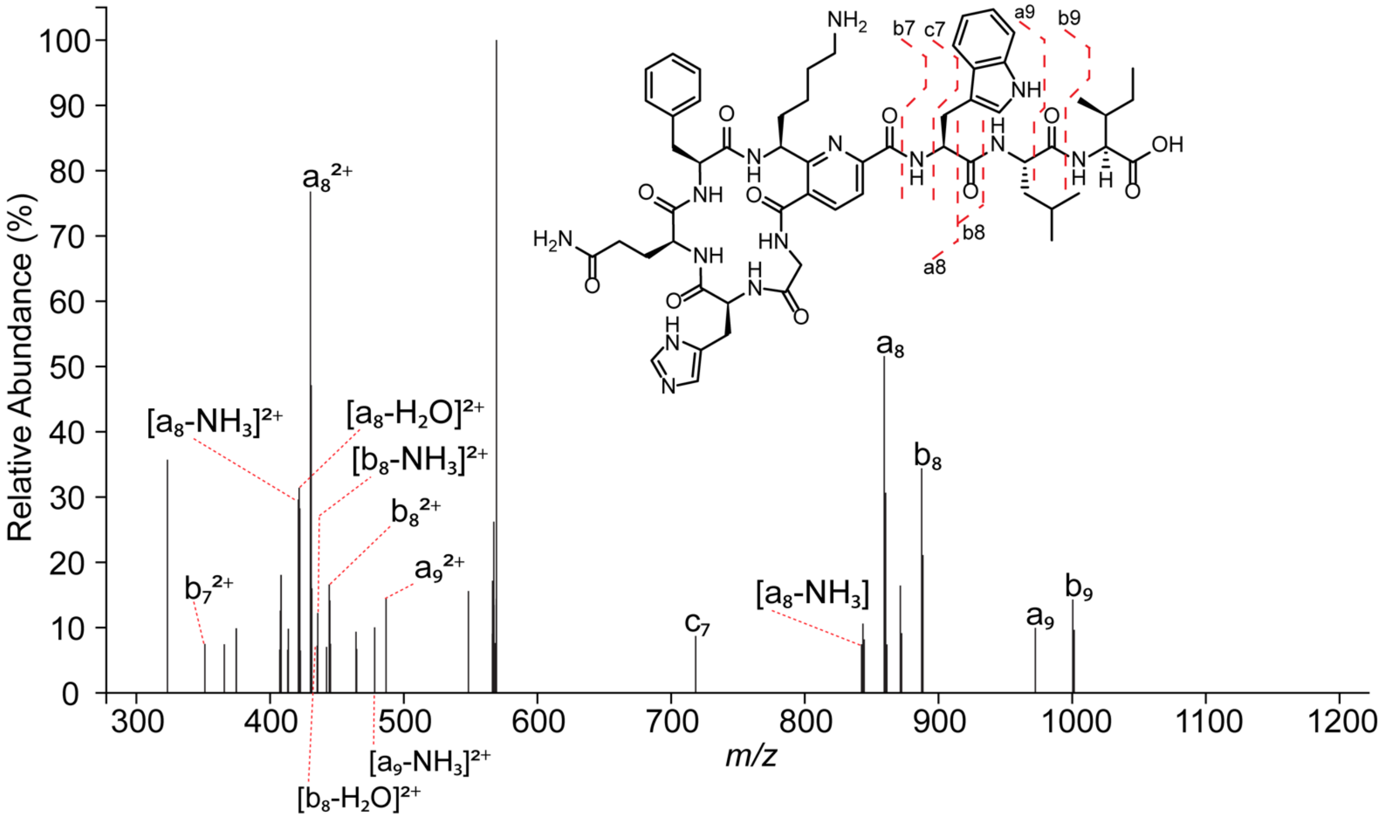

Figure S31: LC-HR-ESI-MS/MS analysis of macrocyclized product of MroA2 multi-site variant 6. The product was generated through MroB/C/D assays with substrate synthesized in 15 µL scale *in vitro* translation. The number of the compound is based on Table S6. A table comparing observed and theoretical *m/z* values for fragments may be found in **Supplementary Dataset 2**.

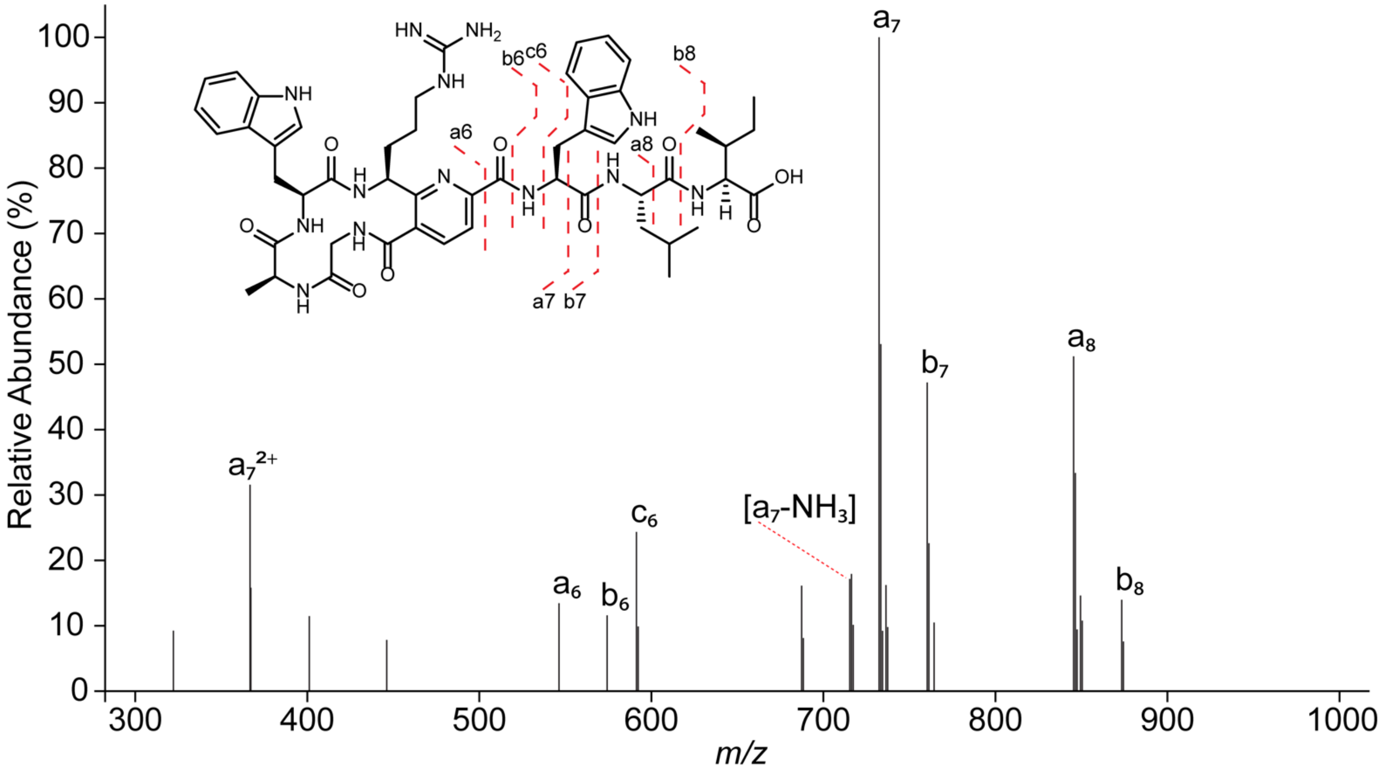

Figure S32: LC-HR-ESI-MS/MS analysis of macrocyclized product of MroA2 multi-site variant 17. The product was generated through MroB/C/D assays with substrate synthesized in 15 µL scale *in vitro* translation. The number of the compound is based on Table S6. A table comparing observed and theoretical *m/z* values for fragments may be found in **Supplementary Dataset 2**.

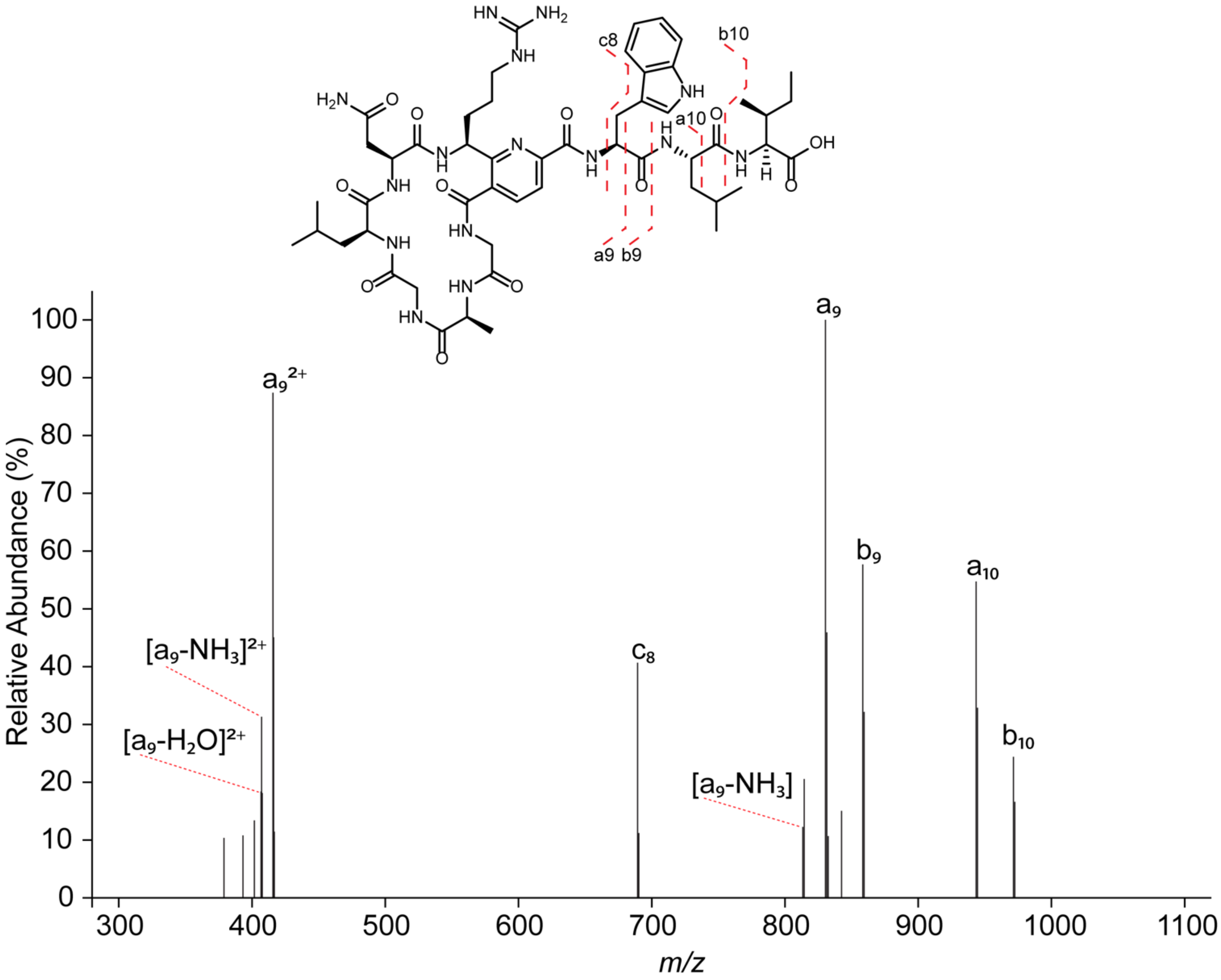

Figure S33: LC-HR-ESI-MS/MS analysis of macrocyclized product of MroA2 multi-site variant 15. The product was generated through MroB/C/D assays with substrate synthesized in 15 μL scale *in vitro* translation. The number of the compound is based on Table S6. A table comparing observed and theoretical *m/z* values for fragments may be found in **Supplementary Dataset 2**.

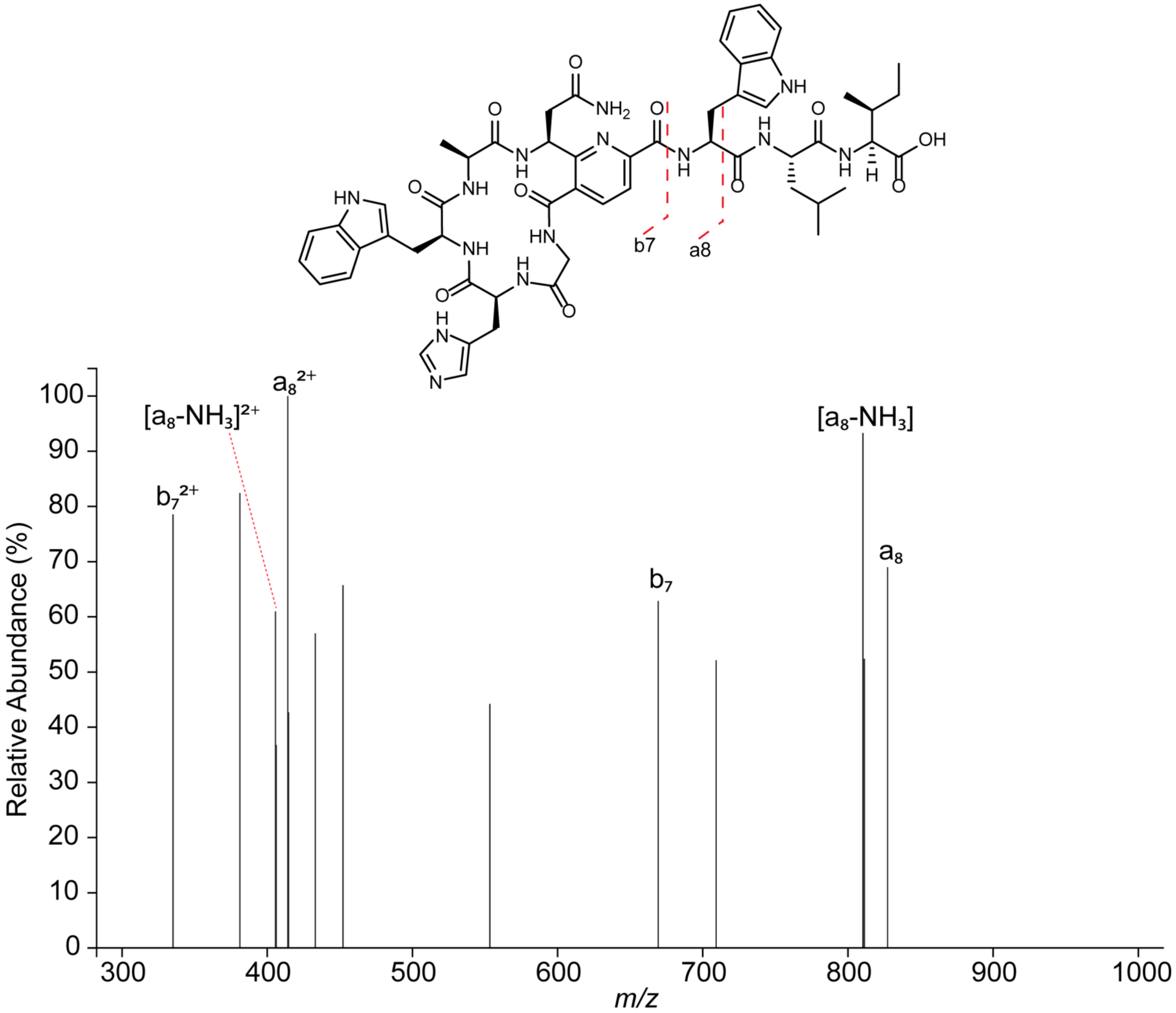

Figure S34: LC-HR-ESI-MS/MS analysis of macrocyclized product of MroA2 multi-site variant 44. The product was generated through MroB/C/D assays with substrate synthesized in 15 μL scale *in vitro* translation. The number of the compound is based on Table S6. A table comparing observed and theoretical *m/z* values for fragments may be found in **Supplementary Dataset 2**.

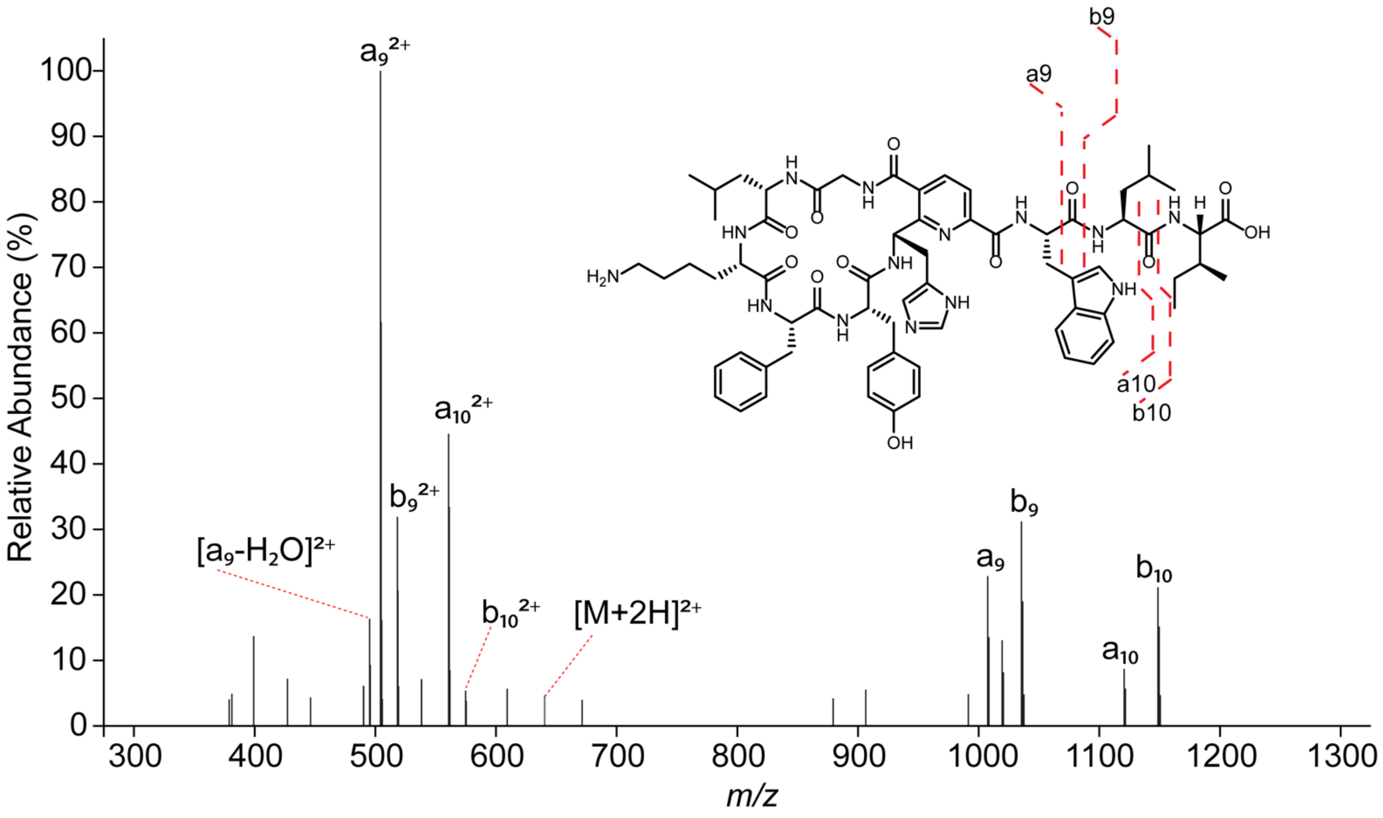

Figure S35: LC-HR-ESI-MS/MS analysis of macrocyclized product of MroA2 multi-site variant 42. The product was generated through MroB/C/D assays with substrate synthesized in 15 µL scale *in vitro* translation. The number of the compound is based on Table S6. A table comparing observed and theoretical *m/z* values for fragments may be found in **Supplementary Dataset 2**.

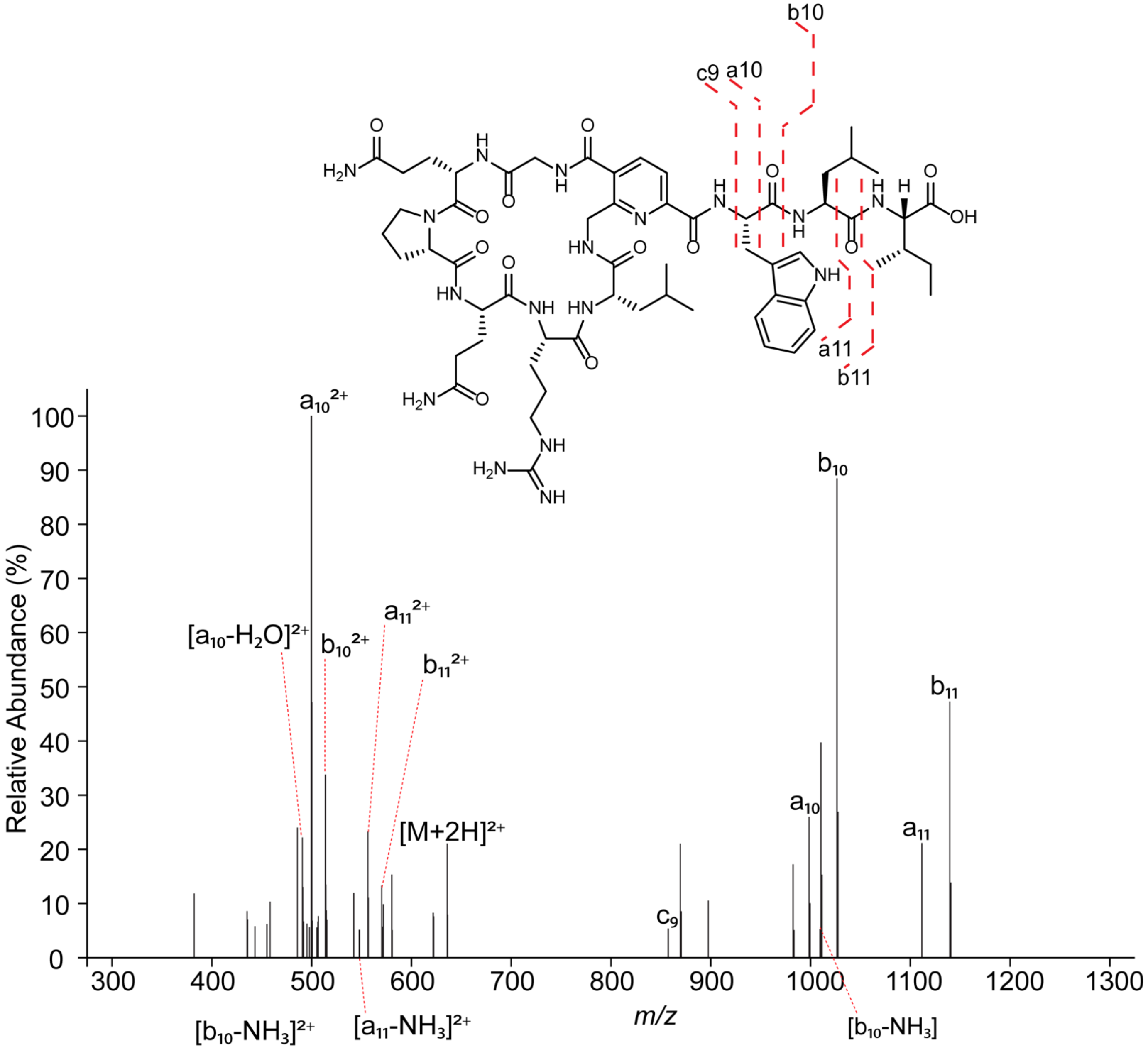

Figure S36: LC-HR-ESI-MS/MS analysis of macrocyclized product of MroA2 multi-site variant 7. The product was generated through MroB/C/D assays with substrate synthesized in 15 µL scale *in vitro* translation. The number of the compound is based on Table S6. A table comparing observed and theoretical *m/z* values for fragments may be found in **Supplementary Dataset 2**.

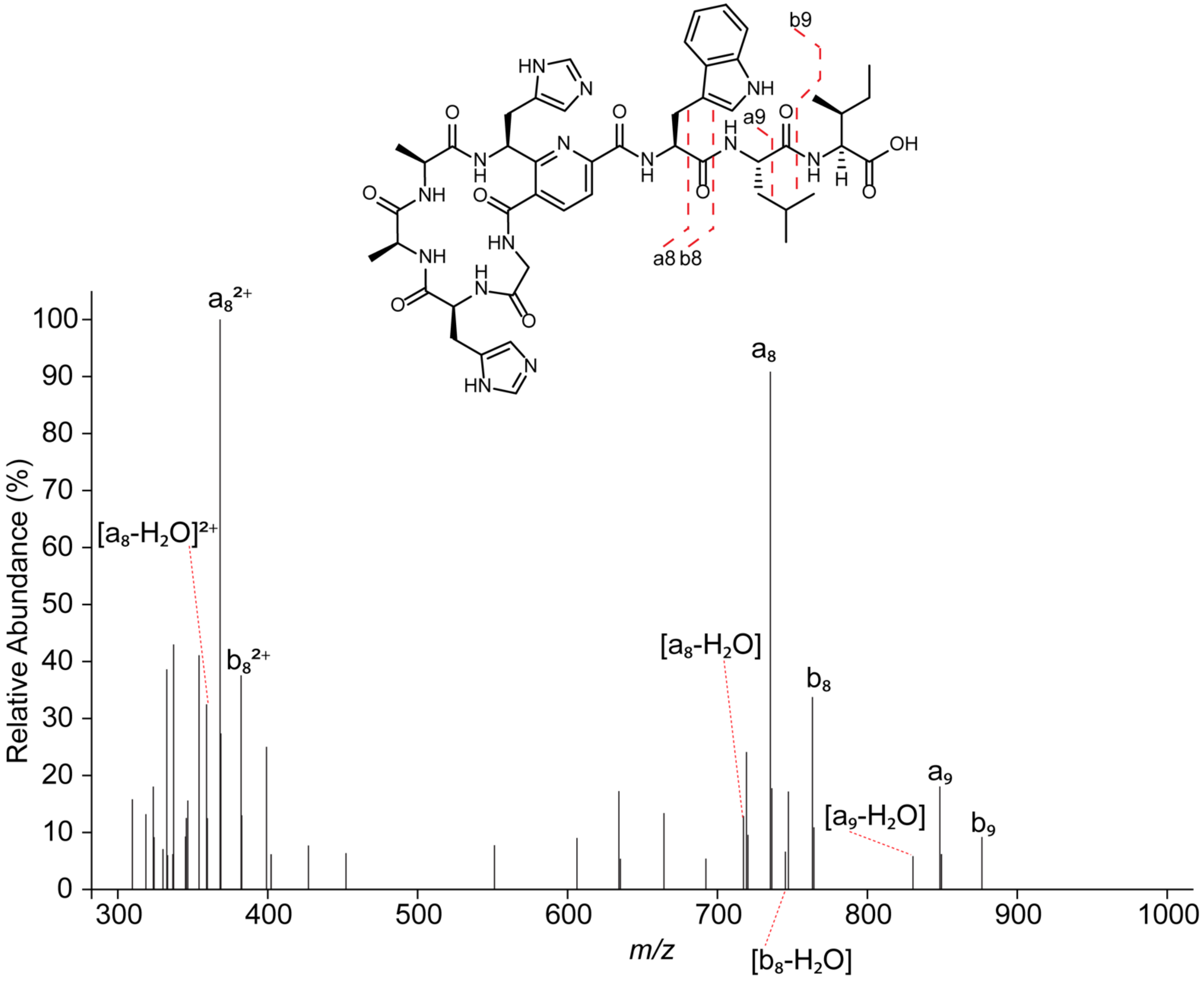

Figure S37: LC-HR-ESI-MS/MS analysis of macrocyclized product of MroA2 multi-site variant 12. The product was generated through MroB/C/D assays with substrate synthesized in 15 µL scale *in vitro* translation. The number of the compound is based on Table S6. A table comparing observed and theoretical *m/z* values for fragments may be found in **Supplementary Dataset 2**.

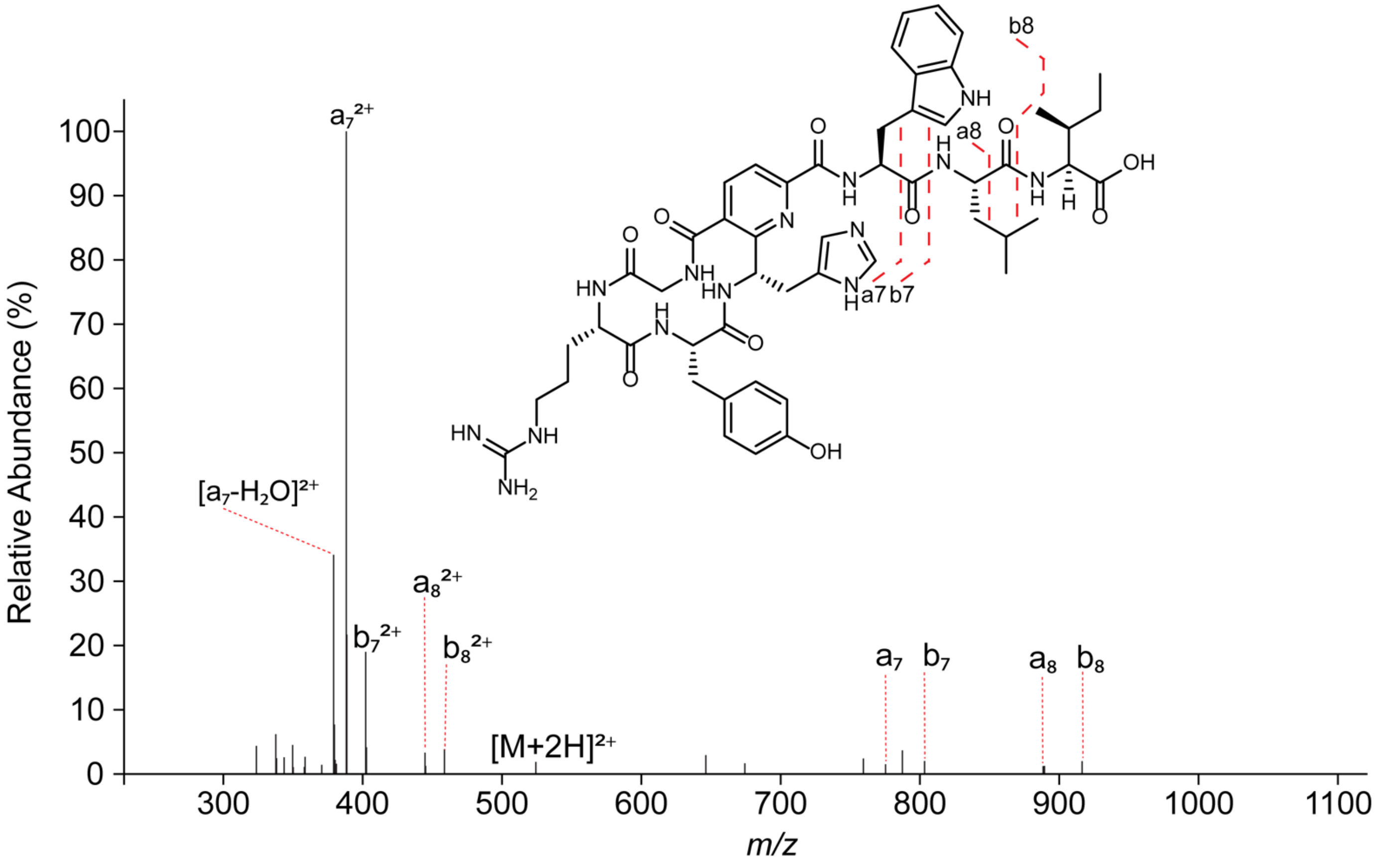

Figure S38: MALDI-TOF-MS analysis of Arg variants of uncyclized precursor peptides after MroB/C/D treatment. The sequences of original uncyclized precursors and their corresponding Arg variants are indicated in each mass spectrum. The sequence of MroA2 precursor peptide with the varied region highlighted in blue is shown. All spectra were acquired using reflector positive mode of MALDI-TOF-MS. Unless otherwise stated, all peaks are $[M+H]^+$. The precursor peptides were generated through in vitro translation (see Experimental Methods). The f in the precursor peptide sequence represents a formyl group, which results from formyl-methionine utilized in in vitro translation. The pyritide macrocycles, the ejected leader peptides, and the remaining didehydrated intermediates are annotated accordingly.

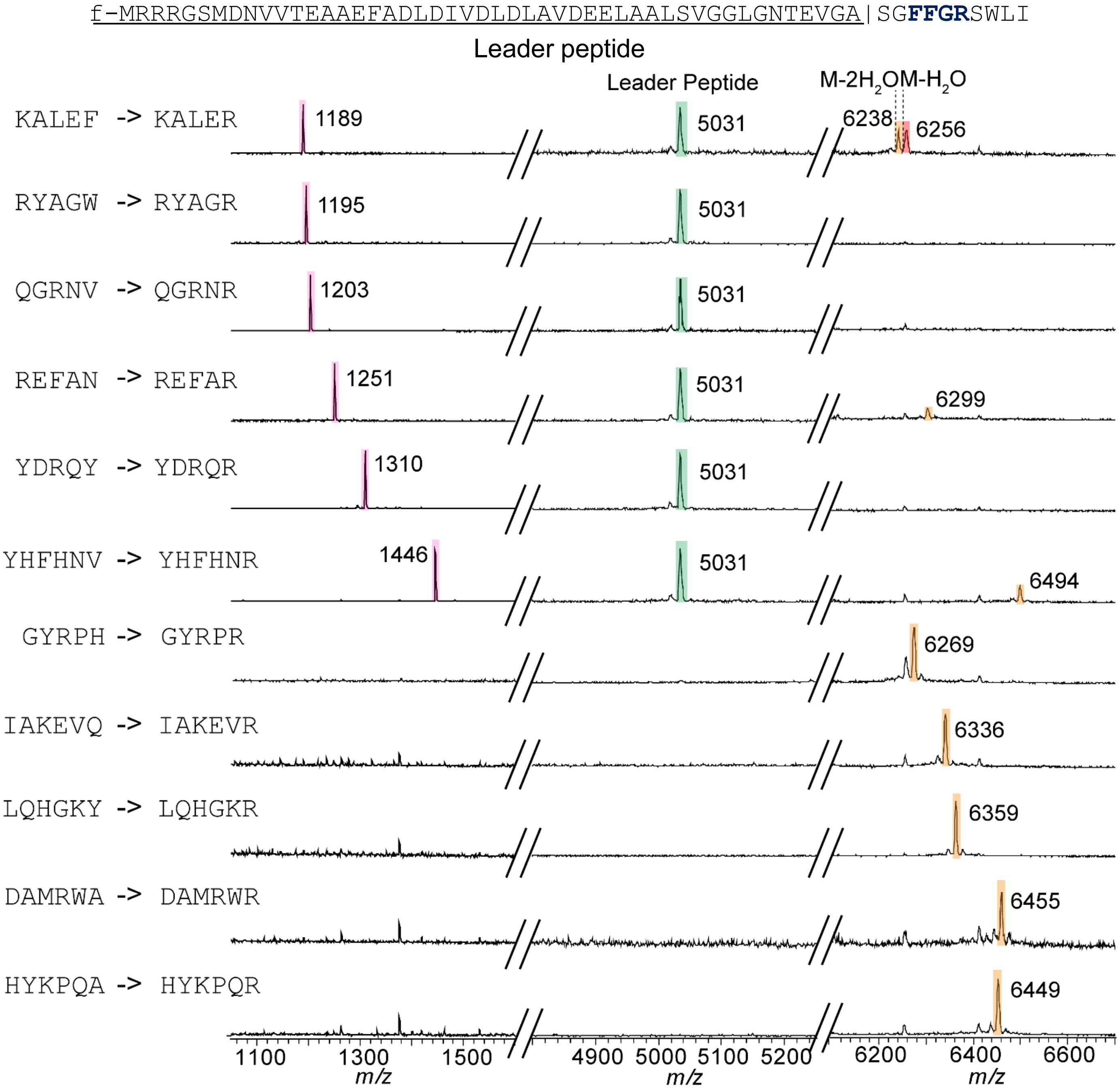

Table S7: Sequence alignment of pyritide precursor peptides. The sequences were identified from the GenBank database and aligned according to a previously reported bioinformatic protocol.[2] The table shows all identified pyritide precursor peptides found up to May 2022. The NCBI accessions of the precursor peptides are shown on the left. The box captures the amino acid residues removed to generate Δ12MroA1, which was utilized in fluorescence polarization experiments. The start codon of the last two precursor peptides were potentially misidentified by GenBank.

```
MroA1           ---------------------------MD---------NVVTEAAEFADLDIVDLDLAVDE-ELAALSVGGLGNTEVGASGW--LG-SWVI
MroA2           ---------------------------MD---------NAATEATEFADLDIVNLDLPIDE-ELAAVSVGGLGNTEVGASGF--FGRSWLI
WP_157518710.1  ---------------------------MATNEPF---------HLDLDSLDVMTVELPGED-LVKALGM-GLGNTEVGASGY--MRTSWVV
WP_175440426.1  ---------------------------ME---------DMAVEATEFADLDIVDLDLPIDE-ELAAVSIGGLGNTEVGASGF--WGRSWLI
WP_184982132.1  ---------------------------MATEEPL---------RLDLDTLDVMTVELPGED-LVKALGM-GLGNTEVGASGI-TGRTSWLI
WP_185845188.1  ---------------------------MDLHEE----------ALDLDALDVATVELPGSEVLVEAVAM-GLGNTEIGASGC-TSGKSWLI
WP_189161139.1  ---------------------------MATNEPL---------RLDLDSLDVMTVELPGED-LVQALGM-GLGNTEVGASGL-TGRTSWLI
WP_191909865.1  ---------------------------MAMHEE-----ALNLDALDLDSLDVATVELPGSDLLVEAVTM-GLGNTEVGASGAWTSRTSWLV
WP_204289226.1  ---------------------------MAMHEE-----ALNLDALDLDSLDVATVELPGSDVLVEAVTM-GLGNTEVGASGAWTSRTSWLV
WP_210159446.1  ---------------------------MAMHEEVLDLDALDLDSLDLDSLDVATVELPGSEVLVEAVTM-GLGNTEIGASGTWTSSKSWLV
WP_192785191.1  MDEIVDRAYGGDRHRYGPLRRVRMGVQMATNEPL---------RLDLDALDVMTVELPGED-LVKALGM-GLGNTEVGASGI--ARTSWLI
WP_157556456.1  MTIV-------------PEIARETGEQMANDEPL---------HLDLNSLDVTTVELPGED-LVEALGM-GLGNTEVGASGI--GRTSWLI
                                           *                 ::  **:  ::*. .:  : *: : ******:****      **::
```

Figure S39: MALDI-TOF-MS analysis of substrates containing Thr preceding the second Ser in the core peptide after MroB/C/D treatment. The varied region (X containing 3-6 amino acid residues) is highlighted in dark blue in the precursor peptide sequence. The sequence of each variant is indicated in each mass spectrum. All spectra were acquired using reflector positive mode or negative mode MALDI-TOF MS. Unless otherwise stated, all peaks are $[M+H]^+$. The precursor peptides were generated through *in vitro* translation (see Experimental Methods). The f in the precursor peptide sequence represents formyl, which results from formyl-methionine utilized in *in vitro* translation. The pyritide macrocycles, the ejected leader peptides, and the remaining didehydrated intermediates are annotated accordingly. Every precursor peptide sequence contains Trp-Leu-Ile as the three C-terminal residues, except for MroA1 G5T (Trp-Val-Ile).

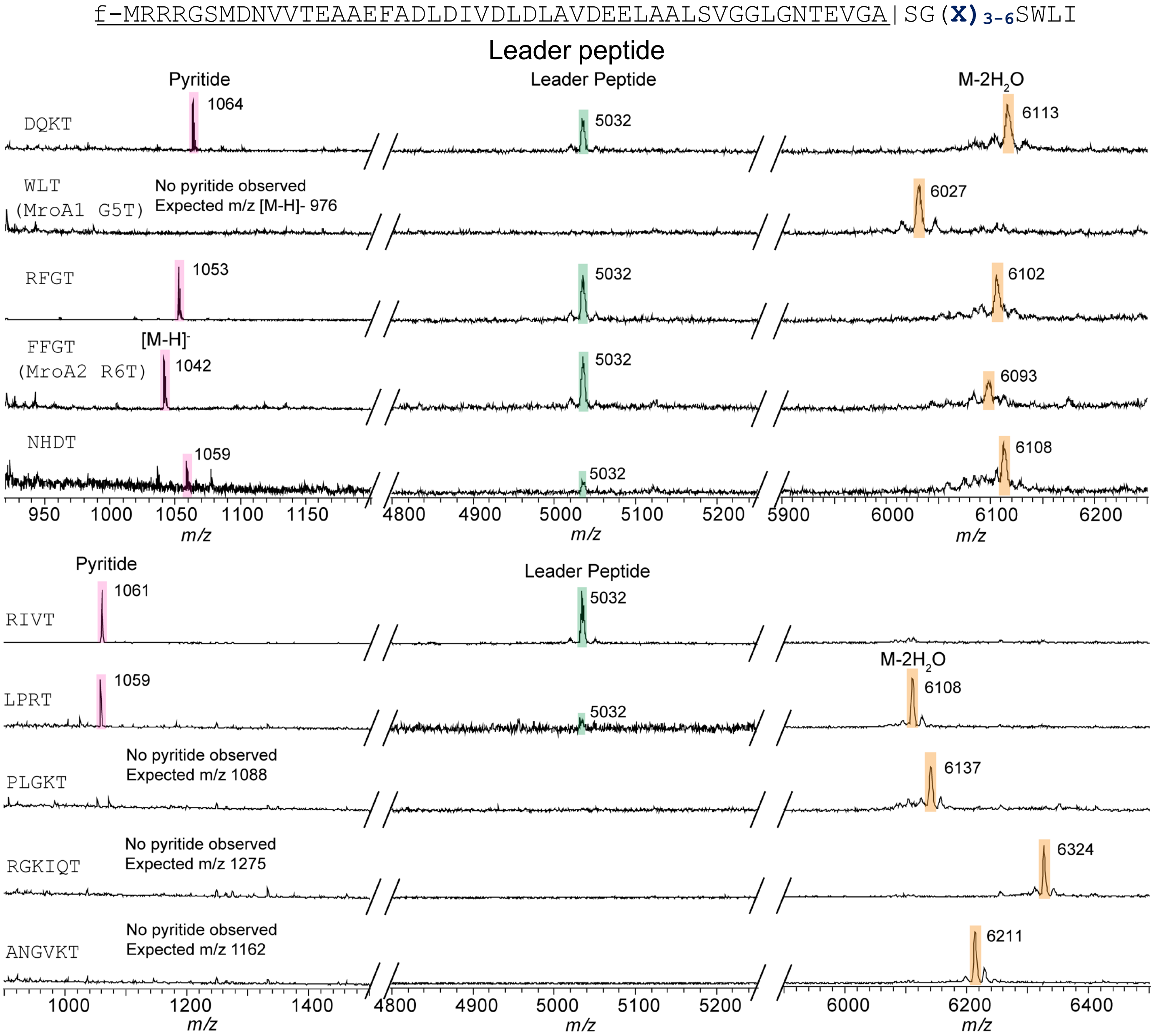

Figure S40: Substrates containing Thr preceding the second Ser likely are not dehydrated at this Thr by MroB/C. MALDI-TOF-MS analysis of Thr-containing substrates in Figure S39 having the Thr or the second Ser mutated to Ala. The core sequence of each substrates is indicated in each mass spectrum. All spectra were acquired using reflector positive mode MALDI-TOF-MS. Unless otherwise stated, all peaks are $[M+H]^+$. The precursor peptides were generated through *in vitro* translation (see Experimental Methods). The f in the precursor peptide sequence represents formyl, which results from formyl-methionine utilized in *in vitro* translation. The Ser which is dehydrated is bolded in purple in each mass spectrum. All substrates having Thr mutated to Ala had two dehydrations, while substrates having the second Ser mutated Ala had one only one dehydration. This result suggests that the substrates in Figure S39 had two Ser dehydrated, and substrates undergoing [4+2] cyclization form the pyridine moiety from two Dha rather than one Dha and one Dhb. Further evidence can be found in HR-ESI-LC-MS/MS data in Figure S41-S42.

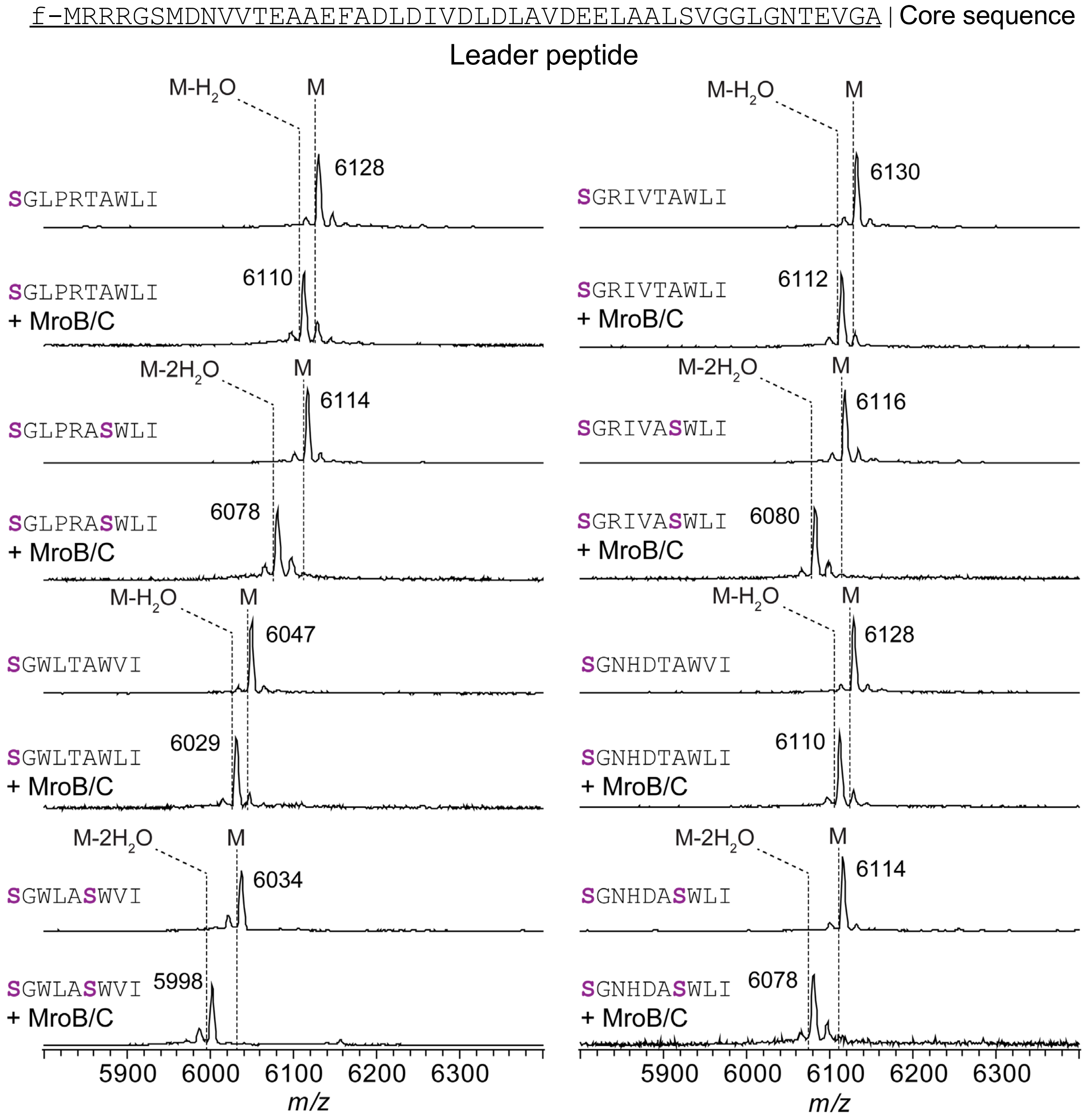

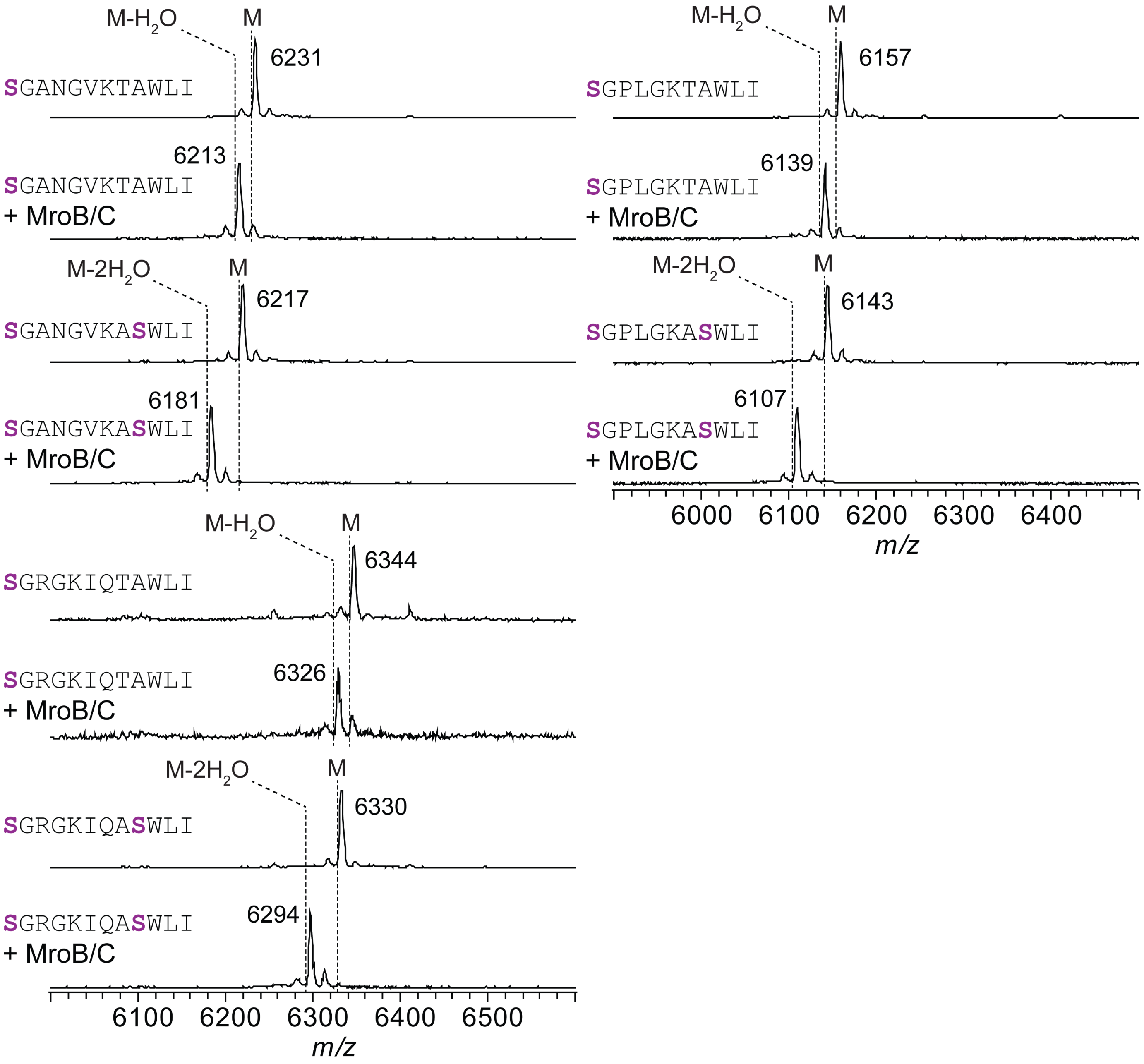

M-H_2O
M
6231
SGANGVKTAWLI
6213
SGANGVKTAWLI
+ MroB/C
M-2H_2O
M
6217
SGANGVKASWLI
6181
SGANGVKASWLI
+ MroB/C
M-H_2O
M
6157
SGPLGKTAWLI
6139
SGPLGKTAWLI
+ MroB/C
M-2H_2O
M
6143
SGPLGKASWLI
6107
SGPLGKASWLI
+ MroB/C
6000
6100
6200
6300
6400
m/z
M-H_2O
M
6344
SGRGKIQTAWLI
6326
SGRGKIQTAWLI
+ MroB/C
M-2H_2O
M
6330
SGRGKIQASWLI
6294
SGRGKIQASWLI
+ MroB/C
6100
6200
6300
6400
6500
m/z

Figure S41: LC-HR-ESI-MS/MS analysis of twice-dehydrated Thr-containing intermediates in Figure S39. The species were generated through MroB/C/D assays followed by GluC cleavage. The sequence of each peptide is shown in each panel. A table comparing observed and theoretical *m/z* values for fragments may be found in **Supplementary Dataset 2**.

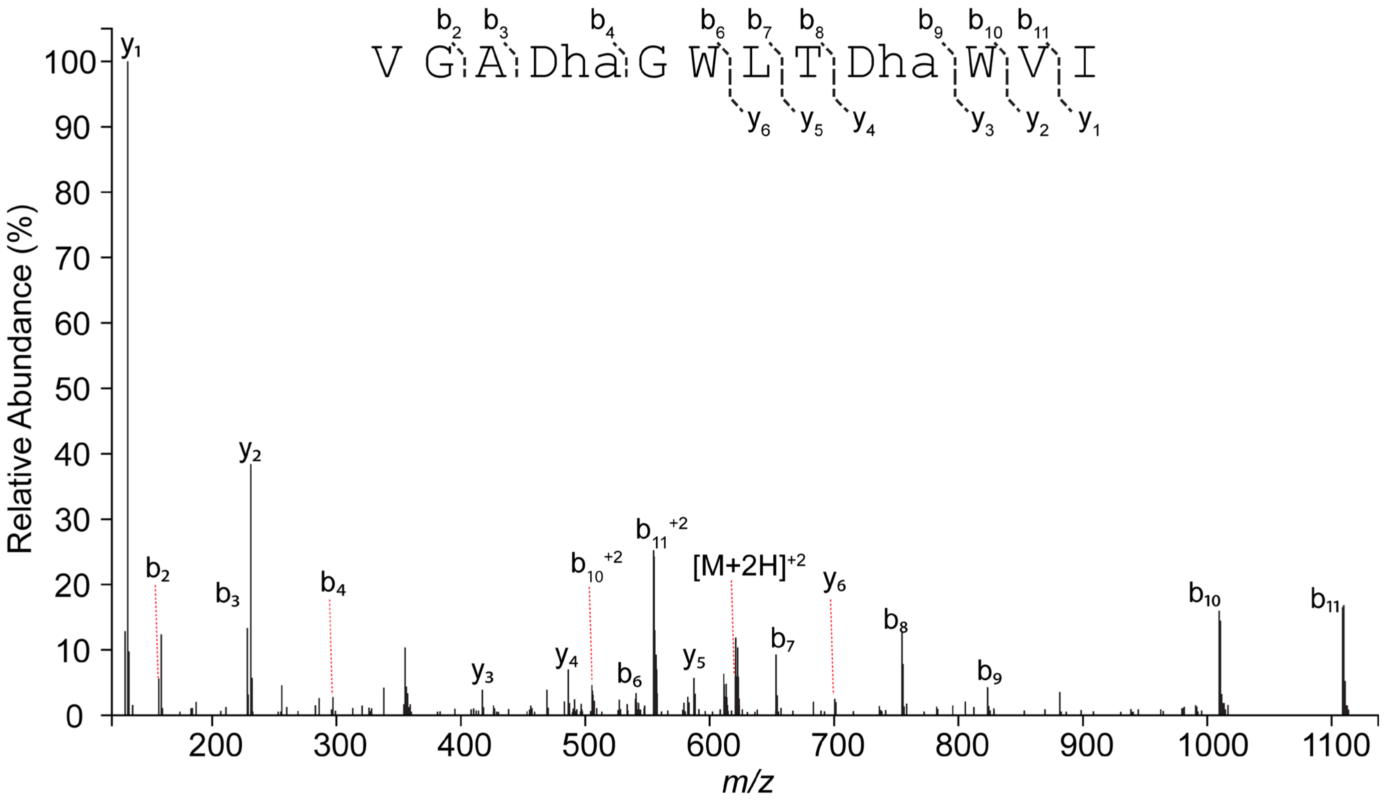

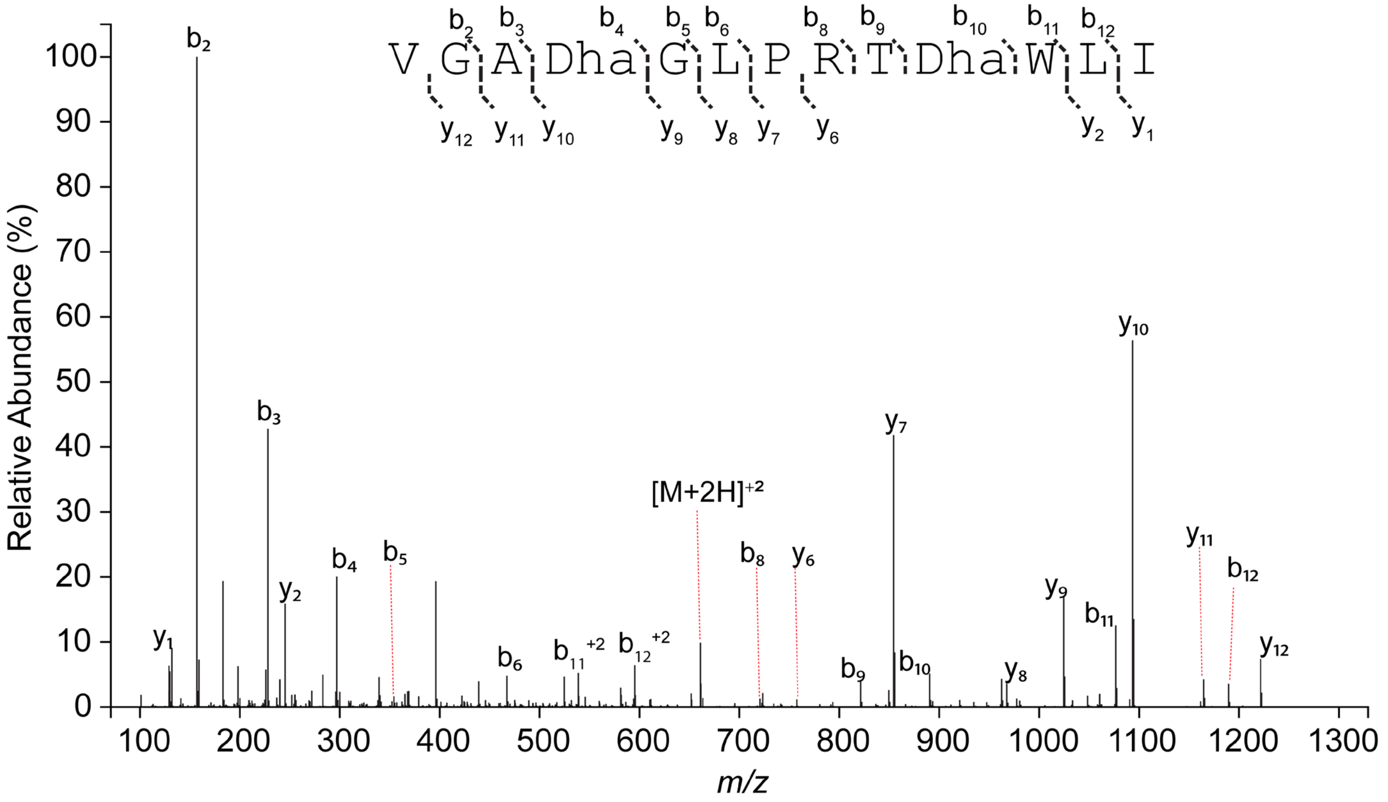

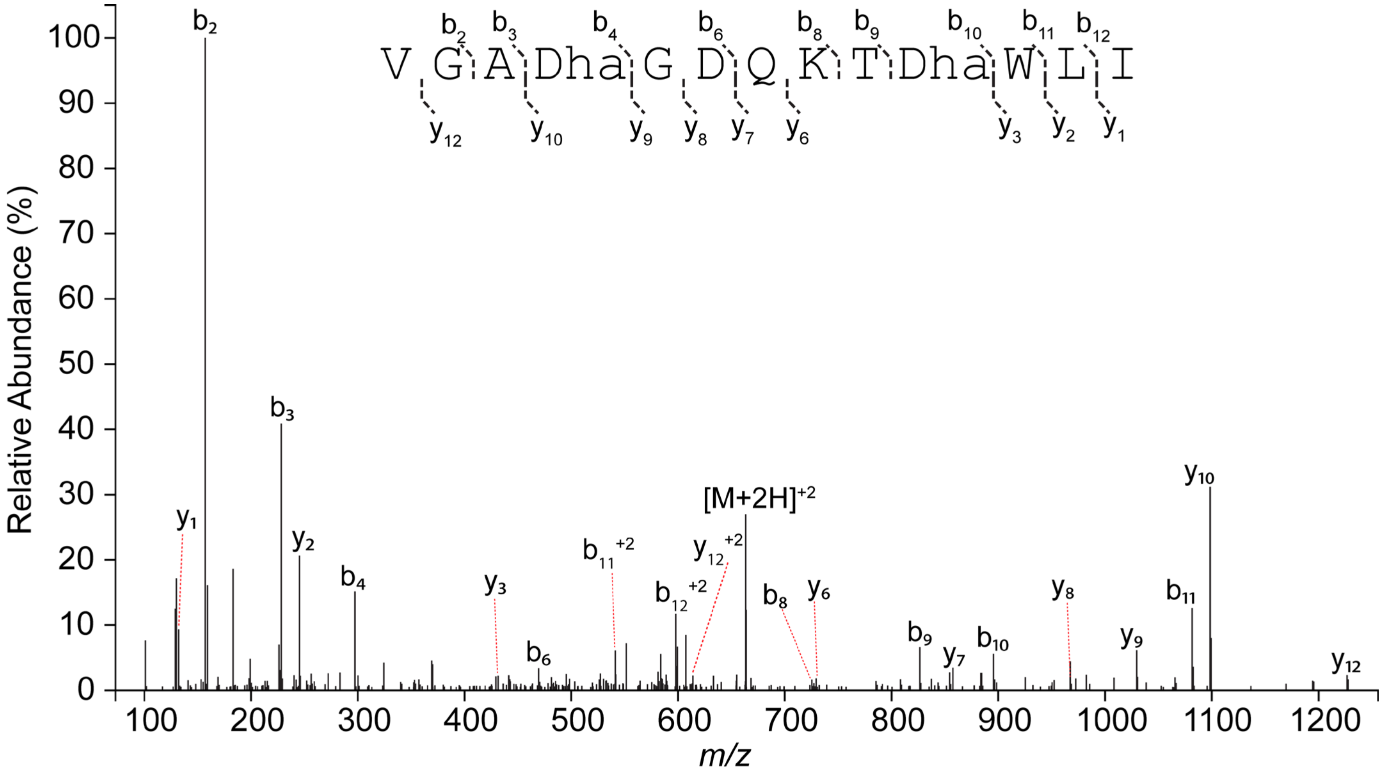

V G A Dha G D Q K T Dha W L I
Relative Abundance (%)
m/z
[M+2H]+2

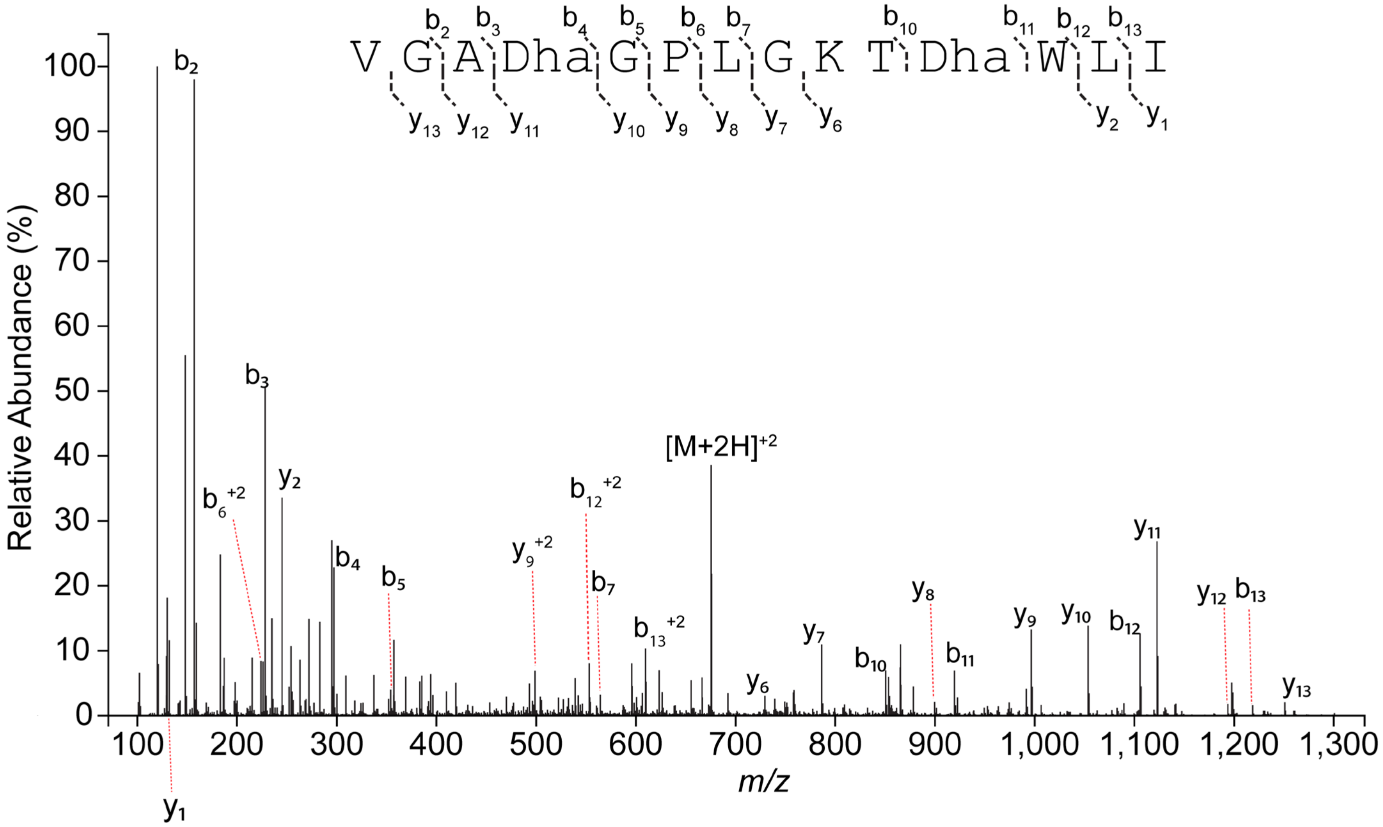

V G A Dha G P L G K T Dha W L I
Relative Abundance (%)
m/z
[M+2H]+2

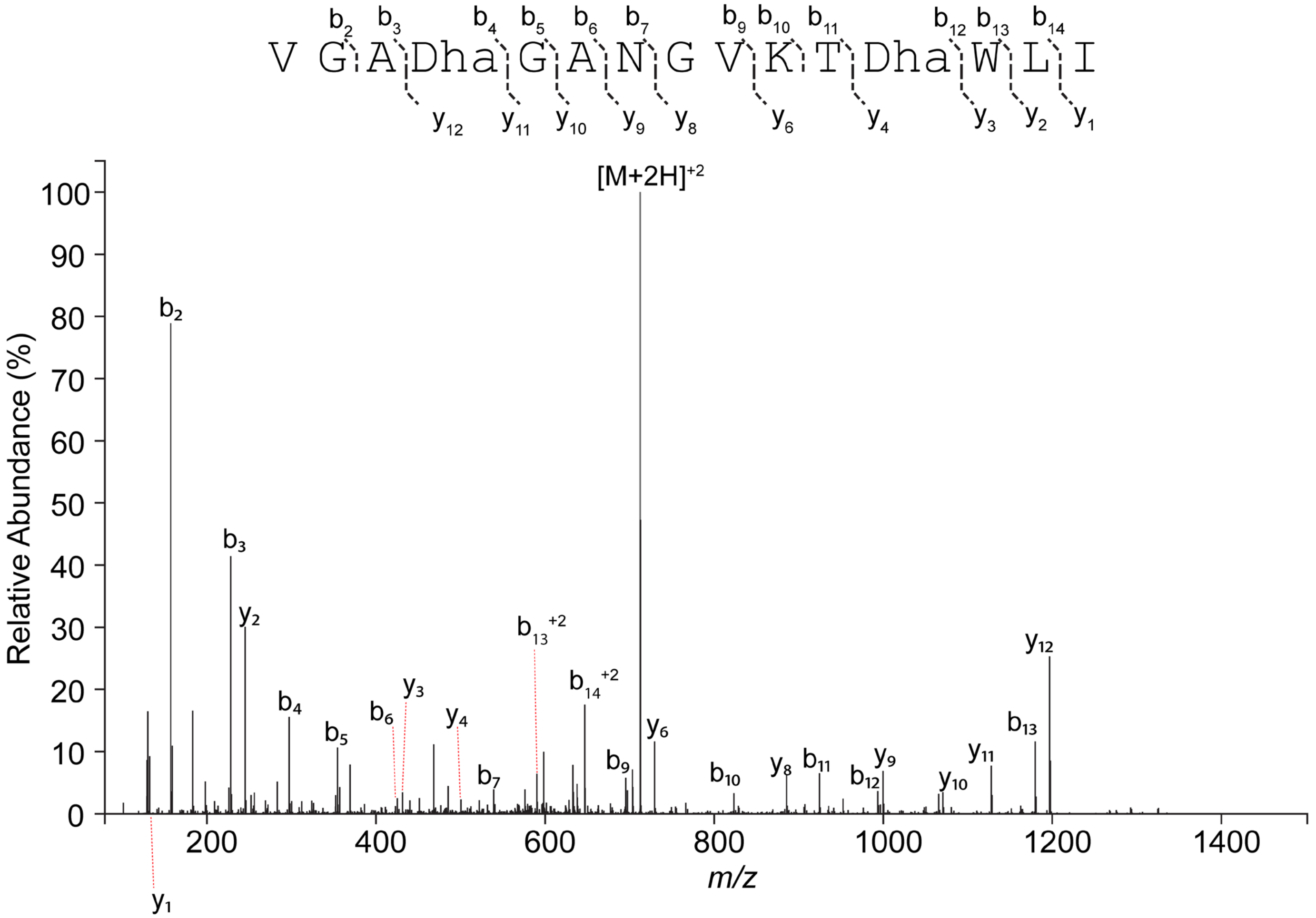

V G A Dha G A N G V K T Dha W L I
b2 b3 b4 b5 b6 b7 b9 b10 b11 b12 b13 b14
y12 y11 y10 y9 y8 y6 y4 y3 y2 y1
[M+2H]+2
Relative Abundance (%)
m/z
b13+2
b14+2

Figure S42: LC-HR-ESI-MS/MS analysis of macrocyclized products of Thr-containing substrates in Figure S39. The structure of each macrocyclized peptide is shown in each panel. A table comparing observed and theoretical *m/z* values for fragments may be found in **Supplementary Dataset 2**.

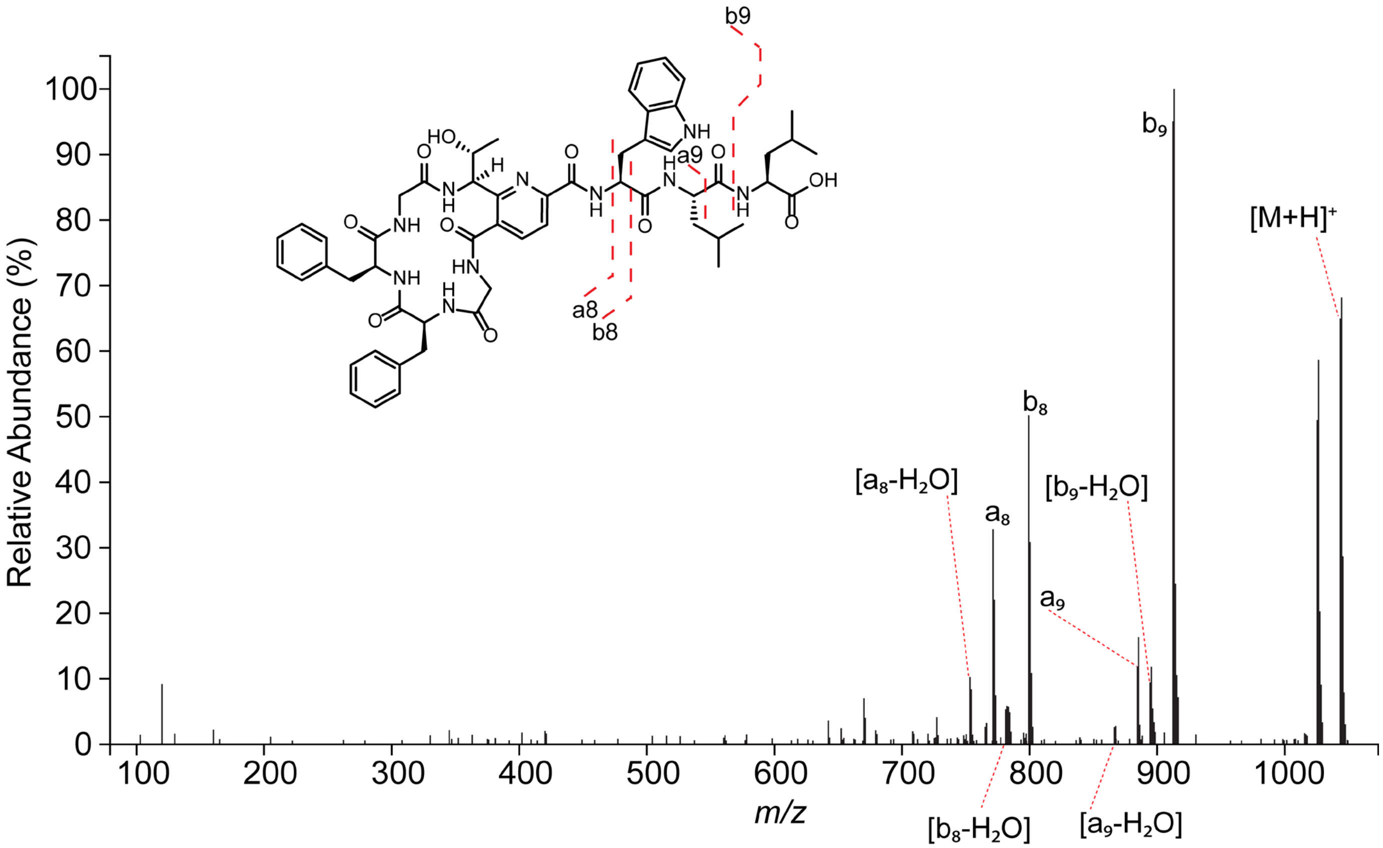

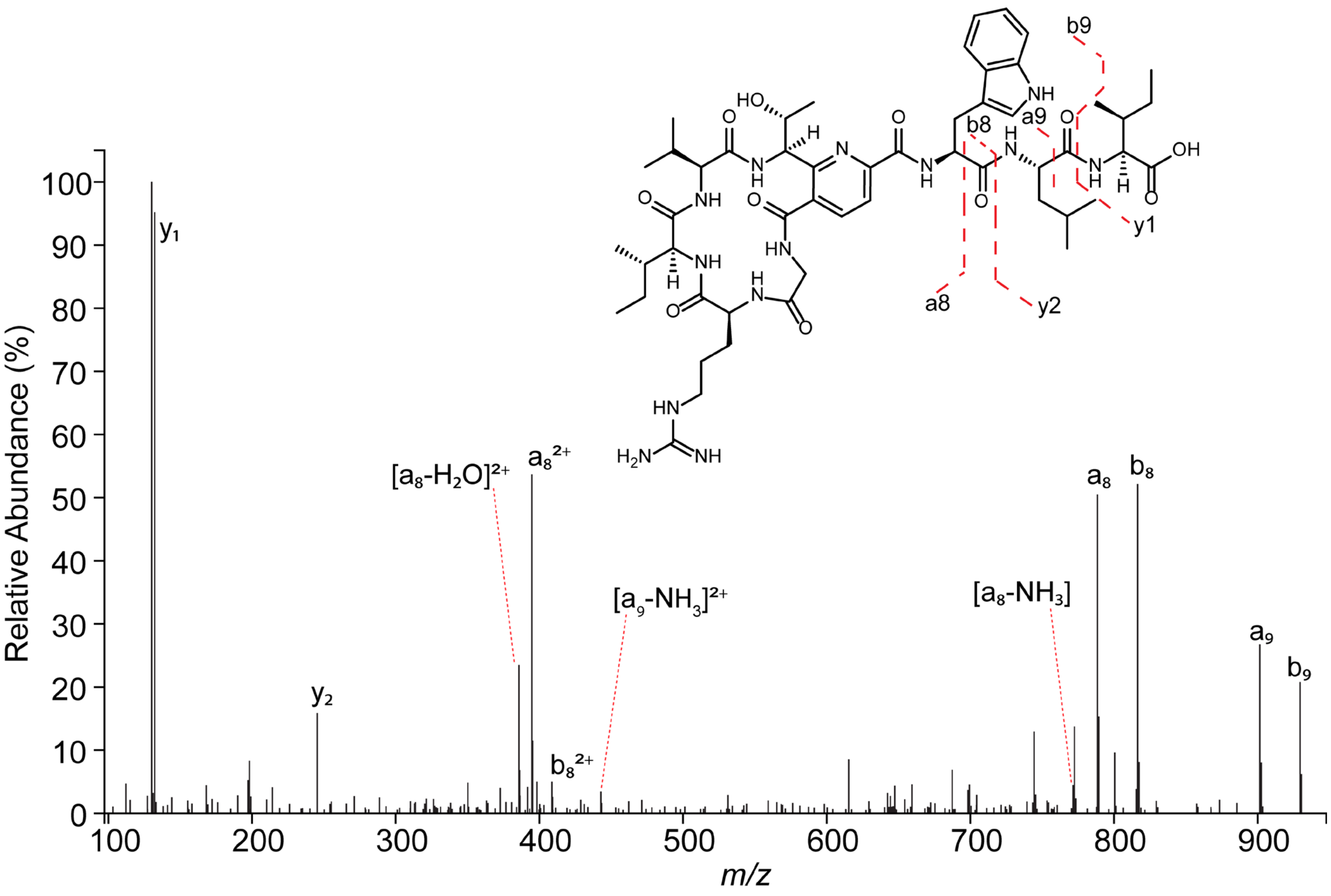

Relative Abundance (%)
m/z
y₁
y₂
[a₈-H₂O]²⁺
a₈²⁺
b₈²⁺
[a₉-NH₃]²⁺
[a₈-NH₃]
a₈
b₈
a₉
b₉

Figure S43: MroB/C/D do not produce 8- and 11-membered macrocycles. All results are acquired using reflector positive mode MALDI-TOF-MS. Unless otherwise stated, all peaks are $[M+H]^+$. The precursor peptides were generated through *in vitro* translation (see Experimental Methods). The f in the precursor peptide sequence represents a formyl group, which results from formyl-methionine utilized in *in vitro* translation. The remaining didehydrated intermediates are annotated accordingly. The resulting pyritides and ejected leader peptides were not observed. The asterisk (*) in the mass spectrum represents a +16 Da species caused by oxidation during sample preparation. [15] The # mark in the mass spectrum represents a -17 Da MALDI artifact, a result of deamination specific to reflector positive mode in MALDI-TOF-MS.[13, 14]

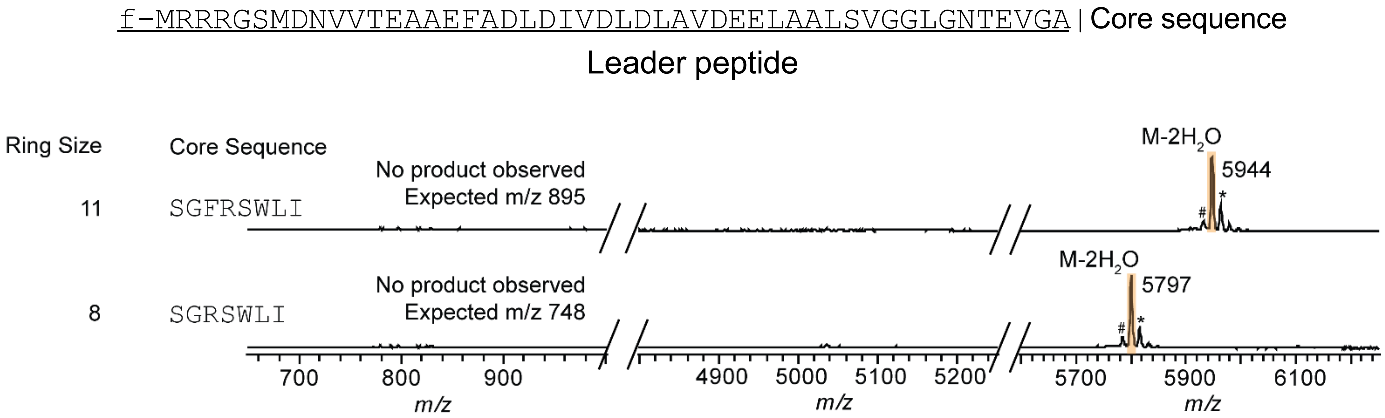

Figure S44: LC-HR-ESI-MS/MS analysis of a 32-membered macrocycle produced by MroB/C/D. The product was generated through MroB/C/D reaction with substrate synthesized in a 15 µL scale *in vitro* translation. A table comparing observed and theoretical m/z values for fragments may be found in **Supplementary Dataset 2**.

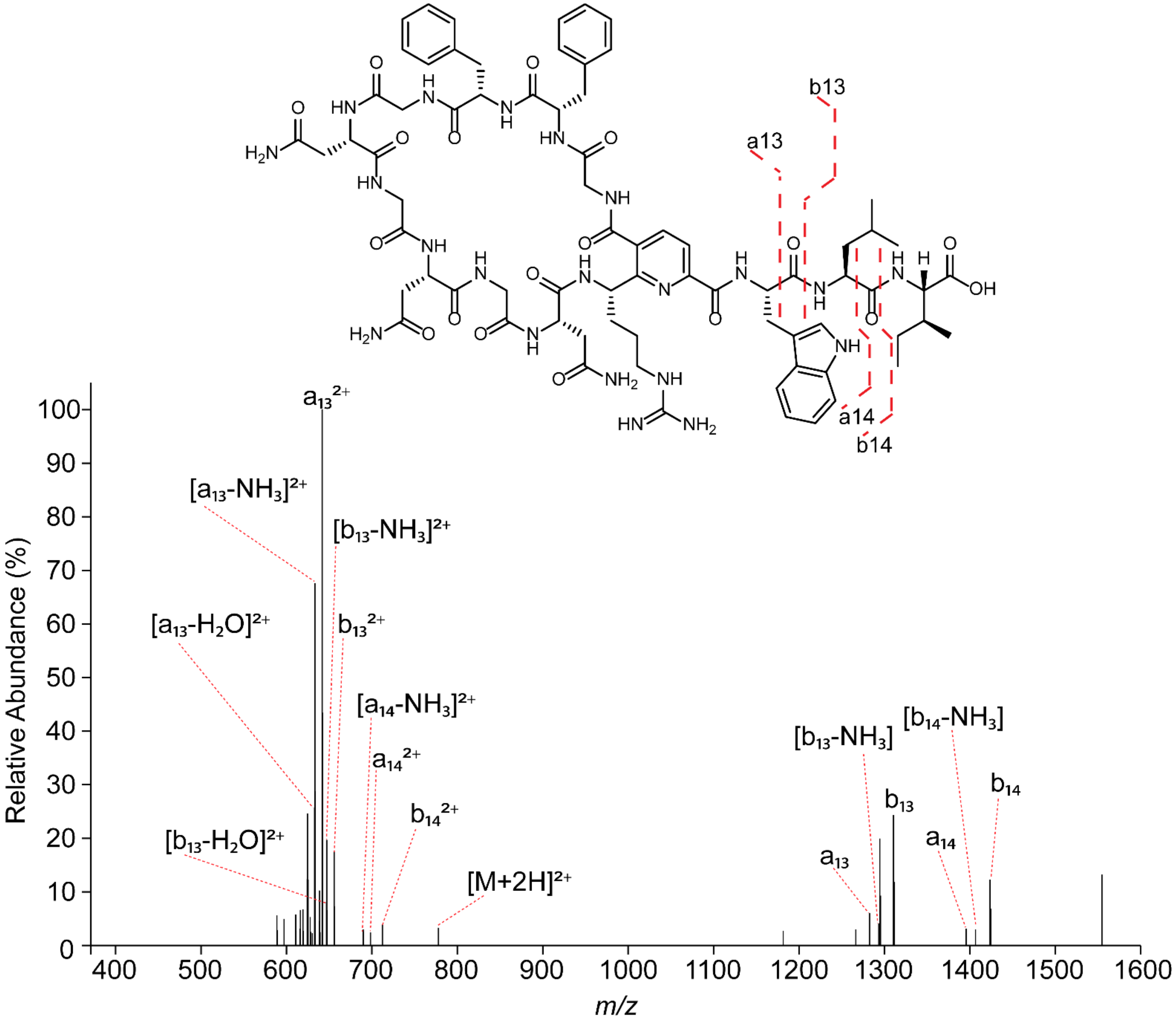

Figure S45: LC-HR-ESI-MS/MS analysis of a 38-membered macrocycle produced by MroB/C/D. The product was generated through MroB/C/D assays with substrate synthesized in a 15 µL scale *in vitro* translation. A table comparing observed and theoretical m/z values for fragments may be found in **Supplementary Dataset 2**.

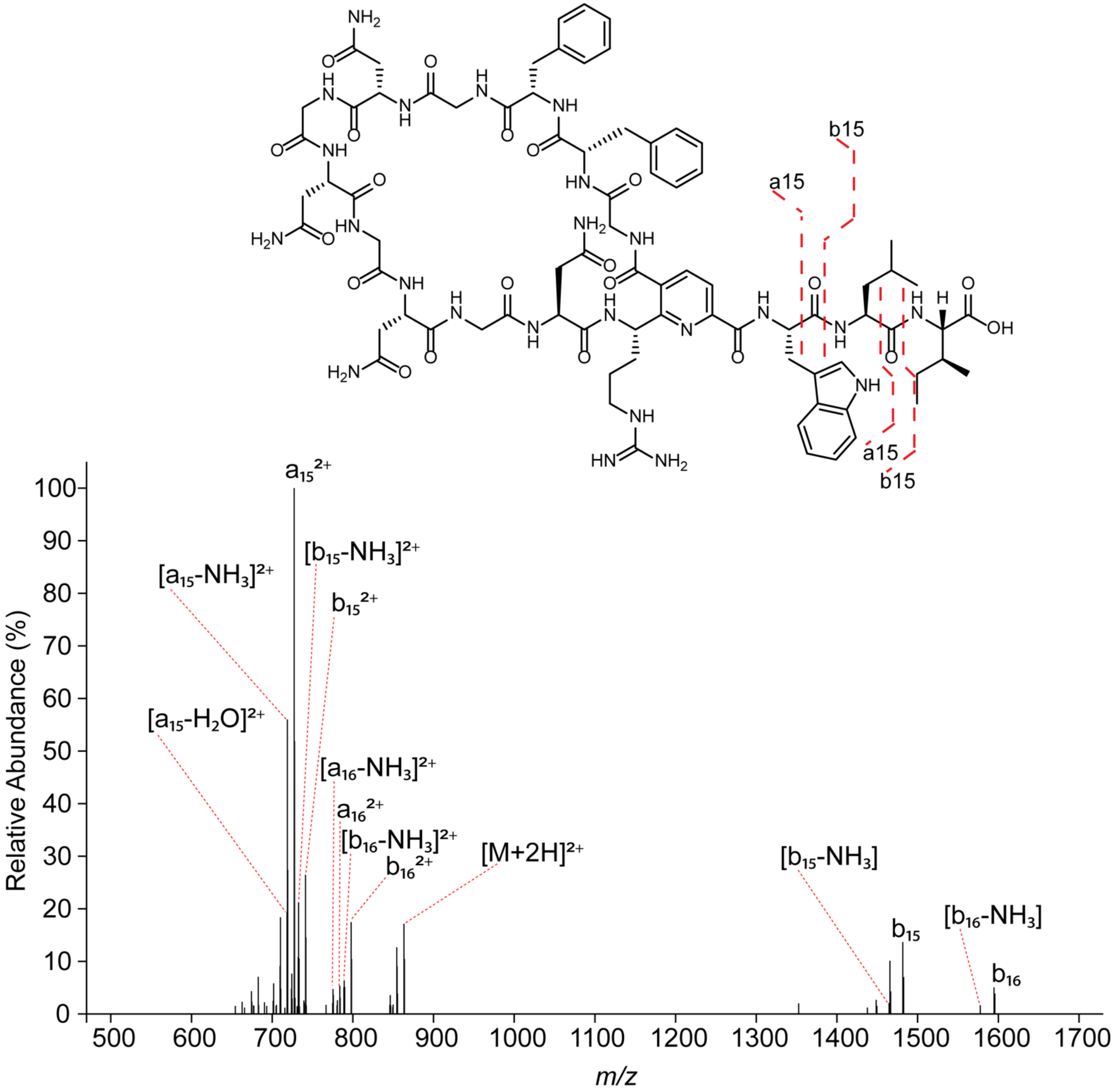

Figure S46: LC-HR-ESI-MS/MS analysis of a 62-membered macrocycle produced by MroB/C/D. The product was generated through MroB/C/D assays with substrate synthesized in a 15 µL scale *in vitro* translation. A table comparing observed and theoretical m/z values for fragments may be found in **Supplementary Dataset 2**.

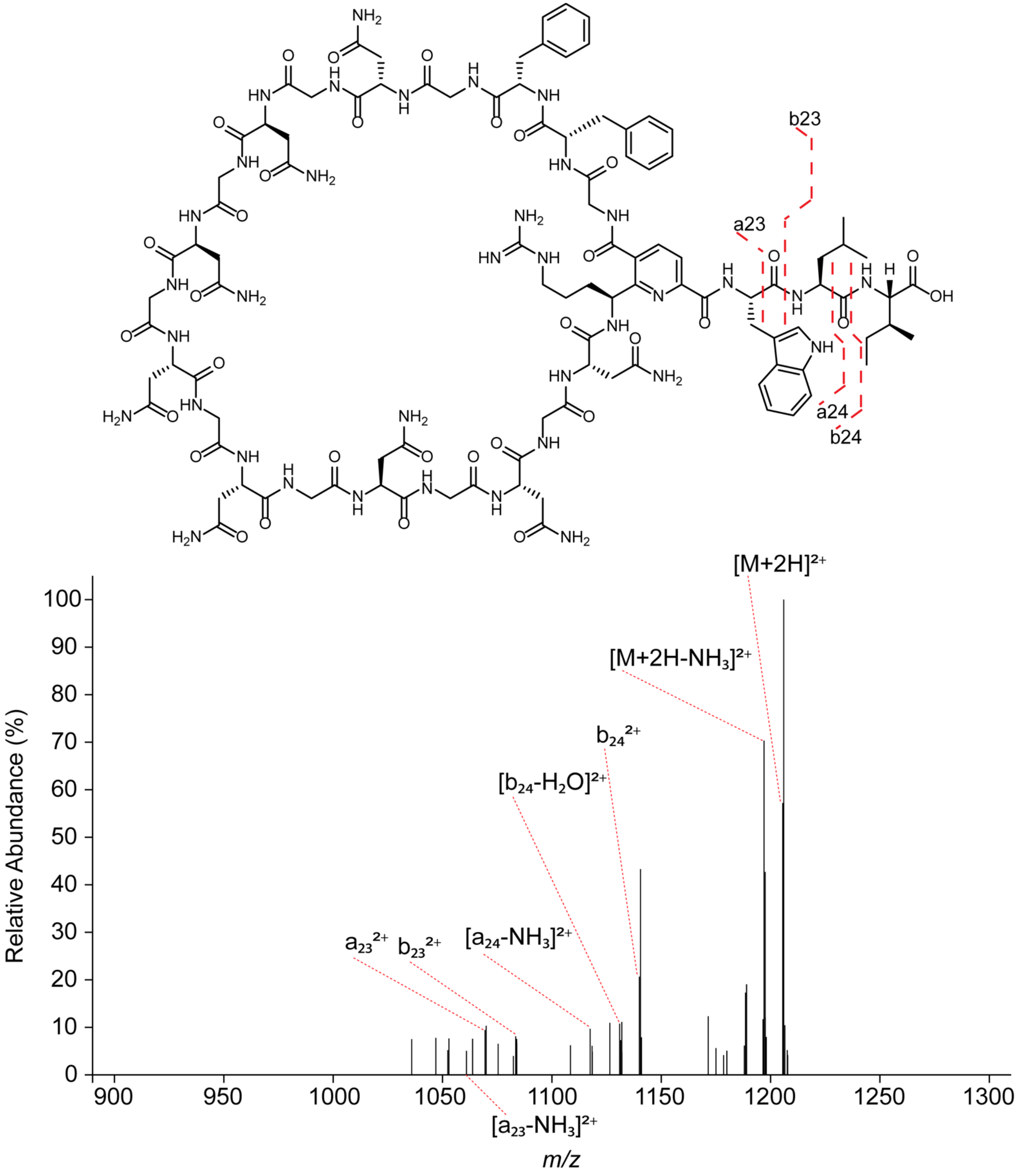

Figure S47: LC-HR-ESI-MS/MS analysis of a 68-membered macrocycle produced by MroB/C/D. The product was generated through MroB/C/D assays with substrate synthesized in a 15 µL scale *in vitro* translation. A table comparing observed and theoretical m/z values for fragments may be found in **Supplementary Dataset 2**.

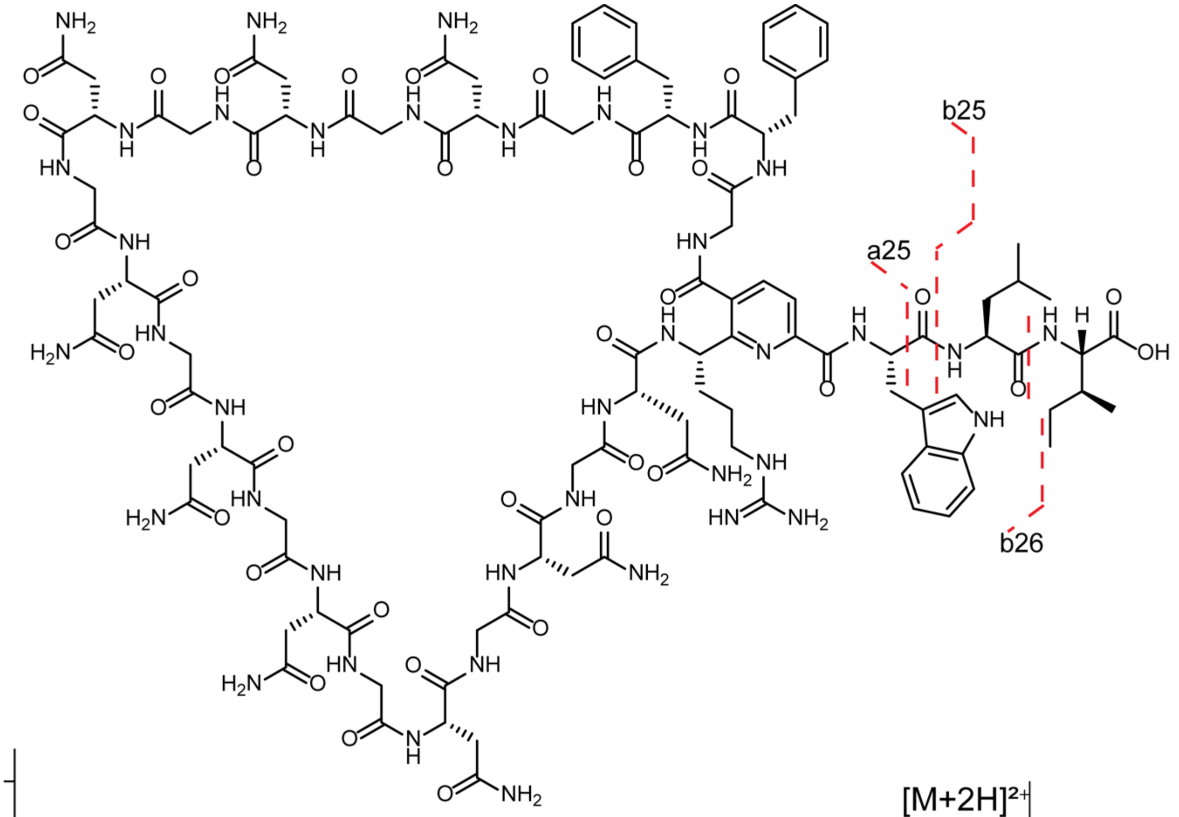

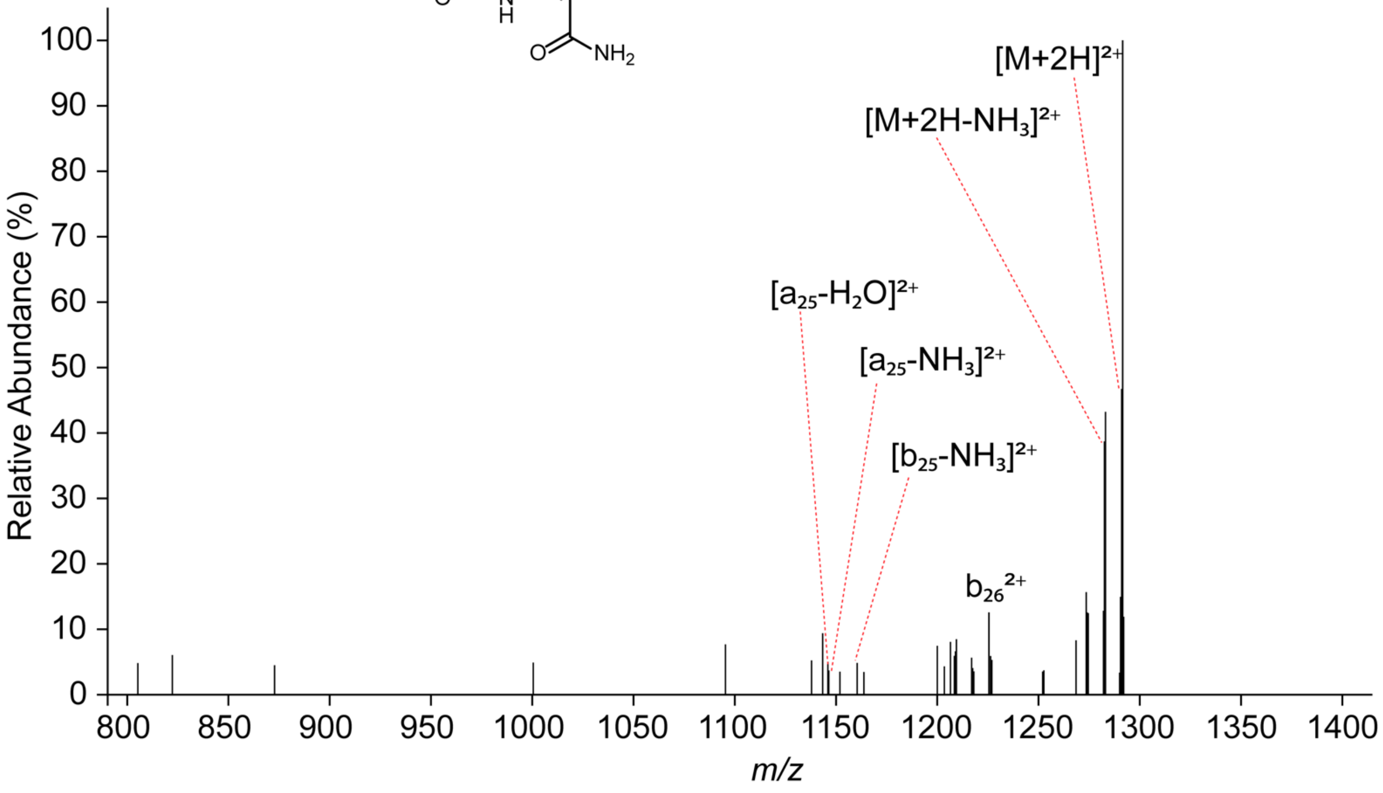

Figure S48: Large macrocycle sizes produced by MroB/C/D. All results were acquired using reflector positive mode MALDI-TOF-MS. The crystallization matrix utilized in this experiment was Super DHB. Unless otherwise stated, all peaks are $[M+H]^+$. The precursor peptides were generated through *in vitro* translation (see Experimental Methods). The f in the precursor peptide sequence represents a formyl group, which results from formyl-methionine utilized in *in vitro* translation. The pyritide macrocycles and the ejected leader peptides are annotated accordingly.

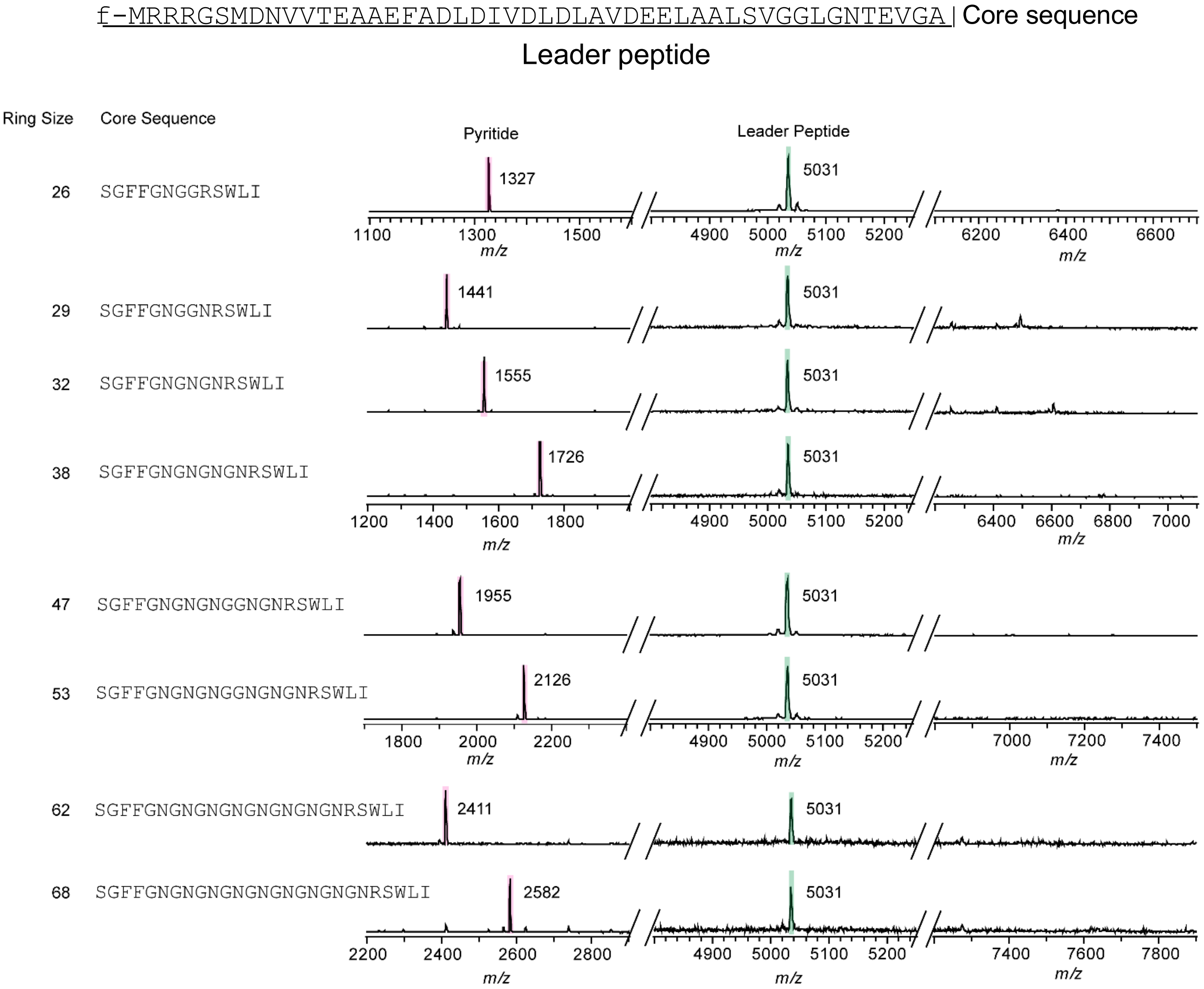

Figure S49: MroB/C/D produces 62-membered macrocycles with different sequences. The core sequences are indicated next to each mass spectrum. The different region between each sequence is highlighted in blue in the full-length precursor peptide (X = random amino acids except Cys, Ser, Thr). All results were acquired using reflector positive mode MALDI-TOF-MS. Unless otherwise stated, all peaks are $[M+H]^{+}$. The precursor peptides were generated through in vitro translation (see Experimental Methods). The f in the precursor peptide sequence represents a formyl group, which results from formyl-methionine utilized in in vitro translation. The pyritide macrocycles, the ejected leader peptides, the didehydrated intermediates, and the monodehydrated intermediates are annotated accordingly.

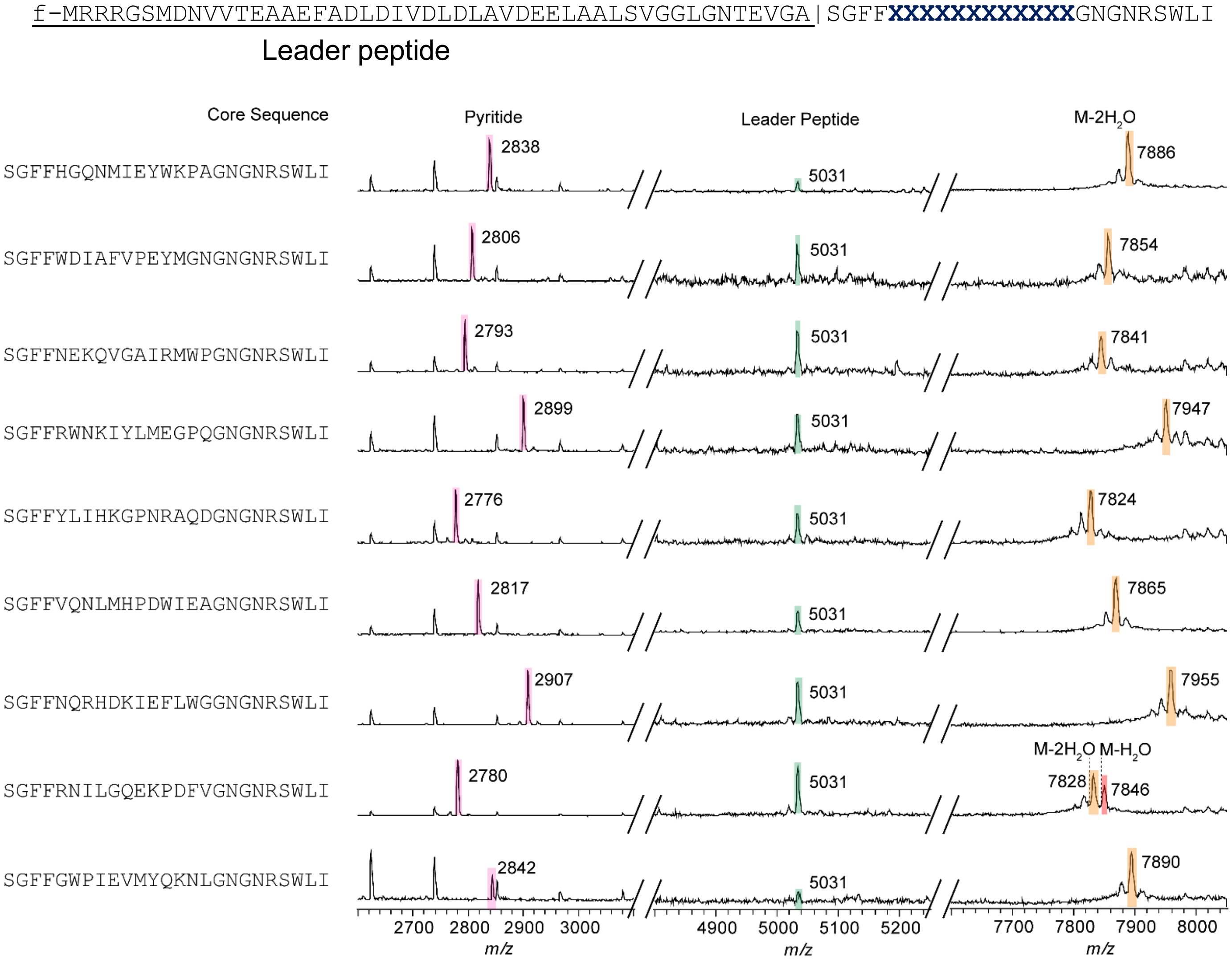

Figure S50: MroBCD produce a pyritide containing thiazol(in)es. (A) Incorporation of critical residues for thiazole-forming enzymes TbtE/F/G to MroA1 leader peptide and the design of the substrate processed by TbtE/F/G and MroB/C/D. Residues critical to TbtE/F/G activity are bolded in red.[16] Cys residues undergoing cyclodehydration by TbtEFG are bolded in blue, while Ser residues undergoing dehydration by MroBC are bolded in purple. (B) Mass spectral analysis of thiazol(in)e formation, dehydration, and cyclization. The sequence of the utilized precursor peptide is shown **(1)**. The f in the precursor peptide sequence represents a formyl group, which results from formyl-methionine utilized in in vitro translation. All spectra were acquired by reflector positive mode MALDI-TOF-MS. Unless otherwise stated, all peaks are $[M+H]^+$. The top MALDI-TOF mass spectrum shows the unmodified precursor peptide, which underwent five carbamidomethylations after treating with iodoacetamide (IAA). The third spectrum shows that five Cys residues were converted to four thiazoles and one thiazoline after treating with Tbt/E/F/G. This intermediate did not undergo carbamidomethylation after adding IAA (2). The fifth and sixth spectrum demonstrate that the precursor containing thiazol(in)e residues underwent two dehydrations by MroB/C followed by [4+2] cyclization by MroD. The last four spectra are different mass regions of 2 and 2 + MroD. CAM = carbamidomethyl.

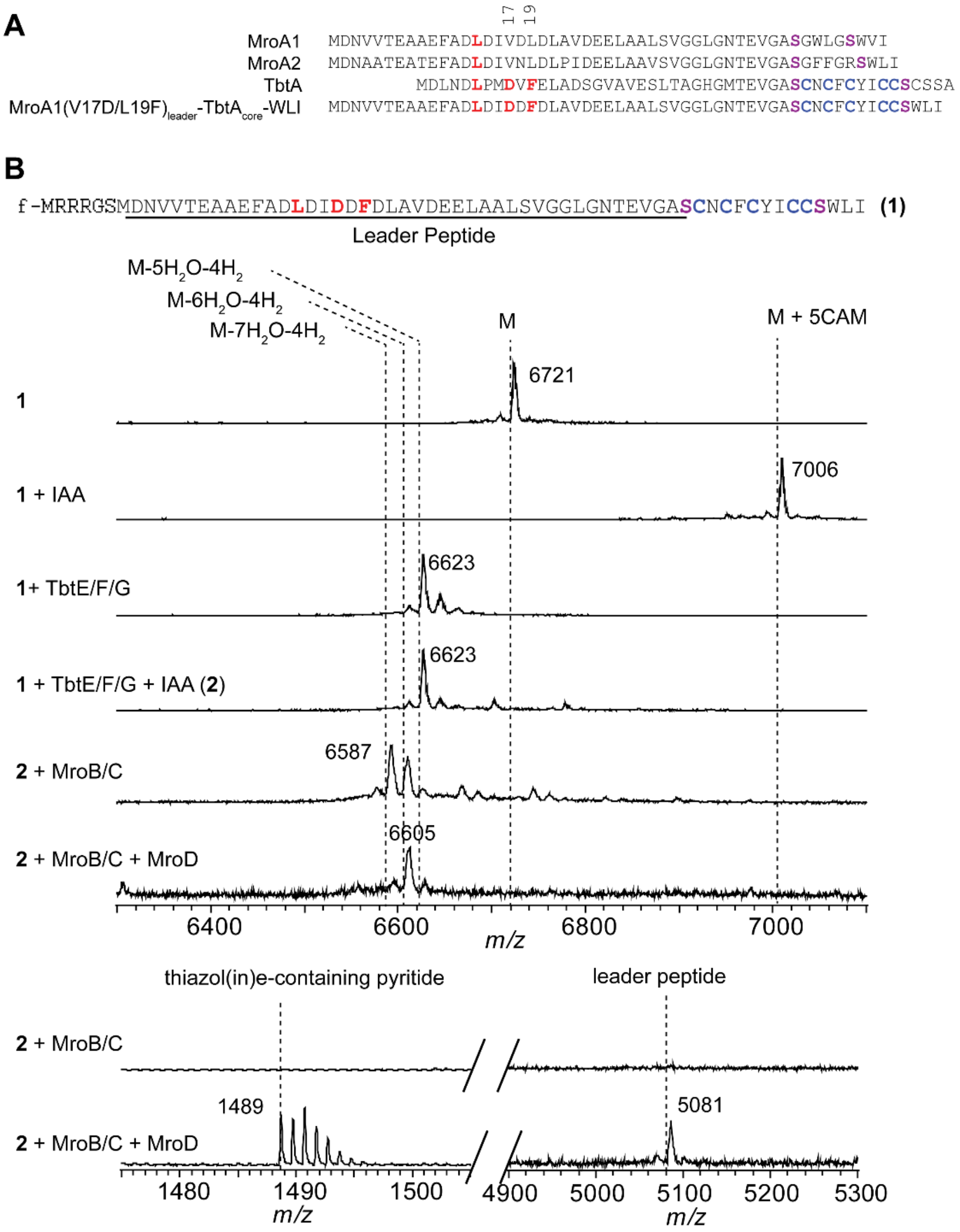

Figure S51: LC-HR-ESI-MS analysis of the thiazol(in)es-containing pyritide and ejected leader peptide (carboxamide). (A) The high-resolution mass spectrum of the pyritide containing four thiazoles and one thiazoline. Thz represents a thiazole residue. The asterisk demonstrates that any of these residues could be thiazoline, as the position of the one thiazoline residue has not yet been identified. The cyclization to form pyritide is colored orange. The thiazol(in)e residues are bolded in blue. (B) The high-resolution mass spectrum of the ejected leader peptide (carboxamide).

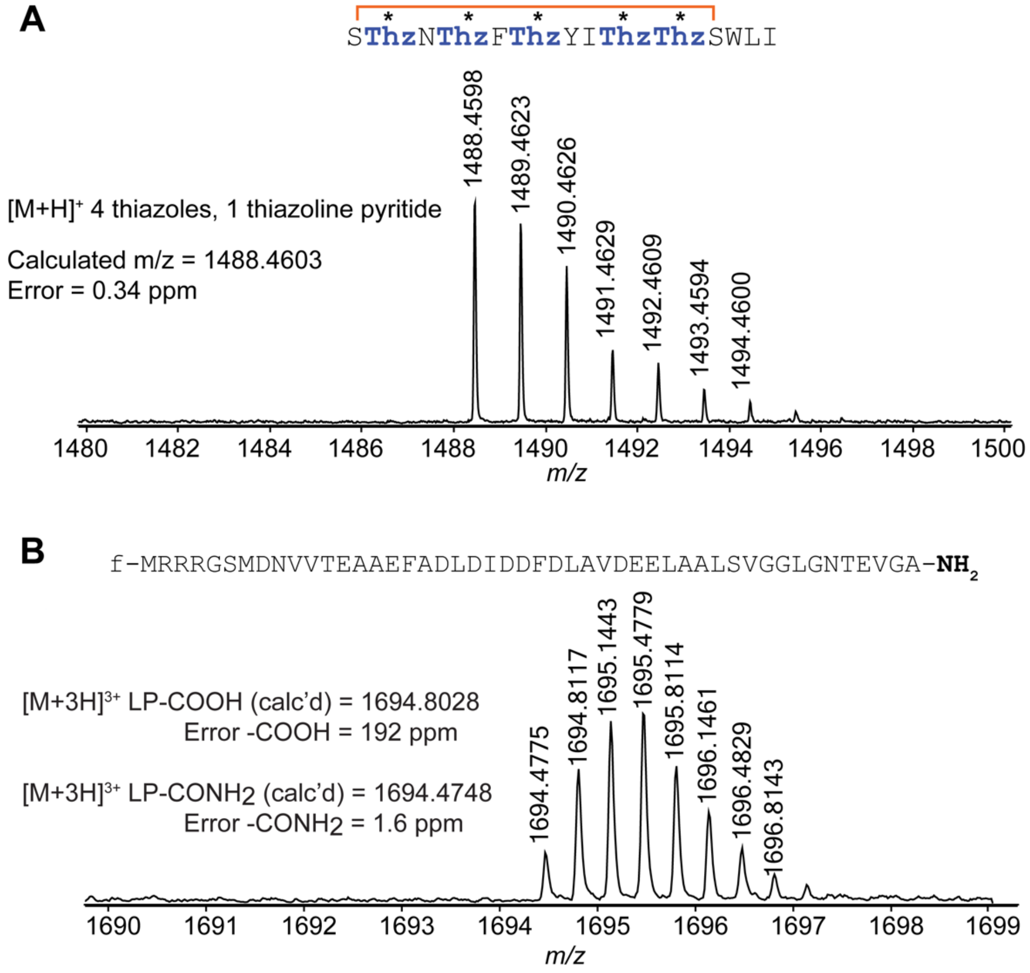

Figure S52: Δ12MroA1 is dehydrated by MroB/C and cyclized by MroD. The sequence of this precursor peptide is shown below. All spectra were acquired using reflector positive mode MALDI-TOF-MS. Unless otherwise stated, all peaks are $[M+H]^+$. The pyritide macrocycle, the ejected leader peptide, the didehydrated intermediate, and the unmodified precursor peptide are annotated accordingly. The x mark in the mass spectrum denotes $[M+Na]^+$ ions and the xx mark denotes $[M+K]^+$ ions.

SDLDIVDLDLAVDEELAALSVGGLGNTEVGA|SGWLGSWVI

Leader Peptide

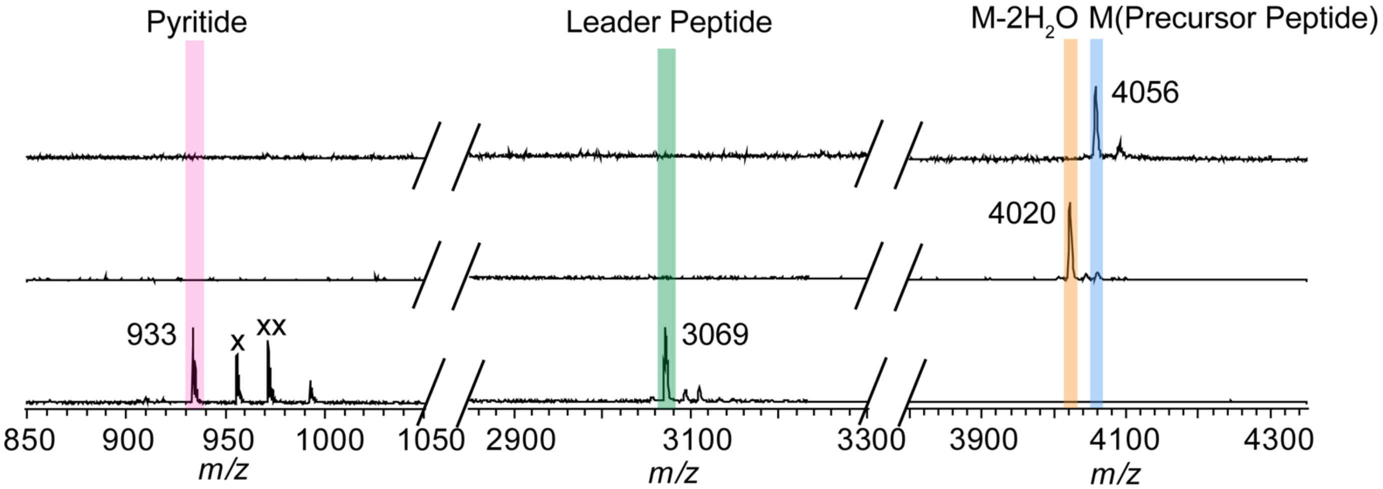

Figure S53: Fluorescence polarization of MBP-MroB and MBP-MroD binding to fluorescein-Δ12MroA1. Fluorescence polarization (FP) binding experiments were performed using N-terminal fluorescein-labeled Δ12MroA1. Errors on K_D values are the standard error of the mean generated by regression analysis; error bars represent standard deviation (n=3).

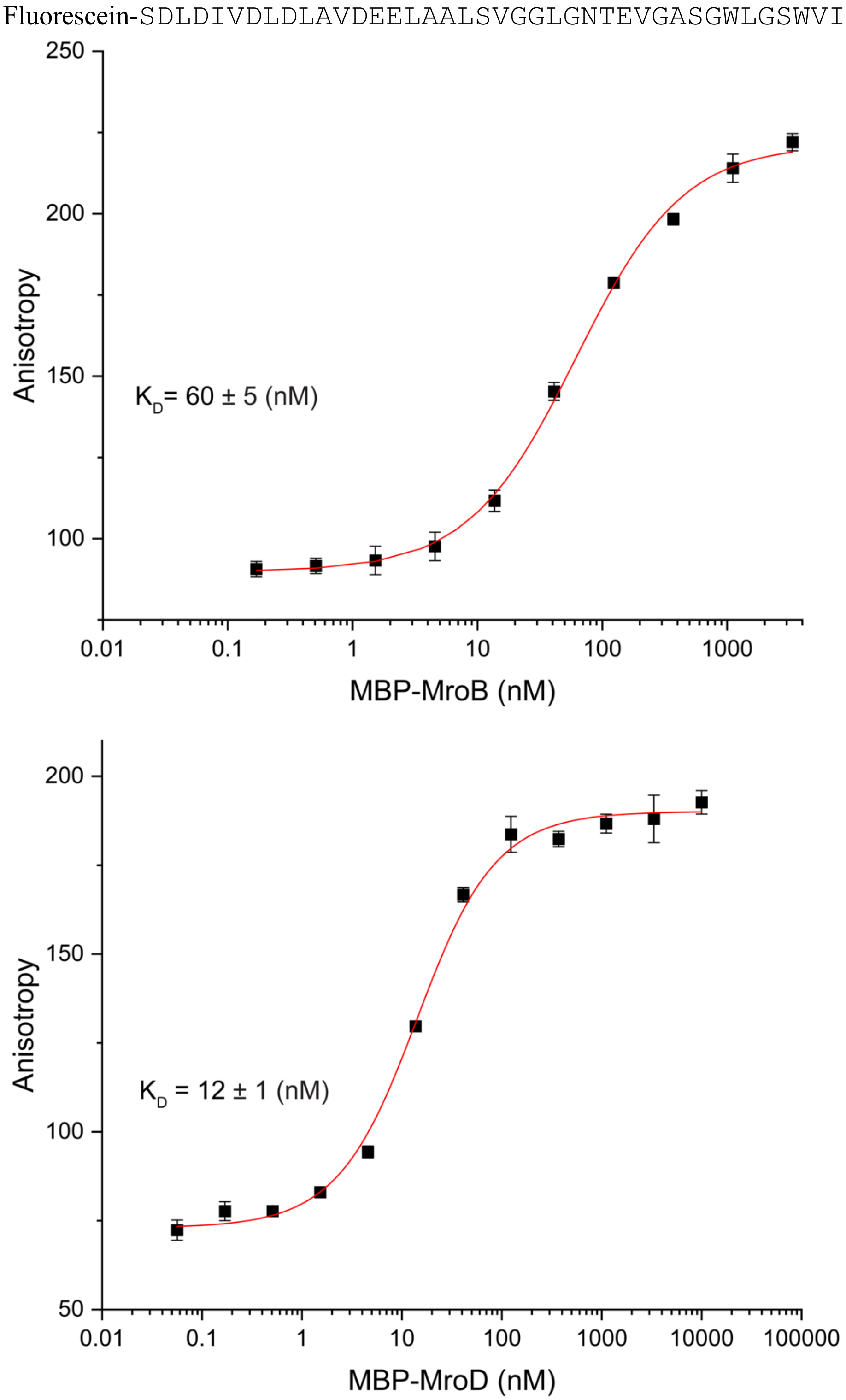

Figure S54: Both leader and core region are required for MroB binding. Competitive fluorescence polarization traces of MroA1 variants binding to MroB. IC_{50} values and sequences of each peptide competitor are summarized in Table 1. These peptides were titrated against MBP-MroB (80 nM) complexed with fluorescein-Δ12MroA1 (5 nM). MBP-MroB shows considerably greater affinity for Δ12MroA1 containing both leader and core than Δ12MroA1 leader peptide (~28-fold reduction in affinity) or GlyAla-MroA1core (~40-fold reduction affinity). Error bars represent standard deviation (n=3). Errors on K_i and IC_{50} values are the standard error of the mean generated through regression analysis.

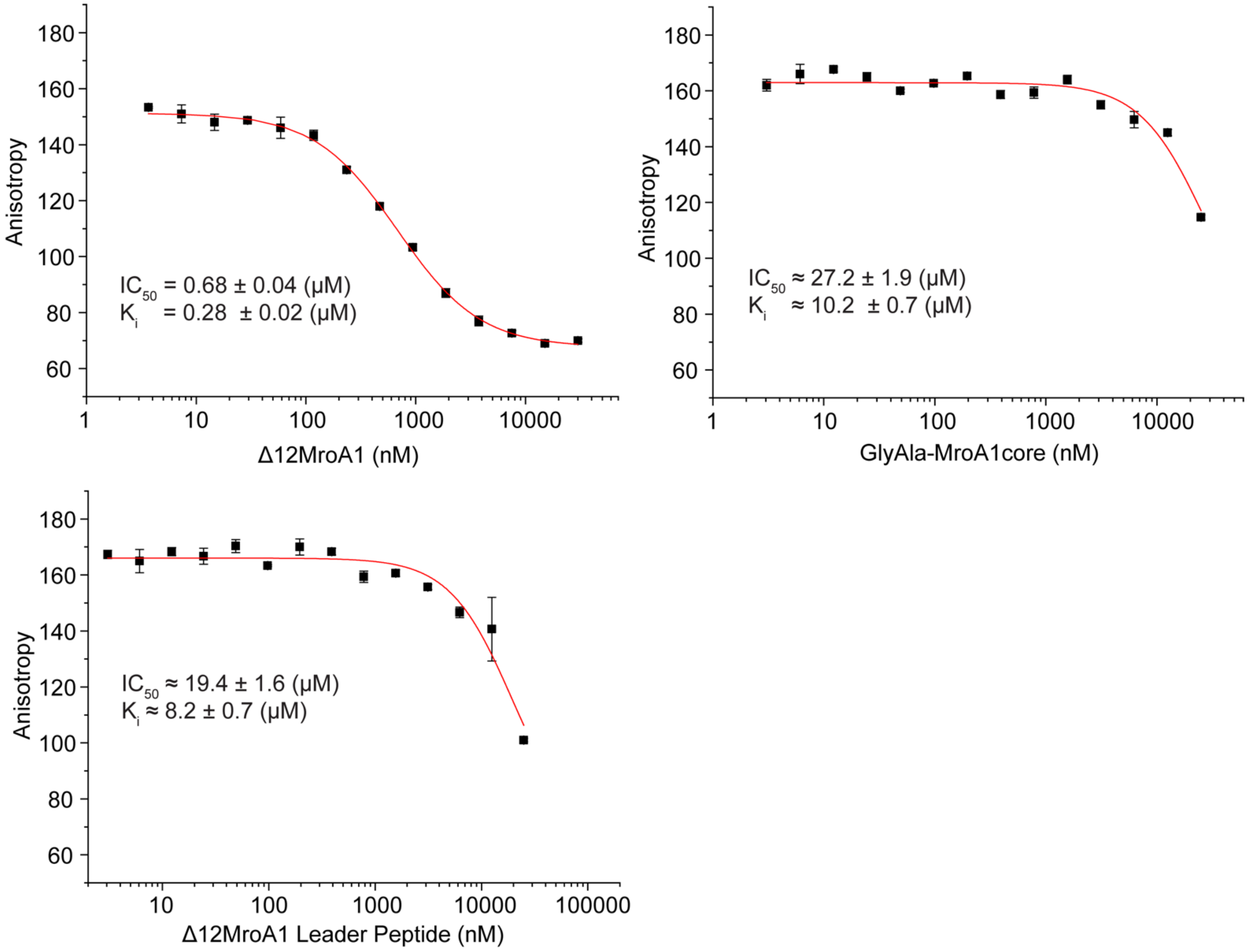

Figure S55: Both leader and core region are required for MroD binding. Competitive fluorescence polarization traces of MroA1 variants binding to MroB. IC_{50} values and sequences of each peptide competitor are summarized in Table 1. These peptides were titrated against MBP-MroD (80 nM) complexed with fluorescein-Δ12MroA1 (5 nM). MBP-MroD shows considerably greater affinity for Δ12MroA1 containing both leader and core than Δ12MroA1 leader peptide (~178-fold reduction in affinity) or GlyAla-MroA1core (~455-fold reduction affinity). Error bars represent standard deviation (n=3). Errors on K_i and IC_{50} values are the standard error of the mean generated through regression analysis.

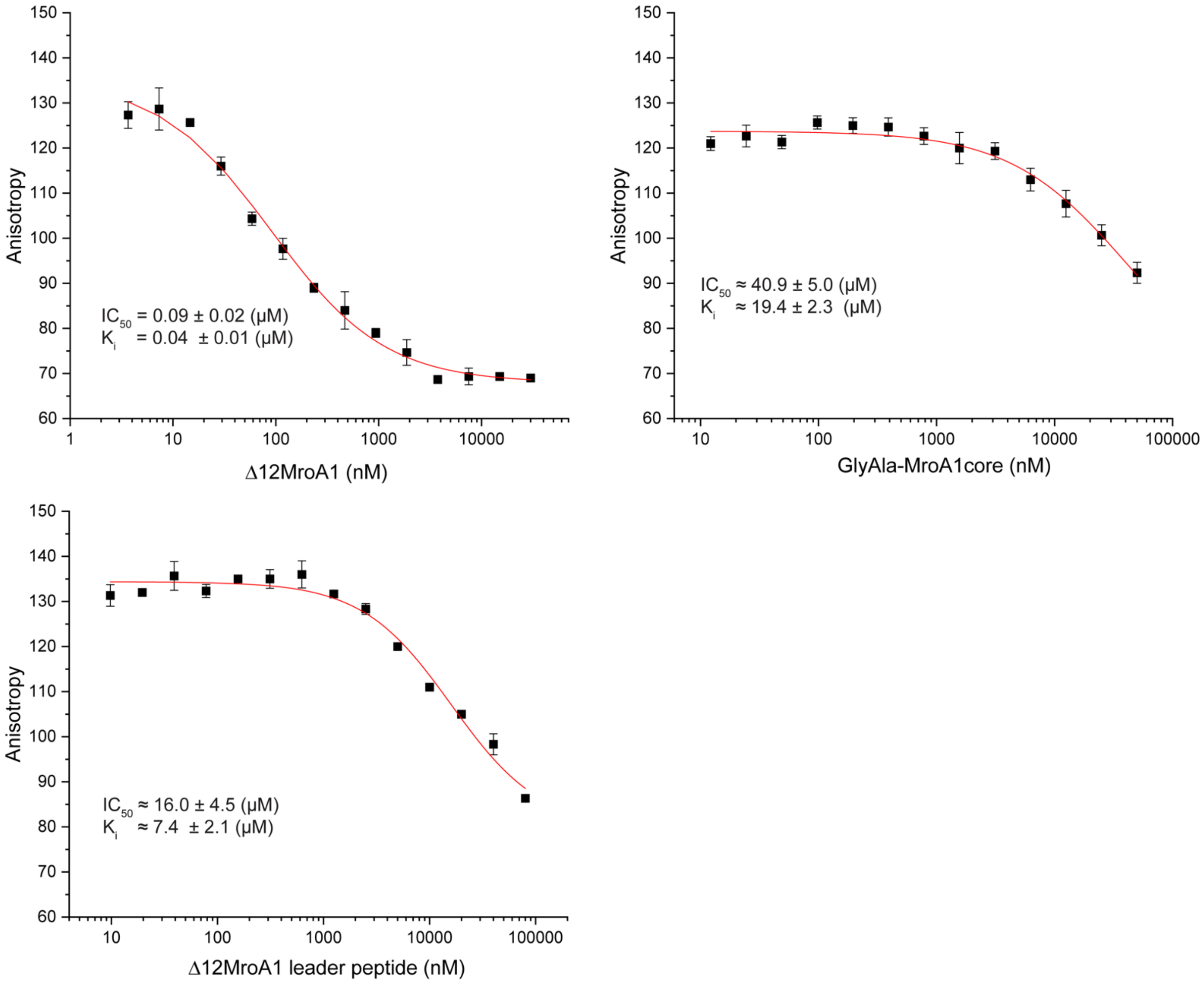

Figure S56: The C-terminal tripeptide motif is important for MroB binding. Competitive fluorescence polarization traces of MroA1 variants binding to MBP-MroB. IC_{50} values and sequences of each peptide competitor are summarized in Table 1. These peptides were titrated against MBP-MroB (80 nM) complexed with fluorescein-Δ12MroA1 (5 nM). Error bars represent standard deviation (n=3). Errors on K_i and IC_{50} values are the standard error of the mean generated through regression analysis.

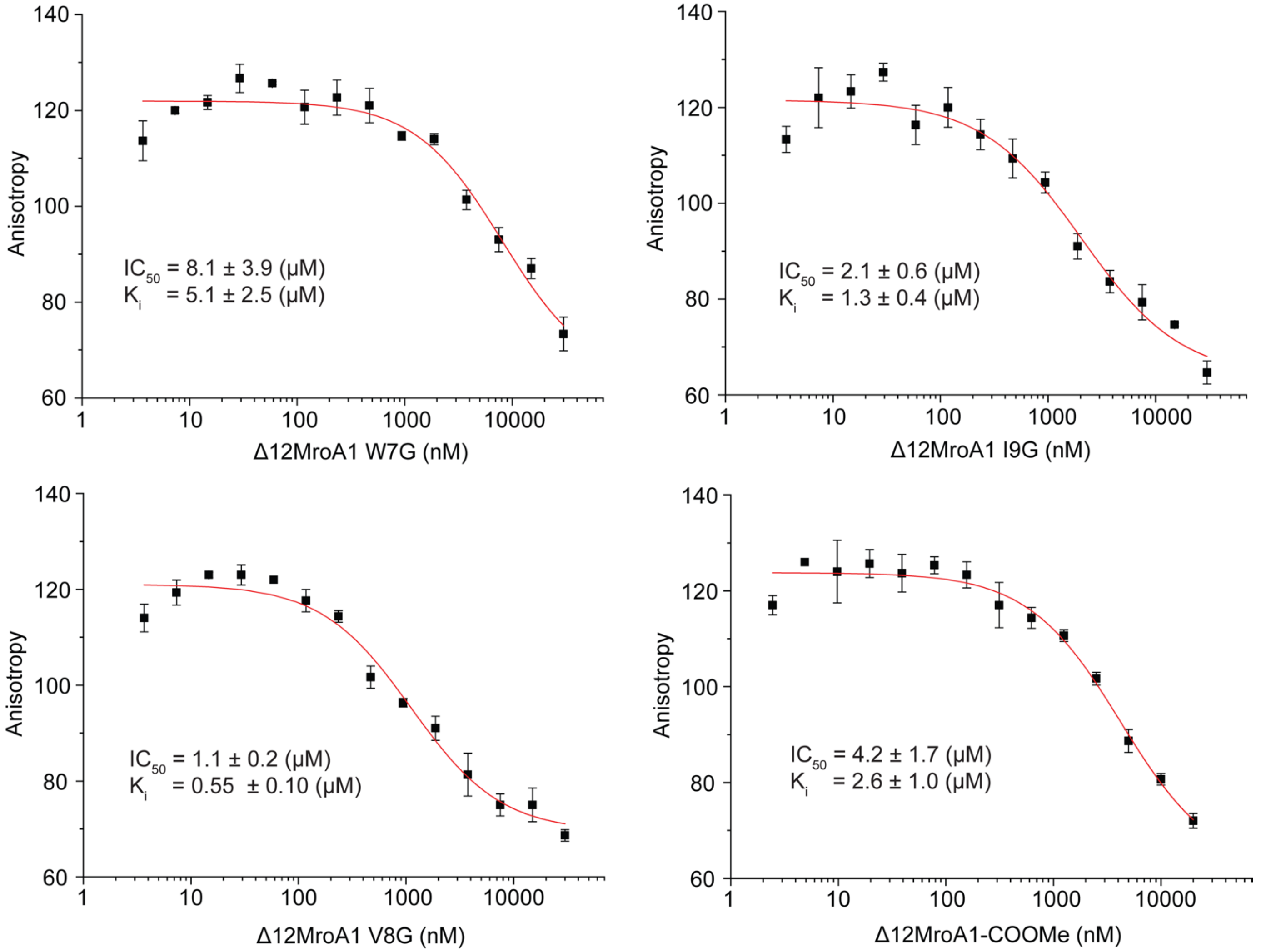

Figure S57: The C-terminal tripeptide motif is important for MroD binding. Competitive fluorescence polarization traces of MroA1 variants binding to MBP-MroD. IC_{50} values and sequences of each peptide competitor are summarized in Table 1. These peptides were titrated against MBP-MroD (80 nM) complexed with fluorescein-Δ12MroA1 (5 nM). Error bars represent standard deviation (n=3). Errors on K_i and IC_{50} values are the standard error of the mean generated through regression analysis.

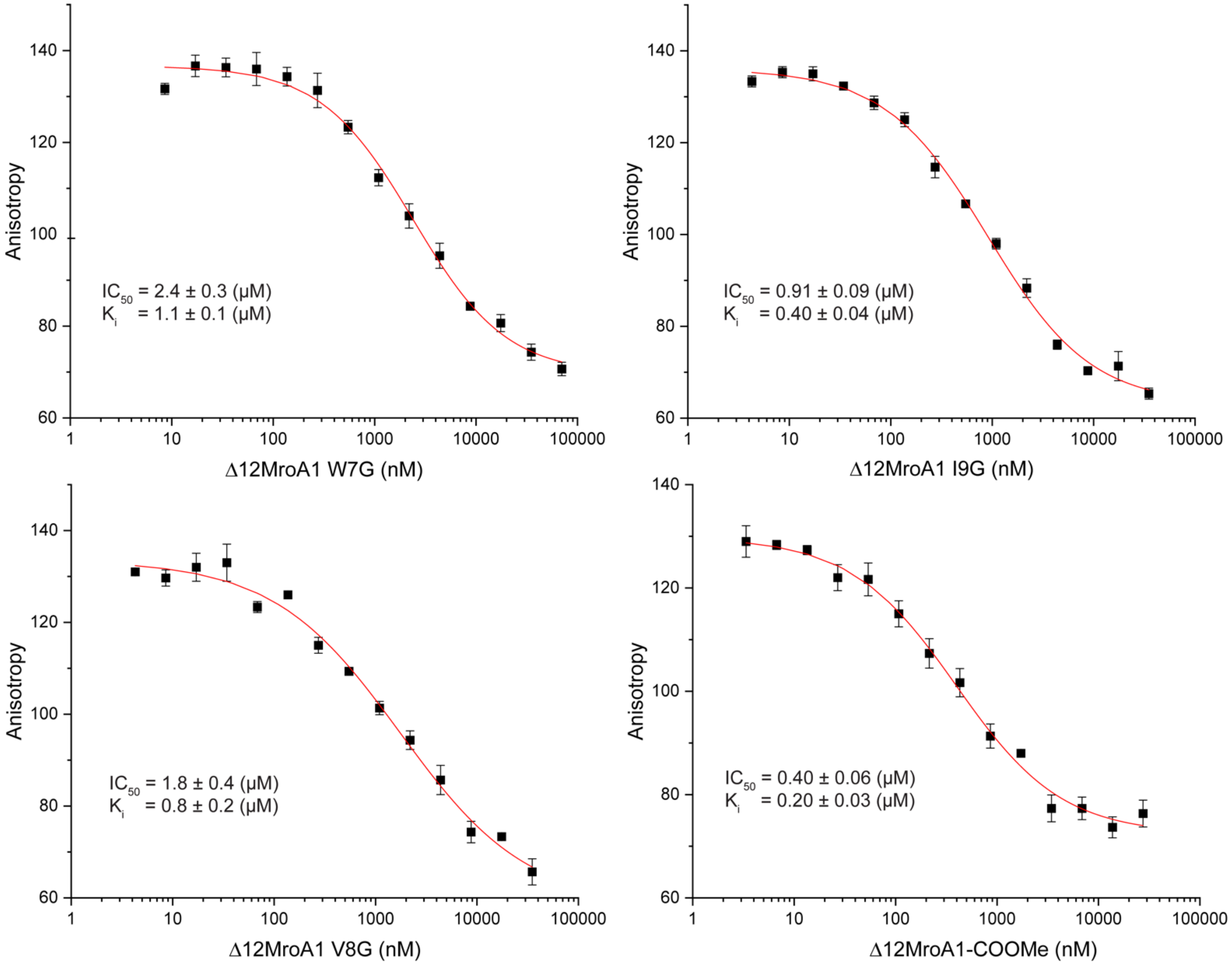

Figure S58: Extracted ion chromatogram (EIC) of the LC-MS trace of Δ12MroA1 W7G after MroB/C reaction. All detected ions have z = 3. (A) Overlaid EICs of nondehydrated, monodehydrated, and didehydrated Δ12MroA1 W7G. (B) The EIC of monodedehydrated Δ12MroA1 W7G. The tandem mass spectrum generated through CID analysis is shown in Figure 4A. (C) The EIC of nondehydrated Δ12MroA1 W7G. (D) The EIC of didehydrated Δ12MroA1 W7G.

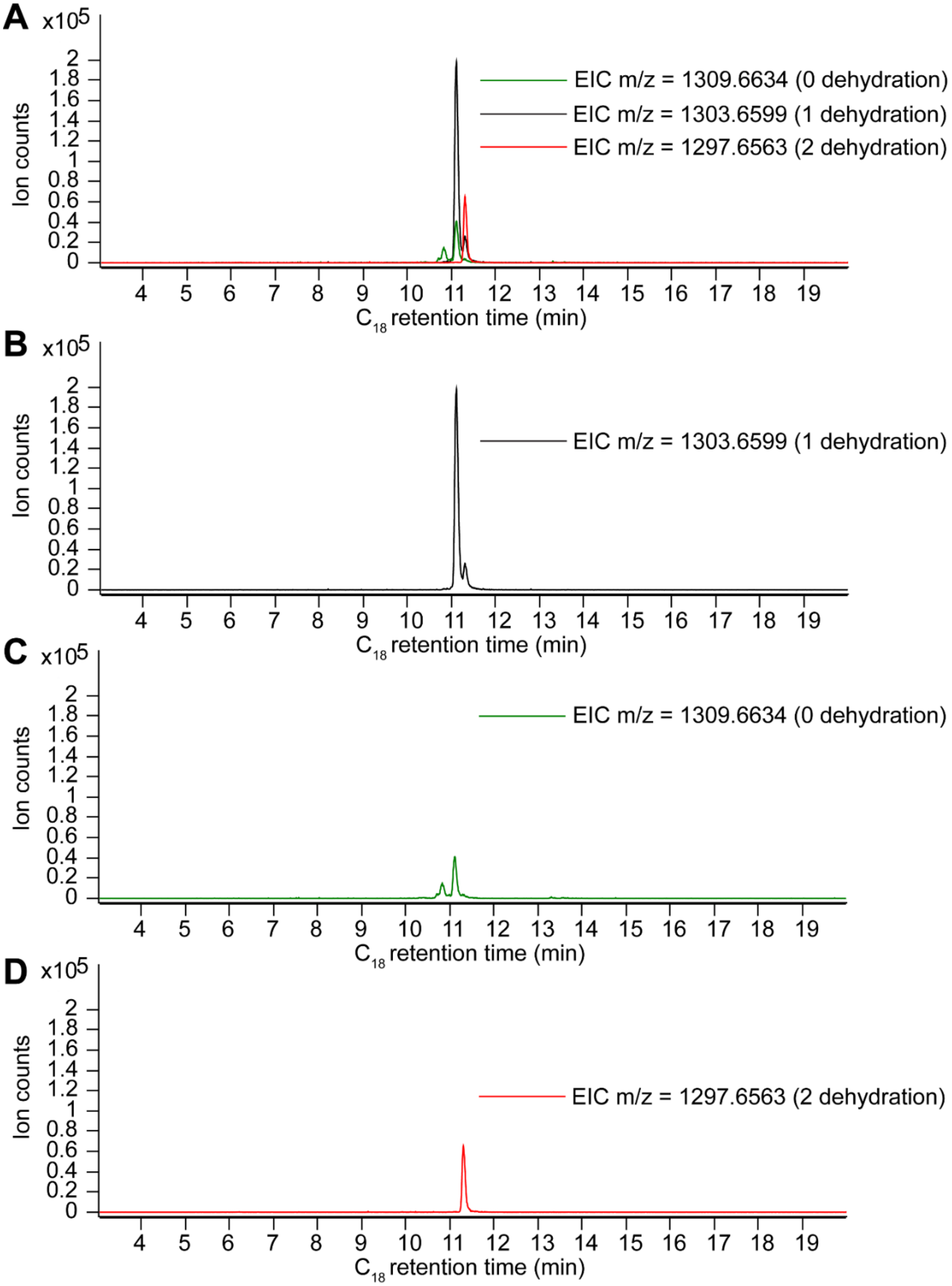

Figure S59: Extracted ion chromatogram (EIC) of the LC-MS trace of GlyAla-MroA1 core peptide after MroB/C reaction. All ions have z = 1. (A) Overlaid EICs of nondehydrated, monodehydrated, and didehydrated GlyAla-MroA1 core. (B) The EIC of monodedehydrated GlyAla-MroA1core. The tandem mass spectrum generated through CID analysis is depicted in Figure 4B. (C) The EIC of nondehydrated GlyAla-MroA1core. (D) The EIC of didehydrated GlyAla-MroA1 core.

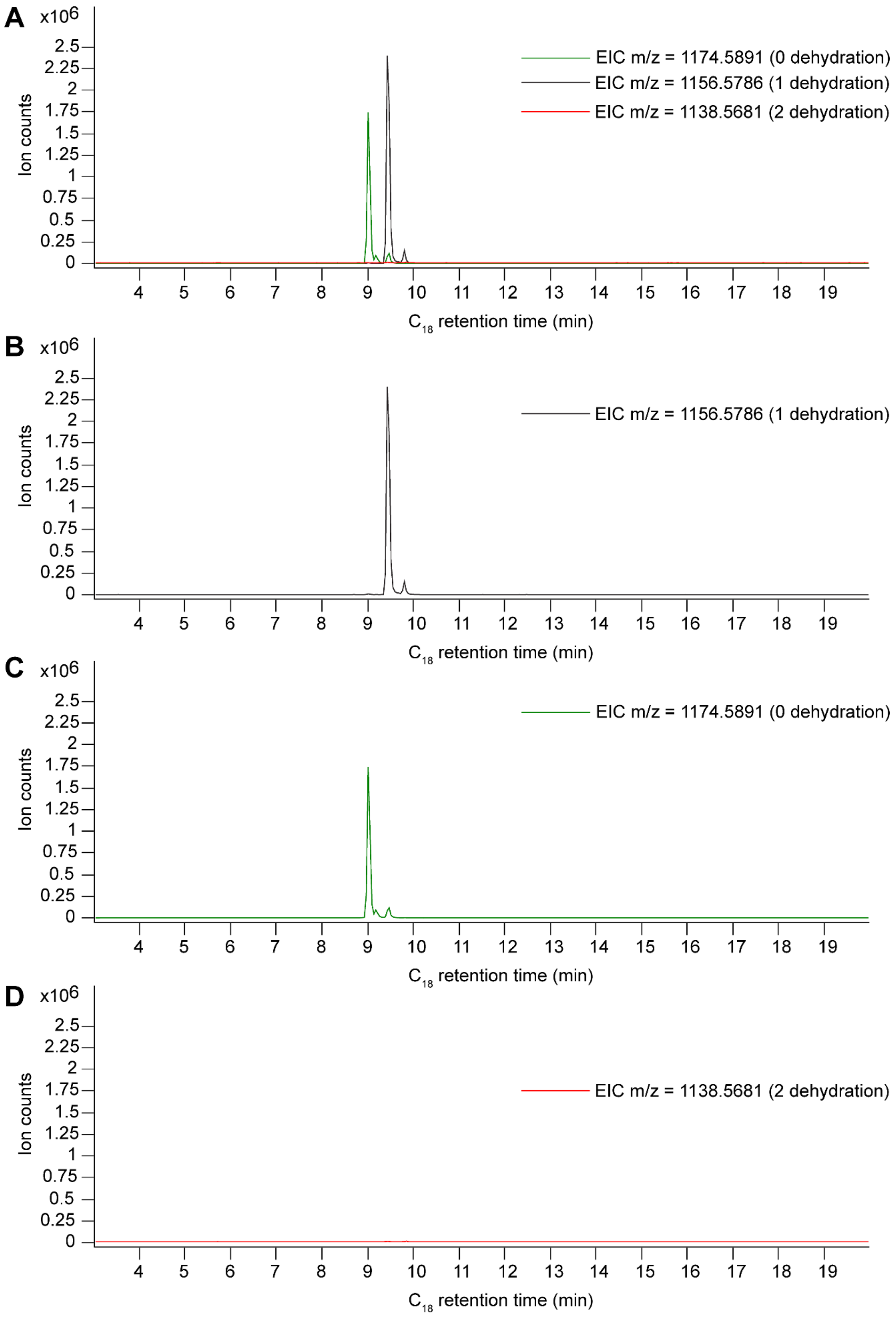

Figure S60: Trp8 and Ile10 are important for dehydration of Ser7 in MroA2. The unmodified precursor peptides and the dehydrated products are annotated accordingly. The # mark in the mass spectrum represents a minor -17 Da MALDI artifact, a result of deamination specific to reflector positive mode in MALDI-TOF-MS.[13, 14]

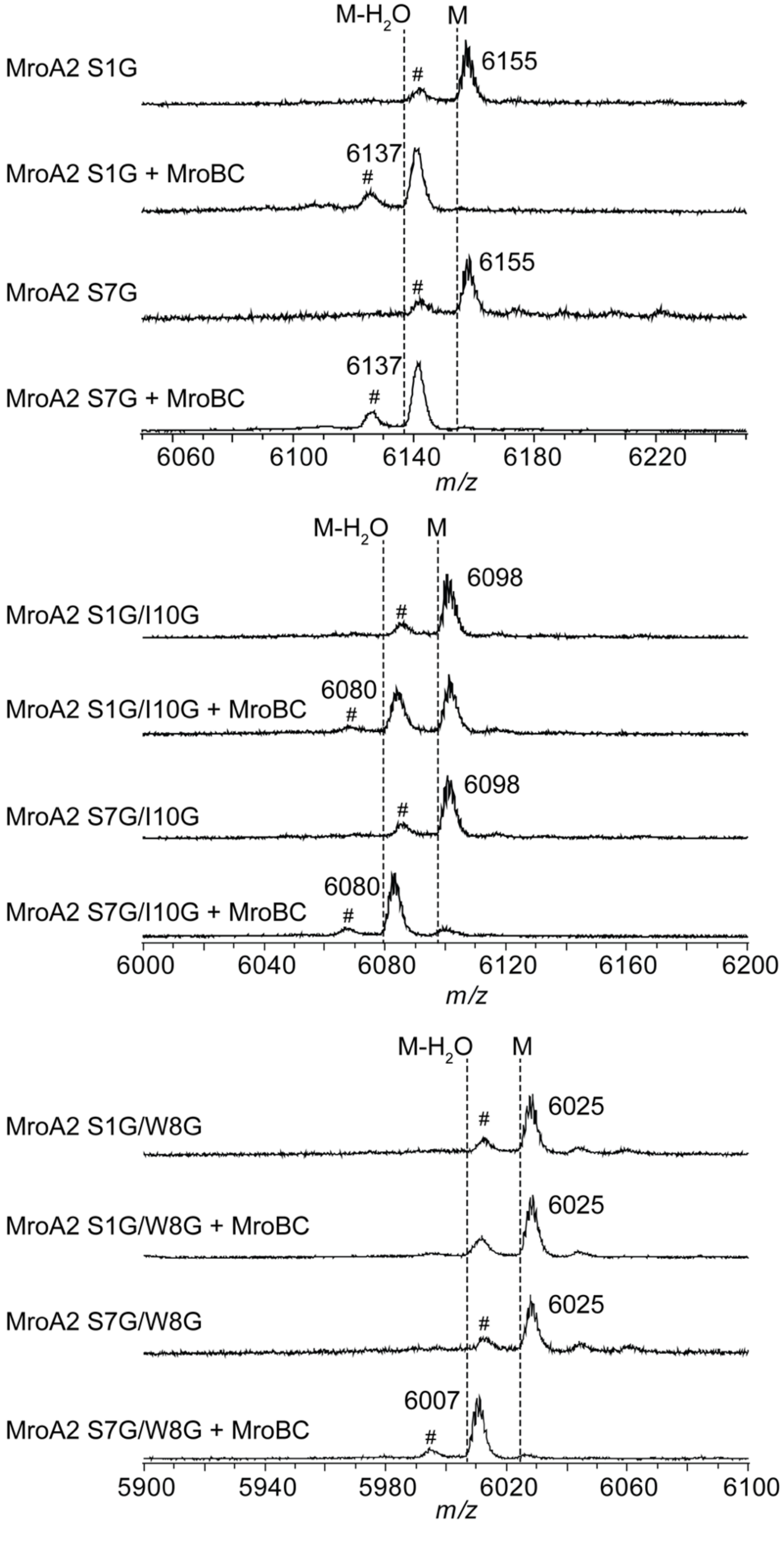

Supporting References

1. Gibson, D. G.; Young, L.; Chuang, R. Y.; Venter, J. C.; Hutchison, C. A., 3rd; Smith, H. O., Enzymatic assembly of DNA molecules up to several hundred kilobases. *Nat. Methods* **2009,** *6* (5), 343.

2. Hudson, G. A.; Hooper, A. R.; DiCaprio, A. J.; Sarlah, D.; Mitchell, D. A., Structure prediction and synthesis of pyridine-based macrocyclic peptide natural products. *Org. Lett.* **2021,** *23* (2), 253.

3. Ferrer, M.; Chernikova, T. N.; Timmis, K. N.; Golyshin, P. N., Expression of a temperature-sensitive esterase in a novel chaperone-based Escherichia coli strain. *Appl Environ. Microbiol* **2004,** *70* (8), 4499.

4. Joseph, R. E.; Andreotti, A. H., Bacterial expression and purification of interleukin-2 tyrosine kinase: single step separation of the chaperonin impurity. *Protein. Expr. Purif* **2008,** *60* (2), 194.

5. Cabrita, L. D.; Gilis, D.; Robertson, A. L.; Dehouck, Y.; Rooman, M.; Bottomley, S. P., Enhancing the stability and solubility of TEV protease using in silico design. *Protein Sci* **2007,** *16* (11), 2360.

6. Huo, L.; Zhao, X.; Acedo, J. Z.; Estrada, P.; Nair, S. K.; van der Donk, W. A., Characterization of a dehydratase and methyltransferase in the biosynthesis of ribosomally synthesized and post-translationally modified peptides in Lachnospiraceae. *ChemBioChem* **2020,** *21* (1-2), 190.

7. Hudson, G. A.; Zhang, Z.; Tietz, J. I.; Mitchell, D. A.; van der Donk, W. A., In vitro biosynthesis of the core scaffold of the thiopeptide thiomuracin. *J. Am. Chem. Soc.* **2015,** *137* (51), 16012.

8. Morrison, P. M.; Foley, P. J.; Warriner, S. L.; Webb, M. E., Chemical generation and modification of peptides containing multiple dehydroalanines. *Chem. Commun.* **2015,** *51* (70), 13470.

9. Brademan, D. R.; Riley, N. M.; Kwiecien, N. W.; Coon, J. J., Interactive peptide spectral annotator: A versatile web-based tool for proteomic applications*. *Mol. Cell. Proteom.* **2019,** *18* (8, Supplement 1), S193.

10. Niedermeyer, T. H.; Strohalm, M., mMass as a software tool for the annotation of cyclic peptide tandem mass spectra. *PLoS One* **2012,** *7* (9), e44913.

11. Lundblad, J. R.; Laurance, M.; Goodman, R. H., Fluorescence polarization analysis of protein-DNA and protein-protein interactions. *J. Mol. Endocrinol.* **1996,** *10* (6), 607.

12. Munson, P. J.; Rodbard, D., An exact correction to the "cheng-prusoff" correction. *J. Recept. Res.* **1988,** *8* (1-4), 533.

13. Paizs, B.; Suhai, S., Fragmentation pathways of protonated peptides. *Mass Spectrom. Rev* **2005,** *24* (4), 508.

14. Kaufmann, R.; Kirsch, D.; Spengler, B., Sequenching of peptides in a time-of-flight mass spectrometer: evaluation of postsource decay following matrix-assisted laser desorption ionisation (MALDI). *Int. J. Mass Spectrom. Ion Processes* **1994,** *131*, 355.

15. Cohen, S. L., Ozone in ambient air as a source of adventitious oxidation. a mass spectrometric study. *Anal. Chem.* **2006,** *78* (13), 4352.

16. Zhang, Z.; Hudson, G. A.; Mahanta, N.; Tietz, J. I.; van der Donk, W. A.; Mitchell, D. A., Biosynthetic timing and substrate specificity for the thiopeptide thiomuracin. *J. Am. Chem. Soc.* **2016,** *138* (48), 15511.
